# Effect of Costimulatory Blockade With Abatacept After Ustekinumab Withdrawal in Patients With Moderate to Severe Plaque Psoriasis: The PAUSE Randomized Clinical Trial

**DOI:** 10.1001/jamadermatol.2021.3492

**Published:** 2021-10-13

**Authors:** Kristina M. Harris, Dawn E. Smilek, Margie Byron, Noha Lim, William T. Barry, James McNamara, Sandra Garcet, Robert J. Konrad, Martin Stengelin, Pradeepthi Bathala, Neil J. Korman, Steven R. Feldman, Erin E. Boh, Kirk Barber, Anne E. Laumann, Yolanda Rosi Helfrich, Gerald G. Krueger, Howard Sofen, Robert Bissonnette, James G. Krueger

**Affiliations:** 1 Biomarker and Discovery Research, Immune Tolerance Network, University of California, San Francisco, San Francisco; 2 Clinical Trials Group, Clinical and Translational Medicine, Immune Tolerance Network, University of California, San Francisco, San Francisco; 3 Rho Inc, Durham, North Carolina; 4 Autoimmunity and Mucosal Immunology Branch, Division of Allergy, Immunology, and Transplantation/National Institute of Allergy and Infectious Diseases, Rockville, Maryland; 5 The Rockefeller University, New York, New York; 6 Lilly Research Laboratories, Eli Lilly and Company, Indianapolis, Indiana; 7 Meso Scale Diagnostics LLC, Rockville, Maryland; 8 Department of Dermatology, University Hospitals Cleveland Medical Center, Cleveland, Ohio; 9 Department of Dermatology, Wake Forest School of Medicine, Winston-Salem, North Carolina; 10 Health Sciences Center, Tulane University School of Medicine, New Orleans, Louisiana; 11 Department of Medicine (Dermatology), University of Calgary, Calgary, Alberta, Canada; 12 Department of Dermatology, Northwestern University, Colorado Springs, Colorado; 13 Dermatology Clinic, University of Michigan Medicine, Ann Arbor; 14 Department of Dermatology, University of Utah School of Medicine, Salt Lake City; 15 Dermatology, David Geffen UCLA (University of California, Los Angeles) School of Medicine, Los Angeles; 16 Innovaderm, Montreal, Quebec, Canada

## Abstract

**Importance:**

Psoriasis relapse may involve compensatory T-cell activation pathways in the presence of CD28-CD80/CD86 blockade with abatacept.

**Objective:**

To determine whether costimulatory signaling blockade with abatacept prevents psoriasis relapse after ustekinumab withdrawal.

**Design, Setting, and Participants:**

Psoriasis Treatment with Abatacept and Ustekinumab: a Study of Efficacy (PAUSE), a parallel-design, double-blind, placebo-controlled randomized clinical trial, was conducted at 10 sites in the US and Canada. Participant enrollment opened on March 19, 2014, and concluded on April 11, 2016. Participants were adults with moderate to severe plaque psoriasis and received ustekinumab in a lead-in phase. Those who responded to ustekinumab at week 12 were randomized 1:1 to either the continued with ustekinumab group (ustekinumab group) or the switched to abatacept group (abatacept group). Treatment was discontinued at week 39, and participants were followed up for psoriasis relapse until week 88. Statistical analyses were performed in the intention-to-treat (ITT) and safety samples from May 3, 2018, to July 6, 2021.

**Interventions:**

Participants received subcutaneous ustekinumab at weeks 0 and 4 (45 mg per dose for those ≤100 kg; 90 mg per dose for those >100 kg). Participants randomized to the abatacept group at week 12 received subcutaneous abatacept, 125 mg weekly, from weeks 12 to 39 and ustekinumab placebo at weeks 16 and 28. Participants randomized to the ustekinumab group received ustekinumab at weeks 16 and 28 and abatacept placebo weekly from weeks 12 to 39.

**Main Outcomes and Measures:**

The primary end point was the proportion of participants with psoriasis relapse (loss of ≥50% of the initial Psoriasis Area and Severity Index improvement) between weeks 12 and 88. Secondary end points included time to psoriasis relapse, proportion of participants with psoriasis relapse between weeks 12 and 40, and adverse events. The psoriasis transcriptome and serum cytokines were evaluated.

**Results:**

A total of 108 participants (mean [SD] age, 46.1 [12.1] years; 73 [67.6%] men) were treated with open-label ustekinumab; 91 were randomized to blinded treatment. Similar proportions of participants in the abatacept group and the ustekinumab group relapsed between weeks 12 and 88 (41 of 45 [91.1%] vs 40 of 46 [87.0%]; *P* = .41). Median time to relapse from the last dose of ustekinumab was similar between groups as well: 36 weeks (95% CI, 36-48 weeks) in the abatacept group vs 32 weeks (95% CI, 28-40 weeks) in the ustekinumab group. Similar numbers and rates of adverse events occurred. Abatacept did not maintain suppression of the pathogenic IL-23-mediated psoriasis molecular signature in lesions after ustekinumab withdrawal, and serum IL-19 levels increased.

**Conclusions and Relevance:**

This parallel-design, double-blind randomized clinical trial found that abatacept did not prevent psoriasis relapse that occurred after ustekinumab withdrawal because it did not completely block the pathogenic psoriasis molecular pathways that led to relapse.

**Trial Registration:**

ClinicalTrials.gov Identifier: NCT01999868

## Key Points

**Question:**

Does blockade of CD28/B7 costimulatory signaling with abatacept suppress the psoriasis molecular signature and prevent psoriasis relapse after ustekinumab withdrawal?

**Findings:**

In this parallel-design, double-blind randomized clinical trial of 91 adults with moderate to severe plaque psoriasis, costimulatory blockade with abatacept did not prevent psoriasis relapse and did not maintain suppression of the pathogenic psoriasis molecular signature following ustekinumab withdrawal.

**Meaning:**

In this study, abatacept did not prevent psoriasis relapse, which may rely on alternative, compensatory mechanisms of residual T-cell activation in skin.

## Teaser

This parallel-design, double-blind randomized clinical trial evaluates whether abatacept could prevent psoriasis relapse after discontinuation of ustekinumab treatment in adults with plaque psoriasis.

## Introduction

Psoriasis vulgaris is a systemic immune-mediated disease that predominantly involves the skin and joints. The pathogenesis involves activated interkeukin (IL)–17–producing T cells in skin, perpetuated by an IL-23–mediated psoriasis molecular signature that reflects the pathogenic keratinocyte response.^1,2,3^ Ustekinumab is a US Food and Drug Administration–approved biologic agent for psoriasis that targets the IL-12/IL-23 pathways. Ongoing administration of ustekinumab is required because discontinuation leads to psoriasis relapse.^4,5^

T-cell activation depends on both antigen-specific engagement of the T-cell receptor and costimulation by antigen-presenting cells. The CD28 protein induces a critical costimulatory signal in T cells on ligation of CD80/CD86. Antigen engagement of the T-cell receptor in the absence of CD28-mediated costimulation may lead to T-cell tolerance,^6^ providing a rationale for costimulatory blockade as a therapeutic strategy in autoimmunity.^7,8,9,10,11^

Abatacept (CTLA4-Ig) is a costimulatory-blocking fusion protein that consists of the extracellular domain of the CTLA4 ligand for CD80/CD86 coupled to a modified Fc portion of human IgG. Abatacept acts by competing for CD80/CD86 binding to CD28 on T cells, thereby inhibiting T-cell activation and function. Abatacept had a treatment effect during an early-phase psoriasis trial^12^ and improved skin lesions in a phase 2 psoriatic arthritis trial.^13^ Therefore, it was hypothesized that costimulatory blockade with abatacept could induce tolerance in pathogenic T cells encountering antigen in resolving psoriasis lesions, leading to long-term remission.

The Psoriasis Treatment with Abatacept and Ustekinumab: a Study of Efficacy (PAUSE) trial was conducted to determine whether blockade of costimulatory signaling with abatacept could prevent psoriasis relapse after ustekinumab withdrawal. Longitudinal evaluation of the disease transcriptome in lesional skin and serum cytokines was performed to identify the mechanisms associated with treatment outcomes.

## Methods

### Study Design

PAUSE was a multicenter parallel-design, double-blind, placebo-controlled randomized clinical trial that was conducted at 10 investigational sites in the United States and Canada (US: Dermatology Research Associates, Los Angeles, California; Northwestern University, Chicago, Illinois; Tulane University School of Medicine, New Orleans, Louisiana; University of Michigan, Ann Arbor; The Rockefeller University, New York, New York; Wake Forest University, Winston-Salem, North Carolina; Case Western University, Cleveland, Ohio; and University of Utah, Salt Lake City; Canada: Kirk Barber Research, Calgary, Alberta, and Innovaderm Research Inc, Montreal, Quebec). Enrollment opened on March 19, 2014, and concluded on April 11, 2016. The trial was conducted in compliance with the Declaration of Helsinki^14^ and was approved by the institutional review boards at all of the investigational sites. All participants provided written informed consent. We followed the Consolidated Standards of Reporting Trials (CONSORT) reporting guideline. The trial protocol is available in Supplement 1.

**Figure 1.**
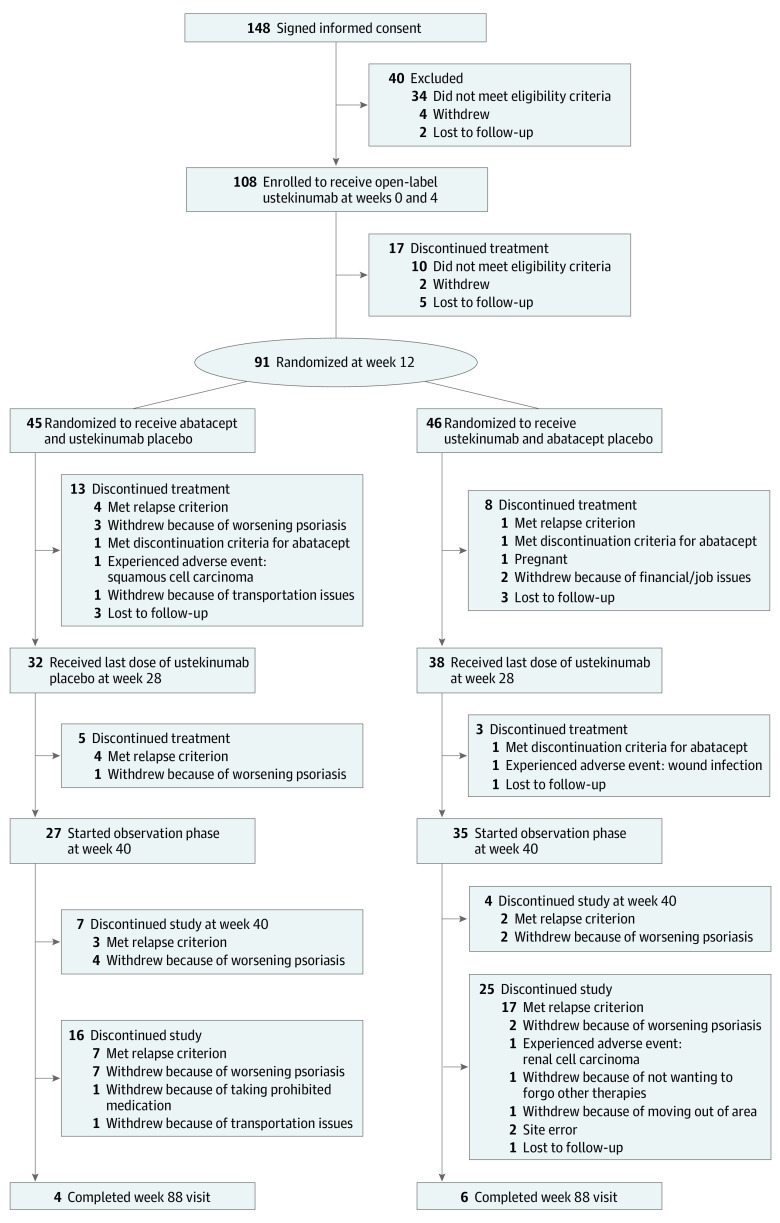
CONSORT Diagram

The trial consisted of a lead-in phase (weeks 0 to 12), a randomized treatment phase (weeks 12 to 40), and an observation phase (weeks 40 to 88) (eFigure 1 in Supplement 2). During the lead-in phase, 108 participants with moderate to severe psoriasis vulgaris received subcutaneous ustekinumab at weeks 0 and 4. Participants who weighed 100 kg or less received 45 mg per dose, and participants who weighed more than 100 kg received 90 mg per dose. At week 12, participants’ response to ustekinumab was assessed using the Psoriasis Area and Severity Index (PASI), and participants were eligible for randomization if they achieved 75% or greater improvement from their baseline PASI (PASI 75). Ninety-one participants were randomized 1:1 to blinded treatment (Figure 1), either continued ustekinumab (ustekinumab group) or switched to abatacept (abatacept group), using a stratified, permuted block design. Randomization was stratified by baseline PASI of 12 to 20 vs greater than 20, and sites used a secure interactive web response system that was maintained at the Statistical and Clinical Coordinating Center (Rho Inc). The abatacept group received subcutaneous abatacept, 125 mg, which was administered weekly from weeks 12 to 39, and ustekinumab placebo at weeks 16 and 28. The ustekinumab group received ustekinumab at weeks 16 and 28 and abatacept placebo weekly from weeks 12 to 39. The PASI was assessed every 4 weeks until the final study visit.

Abatacept and abatacept placebo were self-administered by participants with syringes that were provided by Bristol Myers Squibb. Ustekinumab vials were purchased from Janssen Pharmaceuticals Inc. Ustekinumab syringes and normal saline placebo syringes were prepared by unblinded site pharmacists and administered by blinded site personnel. Other biologic, immunosuppressive, and topical agents for psoriasis were prohibited.

### Study Population, End Points, and Assessments

Eligible participants were 18 to 65 years of age with a diagnosis of moderate to severe plaque psoriasis. Race and ethnicity of participants were self-reported or investigator observed. American Indian or Alaska Native, Native Hawaiian or Other Pacific Islander, and other race and ethnicity categories were combined into 1 category as an extra measure to protect the identity of 1 participant. The multiple race category was not individually specified for the same reason.

The participants had a PASI of 12 or greater, and 10% or more of their body surface area was affected by psoriasis. Exclusion criteria included previous treatment with ustekinumab or other agents that targeted IL-12 or IL-23, moderate to severe psoriatic arthritis, and comorbid conditions that conferred an increased risk of study participation.

The primary end point was the proportion of participants with psoriasis relapse between weeks 12 and 88. Psoriasis relapse was defined as loss of 50% or more of the initial PASI improvement at week 12 compared with baseline scores. Secondary end points included the time to psoriasis relapse, the proportion of participants with psoriasis relapse occurring between weeks 12 and 40, and the frequency and severity of adverse events.

### Transcriptomics of Skin Biopsies and Serum Cytokines

Skin biopsies were obtained from an active lesion and a nonlesional area at week 0 and stored in RNAlater (Ambion Inc) at −70 to −80 °C. The same lesion was resampled at weeks 12, 24 (optional), and 40 and at the final study visit, and RNA was isolated using RNeasy kits (Qiagen). Samples were analyzed using RNA sequencing (HiSeq 4000 [Illumina], paired-end 100 base pairs [bp] × 2 cycle, polyA selection of total stranded RNA) at a sequencing depth of approximately 47 million reads per sample. Samples were aligned to the human genome build hg38 using the R package Rsubread,^15^ and aligned sequences were counted using the featureCounts with hg38 annotation.^16^ For downstream analysis, gene counts were normalized using the variance-stabilizing transformation method in the DESeq2 package.^17^ Differentially expressed genes in paired samples were determined using cutoffs of fold change (≥1.5) and a false discovery rate less than 0.05. Serum samples were collected before and after treatment, and cytokines were quantified using an ultrasensitive immunoassay for low abundant proteins (Meso Scale Diagnostics LLC). Assays were performed in a 96-well plate format and read on a highly sensitive imaging detection system (MESO SECTOR Imager; Meso Scale Diagnostics LLC). Analyte concentrations were calculated using a weighted 4-parameter logistic fit. Concentrations below the limits of blank were assigned the limit of blank (eTable 5 in Supplement 2). Serum IL-19 was quantified by immunoassay, as previously described.^18^ Samples were also analyzed from ACCLAIM (A Cooperative Clinical Study of Abatacept in Multiple Sclerosis), a placebo-controlled trial of abatacept in multiple sclerosis.^19,20^

### Statistical Analysis

Statistical analyses were performed in the intention-to-treat (ITT) sample (defined as all participants who were eligible at week 12 and randomized to 1 of the blinded treatment groups) and in the safety sample (defined as all participants who were receiving ≥1 dose of study treatment).

For the primary end point, treatment group comparisons for the proportion of ITT participants who were experiencing a psoriasis relapse between weeks 12 and 88 were performed using a logistic regression model with the participant’s relapse status as the dependent variable and treatment group as the independent variable. Covariates were randomization stratum (baseline PASI of 12-20 vs >20) and duration of disease. Those who terminated participation early because they had met the psoriasis relapse criterion or because of worsening psoriasis were classified as having experienced a relapse. Those who terminated participation early for any other reason were classified as dropouts. The primary analysis categorized dropouts as experiencing a relapse. In sensitivity analyses, dropouts were assumed to have not experienced a relapse and were removed from analysis for having a missing relapse status.

Based on PHOENIX 1 (A Study of Safety and Effectiveness of Ustekinumab [CNTO 1275] in Patients With Moderate to Severe Plaque-type Psoriasis),^4^ we expected 80% of participants who were randomized to the ustekinumab group to experience a psoriasis relapse by week 88. We hypothesized a decrease in psoriasis relapse rate of 50% by week 88 in participants who were randomized to the abatacept group. Assuming 1:1 randomization and a 2-sided type I error rate of α = .05, a sample size of 39 randomized participants in each treatment group would provide 80% power for the primary end point analysis. It was anticipated that approximately two-thirds of participants who were receiving ustekinumab during the lead-in phase would achieve a PASI 75 improvement for randomization eligibility.^4,5^ This estimate resulted in a target sample size of 120 participants in expectation of randomizing 40 participants per treatment group.

Time to psoriasis relapse was evaluated in the ITT sample using nonparametric estimates of the survival function for interval-censored data,^21^ based on the scheduled PASI assessment at 4-week intervals. Ad hoc subgroup analyses were performed to evaluate the outcomes in participants who achieved and those who did not achieve PASI 90 (≥90% improvement from baseline) by week 12 and at any point in the study. Additional ad hoc survival analyses evaluated time from last ustekinumab dose until relapse in participants in the ITT sample who were completing treatment with the agent. The analyses included participants in the abatacept group who were completing the last dose of open-label ustekinumab at week 4 and participants in the ustekinumab group who were completing the last dose of blinded ustekinumab at week 28.

All treatment group comparisons were conducted using 2-sided statistical tests, and estimates are reported with 95% CIs. Two-sided *P* < .05 was considered significant. All analyses were performed using SAS, version 9.4 (SAS Institute Inc).

The RNA sequencing and serum cytokine data were analyzed using the mixed model for repeated measures, and *P* values were calculated by comparing least square means between treatment groups, skin biopsy types, or time points. The analyses were performed using the nlme package in R, version 4.0.2 (R Foundation for Statistical Computing). The RNA sequencing and serum cytokine analyses were performed from May 3, 2018, to July 6, 2021.

## Results

A total of 108 eligible participants with moderate to severe psoriasis vulgaris received ustekinumab at weeks 0 and 4 (Figure 1; eFigure 1 in Supplement 2). Participants had a mean (SD) age of 46.1 (12.1) years and were composed of 73 men (67.6%) and 35 women (32.4%). The self-reported or investigator-observed race and ethnicity of participants were as follows: Asian (4 [3.7%]), Black (6 [5.6%]), Hispanic or Latino (12 [11.1%]), multiple races (2 [1.9%]), not Hispanic or Latino (96 [88.9%]), other (1 [0.9%]), and White (95 [88.0%]) (eTable 1 in Supplement 2). At week 12, a total of 91 participants (84.3%) met the PASI 75 target after treatment with ustekinumab and were randomized. Characteristics did not significantly differ between randomized and nonrandomized participants, although mean (SD) body weight at screening was higher in those who were not randomized (104.5 [24.75] kg vs 96.2 [20.70] kg; *P* = .20).

### Psoriasis Relapse and Adverse Events

In the abatacept group, more participants experienced a psoriasis relapse before week 88 compared with participants in the ustekinumab group (41 of 45 [91.1%] vs 40 of 46 [87.0%]; *P* = .41) (Table). The psoriasis relapse rate did not decrease in the abatacept group compared with the ustekinumab group, and the primary end point of psoriasis relapse between weeks 12 and 88 was not met. A higher proportion of participants in the abatacept group relapsed between weeks 12 and 40 compared with participants in the ustekinumab group (25 of 45 [55.6%] vs 14 of 46 [30.4%]; *P* = .01).

**Table.** Psoriasis Relapse in the Intention-to-Treat Analysis Population

Variable	Abatacept group (n = 45)	Ustekinumab group (n = 46)
Primary analyses^a^		
Participants who experienced a relapse between wk 12 and wk 88		
No. of evaluable participants	45	46
No. of participants who relapsed (%)	41 (91.1)	40 (87.0)
*P* value: abatacept vs ustekinumab^b^	.41	
Key secondary analyses^a^		
Participants who experienced a relapse between wk 12 and wk 40		
No. of evaluable participants	45	46
No. of participants who relapsed (%)	25 (55.6)	14 (30.4)^c^
*P* value: abatacept vs ustekinumab^b^	.01	
Sensitivity analyses^d^		
Participants who experienced a relapse between wk 12 and wk 88		
No. of evaluable participants	38	30
No. of participants who relapsed (%)	34 (89.5)	24 (80.0)
*P* value: abatacept vs ustekinumab^b^	.16	
Participants who experienced a relapse between wk 12 and wk 40		
No. of evaluable participants	39	37
No. of participants who relapsed (%)	19 (48.7)	5 (13.5)
*P* value: abatacept vs ustekinumab^b^	.002	
Additional secondary analyses		
Time to psoriasis relapse from enrollment (wk 0)		
No. of evaluable participants	45	46
No. of participants who relapsed (%)	34 (75.6)	24 (52.2)
Median time to relapse (95% CI), wk	40 (40-52)	60 (56-68)
Exploratory post hoc analyses		
Time to psoriasis relapse from last ustekinumab dose		
No. of evaluable participants	45	36
No. of participants who relapsed (%)	34 (75.6)	23 (63.9)
Median time to relapse (95% CI), wk	36 (36-48)	32 (28-40)

^a^ Primary and key secondary analyses of end points considered participants who dropped out as having relapsed.

^b^ The *P* value was calculated from a χ^2^ test comparing the treatment arms in a logistic regression model with the participant's relapse status as the dependent variable and with randomization stratum (low vs high Psoriasis Area and Severity Index) and duration of psoriasis disease before screening as the covariates.

^c^ One participant in the ustekinumab group discontinued treatment early for wound infection but continued to be followed up for relapse.

^d^ Sensitivity analyses of end points excluded participants who dropped out without evidence of relapse or disease worsening.

For the primary analysis, all discontinued participants were characterized as having a psoriasis relapse. Discontinuation for reasons other than worsening psoriasis or protocol-defined psoriasis relapse occurred in 23 of 91 participants (25.3%) or dropouts. Sensitivity analyses conducted among participants who were not dropouts confirmed the primary analysis results. Relapse occurred between weeks 12 and 88 in 34 of 38 participants (89.5%) in the abatacept group who were not dropouts compared with 24 of 30 (80.0%) in the ustekinumab group (*P* = .16).

**Figure 2.**
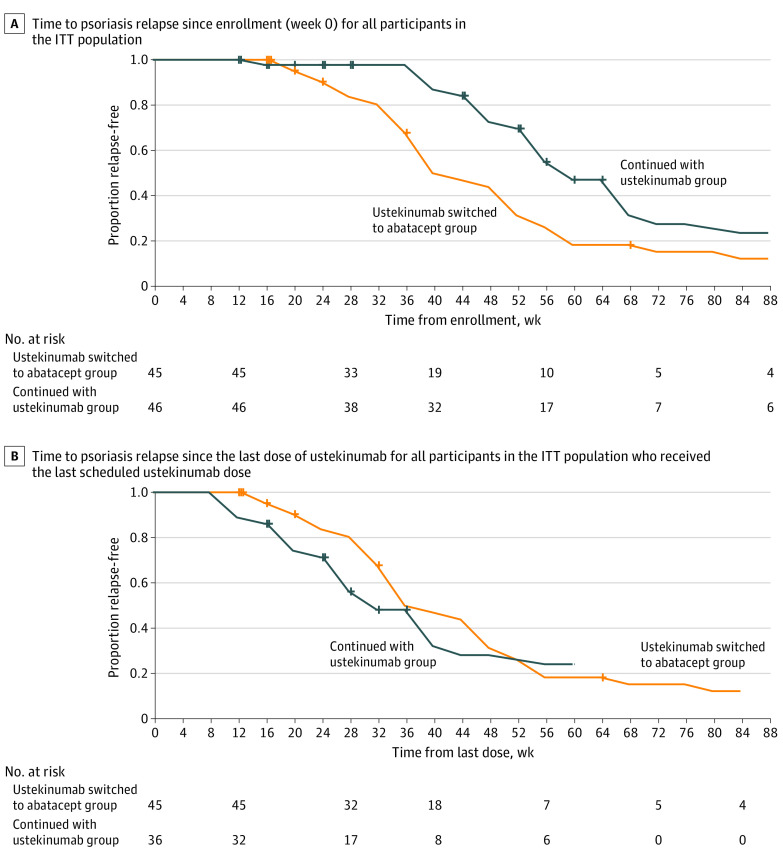
Time to Psoriasis Relapse Time to psoriasis relapse curves for the switched to abatacept group (abatacept group) and the continued with ustekinumab group (ustekinumab group) are displayed as the estimated survival function for interval-censored data. For participants who experienced a psoriasis relapse, the censoring interval was derived from the time points of the participants’ last 2 Psoriasis Area and Severity Index (PASI) evaluations, which were typically 4 weeks apart. For participants who did not experience a psoriasis relapse, the time to relapse was right-censored at the time point of the last PASI evaluation. For participants randomized to the abatacept group, the last dose of ustekinumab was administered at week 4. For participants randomized to the ustekinumab group, the last dose of ustekinumab was administered at week 28. ITT indicates intention to treat.

The median time to relapse from enrollment was 40 weeks (95% CI, 40-52 weeks) in the abatacept group and 60 weeks (95% CI, 56-68 weeks) in the ustekinumab group (Figure 2A). However, the median time to relapse from the last dose of ustekinumab was similar between the 2 groups: 36 weeks (95% CI, 36-48 weeks) in the abatacept group and 32 weeks (95% CI, 28-40 weeks) in the ustekinumab group (Figure 2B).

A longer time to psoriasis relapse was associated with a maximum PASI improvement of 90% or greater in both groups (eFigure 2 in Supplement 2) but was not associated with body weight or PASI at baseline. The number of participants who experienced treatment-emergent adverse events (28 of 45 [62.2%] vs 22 of 46 [47.8%]) and serious adverse events (2 of 45 [4.4%] vs 5 of 46 [10.9%]) was similar between the abatacept and ustekinumab groups (eTable 2 in Supplement 2). The adverse event rates were also similar (59 [1.995 person-years] vs 59 [1.601 person-years]). Serious adverse events are summarized in eTables 3 and 4 in Supplement 2.

### Modulation of the IL-23–Mediated Psoriasis Molecular Signature in Skin by Ustekinumab

**Figure 3.**
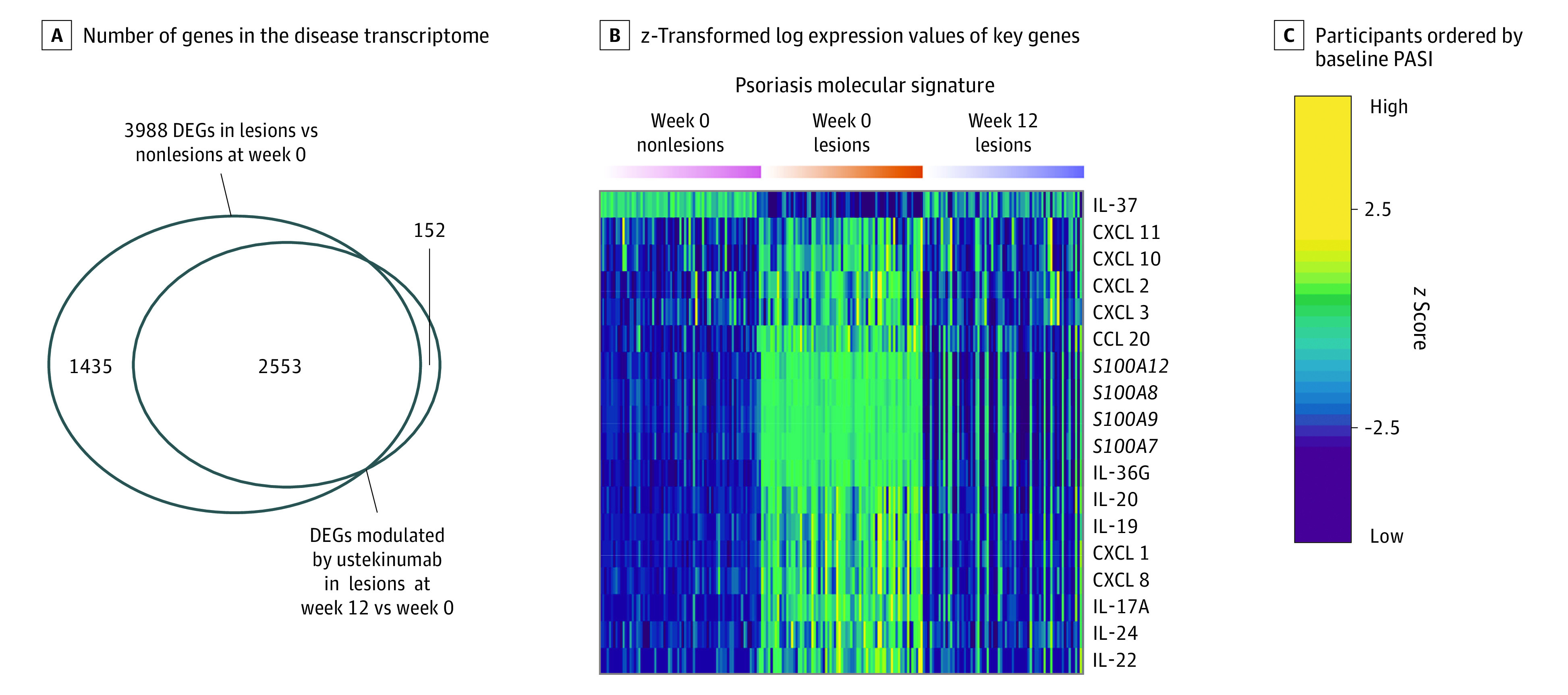
Modulation of the IL-23–Mediated Psoriasis Molecular Signature Genes in Participants Who Were Eligible for Randomization (≥PASI 75) A, Venn diagram shows the number of genes in the disease transcriptome (lesions vs nonlesions at week 0) and not in the disease transcriptome that were significantly modulated by ustekinumab (at week 12: fold change, ≥1.5; false discovery rate <0.05). B, Heat map shows *z*-transformed log expression values of key genes in the interleukin (IL)–23–mediated psoriasis molecular signature in paired nonlesions (pink group; left) and lesions (orange group; center) at week 0 and resolving lesions (indigo group; right) at week 12. C, Individual participant data are ordered by baseline Psoriasis Area and Severity Index (PASI) from low to high for the 3 groups. The *z* score indicates the number of SDs higher or lower than the mean expression value for each gene. DEG indicates differentially expressed gene.

Analysis of RNA sequencing was performed using total RNA from lesional and nonlesional skin specimens that were collected at week 0 to define the baseline psoriasis disease transcriptome in participants who were eligible for randomization. The baseline disease transcriptome comprised 3988 differentially expressed genes (Figure 3A; eFigure 3A and B in Supplement 2; eTable 6 in Supplement 3). In resolving lesions at week 12, we found 2705 genes that were modulated by ustekinumab compared with paired active lesions at week 0, and 2553 of these genes were in the disease transcriptome (Figure 3A; eFigure 3C in Supplement 2; eTable 7 in Supplement 4). Consistent with its mechanism of action, ustekinumab improved disease-associated genes that were modulated by IL-23, including IL-17A and the pathogenic psoriasis molecular signature genes that were induced in keratinocytes by IL-17 receptor signaling^1,2,22^ (Figure 3B; eFigure 3C in Supplement 2; eTable 7 in Supplement 4). In addition, ustekinumab downmodulated a number of transcripts associated with CD28-CD80/CD86 pathway targets of abatacept therapy, including CD80, CD28, ICOS, and CTLA-4 (eFigure 4 in Supplement 2).

### Modulation of Relevant Cytokines in Serum by Ustekinumab

**Figure 4.**
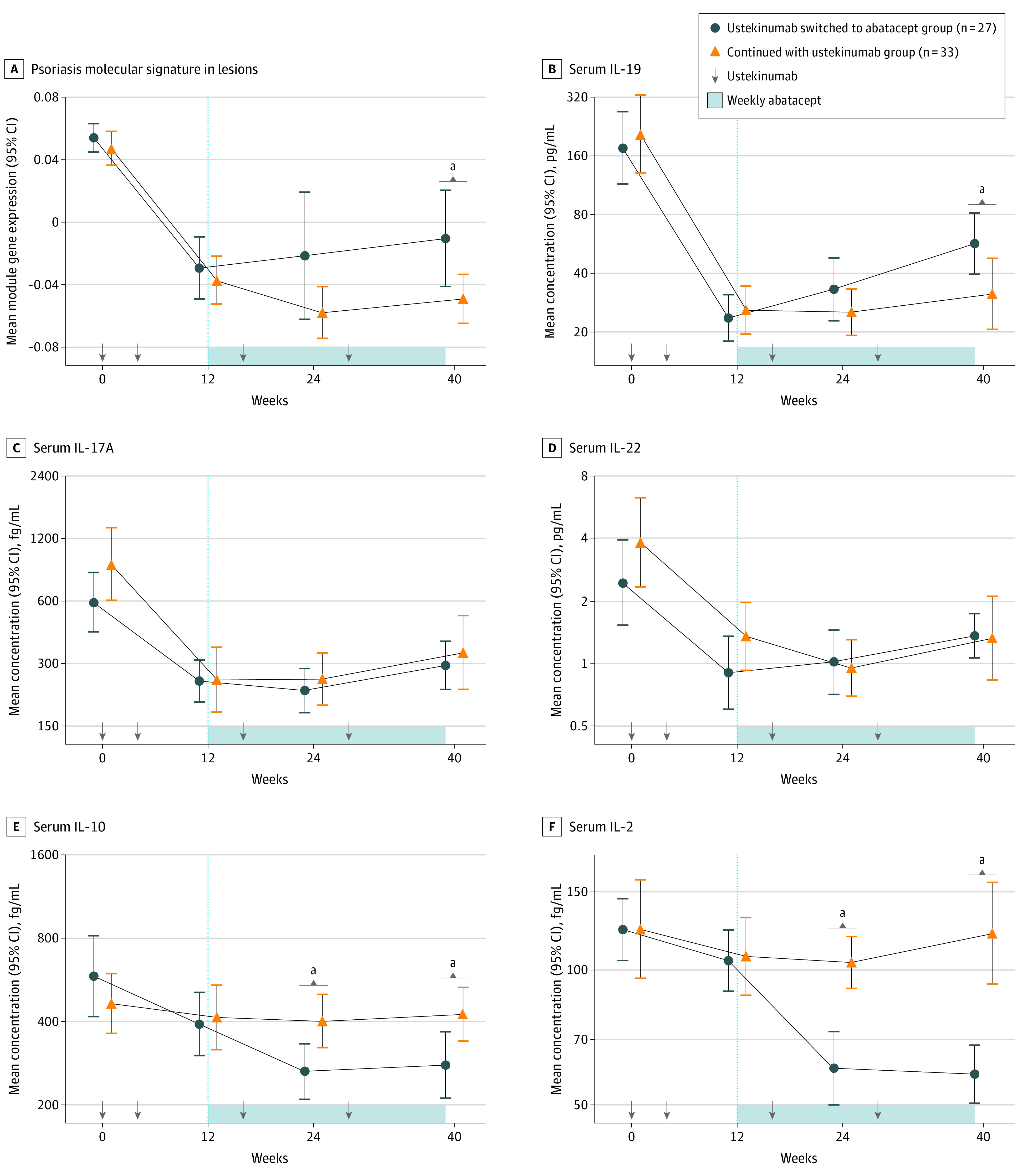
Suppression of the Psoriasis Molecular Signature in Resolving Lesions and Serum IL-19 Levels After Ustekinumab Withdrawal in Participants Who Completed Treatment A, Line plot shows the eigengene (weighted mean expression of genes in the psoriasis molecular signature module) value for lesions at week 0 and resolving lesions at weeks 12, 24, and 40 by treatment group. B to F, Line plots show the mean concentration of interleukin (IL)–19, IL-17A, IL-22, IL-10, and IL-2 levels in serum at weeks 0, 12, 24, and 40 by treatment group. Error bars display the 95% CIs. *P* values between treatment groups were determined by mixed model for repeated measures with baseline adjustment. ^a^Significant difference (*P* < .05) between treatment groups.

Interleukin 17A, IL-22, and IL-19 are elevated in both active skin lesions and blood in psoriasis and have been associated with disease activity.^18,23,24^ Ustekinumab inhibits secretion of IL-17A and other cytokines in vitro,^25^ but ex vivo studies from clinical trial specimens have been limited by assay detection limits for low abundant serum proteins. We evaluated serum levels of IL-17A and other cytokines, including IL-22 and IL-19,^18^ from the psoriasis molecular signature. Consistent with its effect on IL-17A (566 fg/mL; 95% CI, 509-612 fg/mL; *P* < .001), IL-19 (191 pg/mL; 95% CI, 183-197 pg/mL; *P* < .001), and IL-22 (2.4 pg/mL; 95% CI, 2.1-2.7 pg/mL; *P* < .001) transcripts in resolving lesions (Figure 3B), ustekinumab significantly reduced the levels of these cytokines in serum at week 12 vs week 0 (eFigure 3D in Supplement 2; Figure 4). A modest reduction was also observed for serum levels of interferon γ, IL-2, IL-10, IL-6, tumor necrosis factor, and thymic stromal lymphopoietin (eFigure 3D in Supplement 2).

### Maintaining Suppression of Psoriasis Molecular Signature in Resolving Lesions and IL-19 in Serum

Given that sequential treatment with abatacept after ustekinumab did not prevent relapse, we asked whether suppression of the pathogenic, IL-23-mediated psoriasis molecular signature in resolving lesions^1,2,22^ and relevant serum cytokines was maintained in participants in the abatacept group. The weighted mean expression of genes in the psoriasis molecular signature was compared between groups of participants who completed blinded treatment through week 39. Treatment with ustekinumab resulted in approximately 2-fold reduction of the psoriasis molecular signature genes in resolving lesions at week 12 (Figure 4A). After randomization, suppression of the psoriasis molecular signature (Figure 4A) and IL-17A transcripts in skin (eFigure 5 in Supplement 2) was not maintained in the abatacept group vs the ustekinumab group at week 24 and/or week 40; this result is consistent with the earlier relapse time from enrollment in the abatacept group vs the ustekinumab group (Figure 2A). In addition, suppression of serum IL-19 levels at week 12 was not maintained at week 40 in the abatacept group vs the ustekinumab group (27 pg/mL; 95% CI, 8-57 pg/mL; *P* = .008) (Figure 4B). In contrast, serum IL-17A and IL-22 levels were similar between the groups at the time points evaluated (Figure 4C and D), which differed from the transcripts in skin at these time points (eFigure 5 in Supplement 2).

### Reduction of Serum Cytokines Associated With Regulatory T Cells by Abatacept

Given that ustekinumab downregulated molecular targets of abatacept in resolving lesions (eFigure 4 in Supplement 2) and that sequential treatment with abatacept did not prevent psoriasis relapse or maintain improvement in clinically relevant biomarkers, we investigated other immunological effects in the abatacept group. The CD28 costimulatory pathway is important to the development and maintenance of regulatory CD4^+^Foxp3^+^ T cells (Tregs), which function to maintain immune tolerance, and CD4^+^ follicular helper T cells that promote antibody-secreting cells and memory B cells.^26,27,28^ Abatacept reduces the frequency of circulating effector Tregs and follicular helper T cells by disrupting the molecular pathways that are important for their proliferation and maintenance.^20,29,30,31^ Given that IL-2 is indispensable for Treg survival, and IL-10 is produced by Tregs,^32,33,34,35^ we postulated these cytokines were decreased in the abatacept group. Serum samples from participants who completed blinded treatment in the placebo-controlled ACCLAIM trial of abatacept for multiple sclerosis^19,20^ were analyzed as a confirmatory study cohort. Serum IL-10 (week 24: 166 fg/mL [95% CI, 83-227 fg/mL; *P* = .002]; week 40: 179 fg/mL [95% CI, 93-243 fg/mL; *P* = .002]) and IL-2 (week 24: 43 fg/mL [95% CI, 26-56 fg/mL; *P* < .001]; week 40: 62 fg/mL [95% CI, 46-75 fg/mL; *P* < .001]) levels were reduced at weeks 24 and 40 among participants in the abatacept group vs those in the ustekinumab group (Figure 4E and F), which is consistent with results from the ACCLAIM study (eFigure 6 in Supplement 2) and confirms the decrease of serum cytokines associated with Tregs in both trials. IL-10 and IL-2 transcripts in lesions exhibited a downward trend from week 12 in the abatacept group, but they did not differ significantly between groups (eFigure 5 in Supplement 2).

## Discussion

Early trials with abatacept in psoriasis and psoriatic arthritis suggested clinical benefit in cutaneous lesions,^12,13^ contributing to the idea that switching from ustekinumab to abatacept in psoriasis might induce T-cell tolerance in resolving lesions by means of costimulatory blockade.^6,7^ However, the results of PAUSE as reported here showed that abatacept did not prevent relapse after ustekinumab withdrawal. Most participants in both treatment groups experienced a psoriasis relapse or dropped out between weeks 12 and 88. Although abatacept is approved for psoriatic arthritis, the results of this trial do not support abatacept as a choice for treating psoriatic arthritis and psoriasis skin lesions concurrently.

We sought to reconcile these findings with those in previous studies that showed some clinical benefit of abatacept in active psoriasis.^12^ The skin transcriptomic analysis revealed that key molecular targets of abatacept were downmodulated in resolving lesions following successful ustekinumab treatment. This result likely reflects a reduction in the pathogenic T cell–mediated pathways in resolving lesions that were modulated by abatacept in the context of active disease.^12,36^ Given these results, it is not surprising that abatacept did not delay relapse or maintain suppression of the IL-23–mediated psoriasis molecular signature after ustekinumab withdrawal. The findings suggested that the molecular trigger of psoriasis relapse may not completely rely on the CD28-CD80/CD86 costimulation pathway and/or may be dependent on compensatory T-cell activation pathways in the presence of abatacept.

A growing body of literature indicates that relapse is initiated by tissue-resident memory T (TRM) cells that are poised to produce IL-17A and IL-22 in resolving lesions.^37,38,39,40,41,42^ CD28 expression is highly variable in TRM cells and not present on most CD8^+^ TRM cells in healthy skin.^43^ Therefore, it is plausible that psoriasis relapse is triggered by CD28-independent, compensatory activation of IL-17A-producing CD8^+^ TRM cells that reignite the psoriasis molecular signature. Targeting alternative costimulatory molecules, such as 4-BBL or others in the tumor necrosis factor superfamily of receptors and ligands, may be a treatment option for preventing psoriasis relapse.^44,45^ Understanding the mechanisms that regulate the reactivation of TRM cells and their key interactions with innate immune and stromal cell populations in recurring lesions may help identify new treatment strategies for durable remission in psoriasis and potentially other diseases.

### Limitations

This study has some limitations. The abatacept dose may have been too low and/or administered too late after ustekinumab induction therapy to prevent psoriasis relapse. Research studies were limited by the number and frequency of paired skin and blood collections for analyses. Addition of a randomized double-placebo group would have clarified the immunological effects induced by abatacept treatment vs ustekinumab withdrawal.

## Conclusions

The PAUSE randomized clinical trial found that costimulatory blockade with abatacept did not prevent psoriasis relapse after ustekinumab withdrawal. Evaluation of the pathogenic psoriasis molecular signature demonstrated that clinical relapse may involve compensatory T-cell activation pathways in the presence of CD28-CD80/CD86 blockade.

## Notes

Supplement 1.
Trial Protocol

Supplement 2. **eFigure 1.** Study Design **eTable 1.** Demographic and Clinical Characteristics **eTable 2.** Summary of Treatment Emergent Adverse Events after Randomization (Safety Analysis Set) **eTable 3.** Summary of Preferred Terms for Serious Adverse Events in the Lead‐In Phase (Safety Analysis Set) **eTable 4.** Summary of Preferred Terms for Serious Adverse Events in the Post‐Randomization Phase (Safety Analysis Set) **eFigure 2.** Time to Psoriasis Relapse (ITT Population) **eFigure 3.** Modulation of the Disease Transcriptome (PSTR) in Skin and Relevant Cytokines in Serum in Participants Eligible for Randomization (≥PASI75) **eFigure 4.** Ustekinumab Modulates Targets of Abatacept in Lesional Skin **eFigure 5.** Abatacept Fails to Maintain Suppression of IL17A in Resolving Lesions Following Ustekinumab Withdrawal in Participants That Completed Study Treatment **eFigure 6.** Treatment Effect of Abatacept on Serum IL‐10 and IL‐2 in PAUSE Is Consistent With ACCLAIM **eTable 5.** Limits of Blank (LOB) and Lower Limit of Quantitation (LLOQ) of the MSD, LLC Multi‐plex Immunoassay

Supplement 3. **eTable 6.** DEGs in Baseline PSTR (Non‐lesional vs. Lesional at Week 0)

Supplement 4. **eTable 7.** DEGs in Active Lesions Versus Resolving Lesions at Week 12

Supplement 5.
Data Sharing Statement
