# Supplementary material for: Effect of Costimulatory Blockade With Abatacept After Ustekinumab Withdrawal in Patients With Moderate to Severe Plaque Psoriasis: The PAUSE Randomized Clinical Trial
Source: JAMA Dermatol. 2021 Oct 13;157(11):1–11. doi: 10.1001/jamadermatol.2021.3492 (PMC8515260; doi:10.1001/jamadermatol.2021.3492)
Supplement: Supplement 1. — Trial Protocol [file jamadermatol-e213492-s001.pdf]

Immune
Tolerance
Network

Efficacy of Ustekinumab (Anti-IL-12/23) followed by Abatacept (CTLA4-Ig) for the Treatment of Psoriasis Vulgaris

Protocol ITN059AI

Version 4.0 (February 26, 2015)

IND # 117,633

Protocol Chair

James Krueger, MD, PhD
Professor and Head
Laboratory of Investigative Dermatology
The Rockefeller University
1230 York Avenue
New York, NY 10065
Tel: 212-327-7730 Fax: 212-327-8232
Email: kruegej@mail.rockefeller.edu

ITN Clinical Trial Physician

Dawn Smilek, MD, PhD
Associate Director, Clinical Trials Group
Immune Tolerance Network
185 Berry Street, Suite 3515
San Francisco, CA 94107
Tel: 415-353-4308 Fax: 415-353-4404
Email: dsmilek@immunetolerance.org

NIAID Medical Monitor

James McNamara, MD
Chief, Autoimmunity and Mucosal Immunology Branch
Division of Allergy, Immunology, and Transplantation / NIAID
5601 Fishers Lane, RM 7D31
Rockville, MD, 20892-9828
Tel: 240-627-3517
Email: jmcnamara@niaid.nih.gov

ITN Clinical Operations Manager

Karen Chia, CCRP
Manager, Clinical Operations
Clinical Trials Group
Immune Tolerance Network
185 Berry Street, Suite 3515
San Francisco, CA 94107
Tel: 415-353-4290 Fax: 415-353-4404
Email: kchia@immunetolerance.org

NIAID Project Manager

Susan Booher, RN, MS
Project Manager
Division of Allergy, Immunology, and Transplantation / NIAID
5601 Fishers Lane, RM 7D36
Rockville, MD, 20892-9828
Tel: 240-627-3481
Email: boohers@mail.nih.gov

ITN Lead Biologist

Kristina Harris, PhD
Director
Biomarker and Discovery Research
Immune Tolerance Network
7500 Old Georgetown Road, Suite 800
Bethesda, MD 20814
Tel: 240-235-6154 Fax: 240-235-6198
Email: kharris@immunetolerance.org

NIAID Regulatory Affairs

Theresa Allio, PhD
Regulatory Affairs Officer
Division of Allergy, Immunology, and Transplantation/NIAID
5601 Fishers Lane, Room 7B35
Rockville, Maryland 20852
Phone: 240-627-3476 Fax: 301-480-1537
E-mail: theresa.allio@nih.gov

Rho Scientist

James Rochon, PhD
Senior Statistical Scientist
Rho Inc.
6330 Quadrangle Drive
Chapel Hill, NC 27517
Tel: 919-595-6857 Fax: 919-408-0999
Email: james_rochon@rhoworld.com

This clinical study is supported and conducted by the Immune Tolerance Network, which is sponsored by the National Institute of Allergy and Infectious Diseases.

This document is confidential. It is provided for review only to investigators, potential investigators, consultants, study staff, and applicable independent ethics committees or institutional review boards. It is understood that the contents of this document will not be disclosed to others without written authorization from ITN and NIAID unless it is necessary to obtain informed consent from potential study participants.

Protocol Approval

Trial ID: ITN059AI	Protocol Version: 4.0
	Dated: February 26, 2015
IND # 117,633	**Protocol Chair:** James Krueger, MD, PhD
**Title:** Efficacy of Ustekinumab (Anti-IL-12/23) followed by Abatacept (CTLA4-Ig) for the Treatment of Psoriasis Vulgaris	

I confirm that I have read the above protocol in the latest version. I understand it, and I will work according to the principles of good clinical practice (GCP) as described in the US Code of Federal Regulations (CFR)—45 CFR part 46 and 21 CFR parts 50, 56, and 312, and in the International Conference on Harmonization (ICH) document *Guidance for Industry: E6 Good Clinical Practice: Consolidated Guidance* dated April 1996. Further, I will conduct the study in keeping with local legal and regulatory requirements.

As the principal investigator, I agree to carry out the study by the criteria written in the protocol and understand that no changes can be made to this protocol without written permission of the NIAID.

In signing, I also agree to the following:

i) To use and handle the study drugs only in accordance with the Protocol and for no other purpose;
ii) To not transfer the study drugs to any parties other than the NIH designated drug distributor;
iii) To not chemically modify, replicate, make derivatives of, or reverse engineer the study drugs.

____

Principal Investigator (*Print*)

____ ____________________

Principal Investigator (*Sign*) Date

Synopsis

**Title**	Efficacy of Ustekinumab (Anti-IL-12/23) followed by Abatacept (CTLA4-Ig) for the Treatment of Psoriasis Vulgaris
**Sponsor**	NIAID
**Conducted by**	Immune Tolerance Network
**Protocol Chair**	James G. Krueger, MD, PhD
**Study Treatment**	Sequential treatment with open label ustekinumab, followed by either continued ustekinumab and abatacept placebo, or abatacept and ustekinumab placebo.
**Study Design**	This trial will be conducted as a prospective, randomized, double-blind, multicenter, active comparator study in a maximum of 140 participants with psoriasis vulgaris to assess the efficacy of abatacept in perpetuating the known treatment benefit of ustekinumab following ustekinumab withdrawal. Efficacy of abatacept to induce prolonged disease remission following cessation of study medication will also be assessed.<br><br>The study design has a lead-in period of weight-based ustekinumab treatment, with all participants receiving either 45 mg ustekinumab (≤ 100 kg) or 90 mg ustekinumab (> 100 kg) administered subcutaneously at weeks 0 and 4. At week 12, participants will be assessed for a PASI 75 response to ustekinumab. Participants who do not achieve a PASI 75 score will be discontinued from the investigation and permitted to seek standard therapy.<br><br>The week 12 PASI 75 ustekinumab responders will be randomized 1:1 to receive either abatacept plus ustekinumab placebo or continued ustekinumab plus abatacept placebo. The trial design requires a sample size of 80 randomized participants. Published results indicate that 67.9% of participants who receive ustekinumab are expected to achieve a PASI 75 or greater response by week 12. Therefore, 120 participants are expected to be required in order to randomize the 80 required participants. The week 12 PASI 75 response rate will be monitored during the study. If it falls below the expected rate, a maximum of 20 additional participants will be enrolled to ensure that 80 participants are randomized at week 12. The initial enrollment will be set at 120 participants, with a maximum enrollment, if necessary, of 140 participants.<br><br>The abatacept treatment group will receive weekly subcutaneous injections of 125 mg abatacept from week 12 to week 39. The abatacept treatment group will also receive subcutaneous ustekinumab placebo at week 16 and week 28, corresponding to the ustekinumab dosing regimen.

The continued ustekinumab treatment group will receive subcutaneous injections of 45 mg ustekinumab ($\leq$ 100 kg) or 90 mg ustekinumab (> 100 kg) at week 16 and week 28. The ustekinumab treatment group will also receive weekly subcutaneous injections of abatacept placebo from week 12 to week 39, corresponding to the abatacept dosing regimen.

Study medication will be discontinued following the week 39 abatacept or abatacept placebo injection, and participants will be observed for disease relapse from week 40 through week 88. Following relapse, participants will be discontinued from observation, and will be permitted to seek standard therapy. There will be a follow-up safety visit 12 weeks after discontinuation.

In addition to clinical observation, 6 millimeter punch skin biopsies will be obtained from lesional skin at weeks 0, 12, 24, 40, 88, and at the time of psoriasis relapse, and from non-lesional skin at weeks 0, 40, 88, and at the time of psoriasis relapse.

Study Conduct

The study will be conducted at multiple sites in the United States and Canada. The study participation duration will be up to 100 weeks, which includes a treatment phase of 39 weeks, an observation phase of up to 49 weeks, and a follow-up visit 12 weeks after the observation phase is complete.

Primary Objective

The primary objective of the study is to determine whether co-stimulatory blockade with abatacept (CTLA4Ig) will induce tolerance and prevent relapses after medication has been discontinued in psoriasis vulgaris when administered following a decrease in the inflammatory response induced with ustekinumab (anti-IL-12/23).

Primary Endpoint

The primary endpoint is the proportion of participants who experience a psoriasis relapse at any time between week 12 and week 88. Psoriasis relapse is defined as loss of $\geq$ 50% of the initial PASI improvement measured at week 12. The primary endpoint will be assessed in all randomized participants.

Secondary Endpoints

Efficacy:

1. The proportion of randomized participants who experience a psoriasis disease relapse prior to week 40.
2. The proportion of participants who experience a psoriasis disease relapse between week 28 and week 88.
3. The proportion of participants who experience a psoriasis disease relapse between week 40 and week 88.
4. Mean length of time after week 12 to psoriasis relapse.
5. Physician's Global Assessment (PGA) of cleared or minimal at week 40 and week 88.

6. Dermatology Life Quality Index (DLQI) at week 40 and week 88.

Safety:

1. Frequency and severity of all AEs and SAEs.

Inclusion Criteria

Patients *must meet all* of the following criteria to be eligible for this study:

1. Males or females aged 18-65 years with a diagnosis of plaque psoriasis for at least 6 months.
2. Baseline PASI score ≥ 12.
3. ≥ 10% body surface area psoriasis involvement.
4. Willingness to forgo other available psoriasis therapies, live vaccines, and pregnancy during the trial.
5. Ability and willingness to provide informed consent and comply with study requirements.

Exclusion Criteria

Patients who *meet any* of the following criteria will *not* be eligible for this study:

1. Non-plaque forms of psoriasis.
2. Grade 2 or 3 moderate to severe psoriatic arthritis not adequately managed with non-steroidal anti-inflammatory drugs (NSAIDs).
3. Myocardial infarction, unstable angina, cerebrovascular accident, or other significant cardiovascular event within the previous one year.
4. Chronic obstructive pulmonary disease.
5. Comorbid condition that requires regular systemic corticosteroid treatment.
6. History of malignancy, except treated basal cell skin carcinoma.
7. Treated basal cell skin carcinoma within the previous 5 years.
8. Severe, progressive, or uncontrolled renal, hepatic, hematological, gastrointestinal, pulmonary, cardiac, or neurological disease, or any other medical condition that, in the investigator's opinion, places the participant at risk by participating in this study.
9. History of recent or ongoing uncontrolled bacterial, viral, fungal, or other opportunistic infections.
10. Evidence of infection with Hepatitis B Virus (HBV), Hepatitis C Virus (HCV), or Human Immunodeficiency Virus (HIV).
11. Positive QuantiFERON-TB Gold test. PPD tuberculin test may be substituted for QuantiFERON-TB Gold test.
12. Severe reaction or anaphylaxis to any human monoclonal antibody.
13. Any previous treatment with agents targeting IL-12 or IL-23, including ustekinumab.
14. Any previous treatment with abatacept.
15. Treatment with biologic agents within previous 3 months prior to visit 0, including adalimumab, etanercept, and infliximab.

16. Treatment with immunosuppressive medications, including methotrexate, cyclosporine, oral retinoids, prednisone, or phototherapy within previous 4 weeks prior to visit 0.
17. Topical psoriasis treatment within previous 2 weeks prior to visit 0, including topical corticosteroids, vitamin D analogues, retinoids, calcineurin inhibitors, salicylic acid, and coal tar.
18. Investigational study medication within previous 6 months prior to visit 0.
19. Liver function test (aspartate aminotransferase [AST], alanine aminotransferase [ALT], or alkaline phosphatase) results that are ≥ 2x the upper limit of normal (ULN).
20. Serum creatinine ≥ 2x the ULN.
21. Any of the following hematologic abnormalities, confirmed by repeat test at least 1 week apart:
    a. White blood count <3,000/µL or >14,000/µL;
    b. Lymphocyte count <1,000/µL;
    c. Neutrophil count <1,500/µL;
    d. Platelet count <150,000 /µL; *or*
    e. Hemoglobin <10 g/dL.
22. Females who are pregnant, lactating, planning on pregnancy during the study period, or unwilling to use a medically acceptable method of birth control.
23. Receipt of a live vaccine (e.g., varicella, measles, mumps, rubella, cold-attenuated intranasal influenza vaccine, and smallpox) in the previous 6 weeks prior to visit 0.
24. BCG vaccines in the previous one year.

Table of Contents

BACKGROUND AND RATIONALE....16
1.1 BACKGROUND....16
1.2 SCIENTIFIC RATIONALE....16
1.2.1 Immunological Basis of Psoriasis....16
1.2.2 Rationale for Combining Ustekinumab and Abatacept for Treatment of Psoriasis....16
1.2.3 Assessment of Mechanistic Correlates of Clinical Benefit in Skin....17
1.3 PRECLINICAL AND CLINICAL EXPERIENCE....18
1.3.1 Preclinical Studies....18
1.3.2 Clinical Studies....19
1.4 SUMMARY OF KNOWN AND POTENTIAL RISKS AND BENEFITS FOR HUMAN PARTICIPANTS....22
1.4.1 Risks....22
1.4.2 Benefits....24
2. OBJECTIVES....25
2.1 PRIMARY OBJECTIVE....25
2.2 SECONDARY OBJECTIVES....25
2.3 EXPLORATORY OBJECTIVES....25
3. STUDY DESIGN....25
3.1 DESCRIPTION....25
3.2 STUDY DURATION....28
3.3 STUDY ENDPOINTS....28
3.3.1 Primary Endpoint....28
3.3.2 Secondary Endpoints....28
3.3.3 Definitions....28
3.3.4 Study Population....29
3.4 RATIONALE FOR SELECTION OF DRUG, ROUTE, DOSE, AND REGIMEN....29
3.5 PREMATURE TERMINATION OR SUSPENSION OF THE TRIAL....29
3.5.1 Ongoing Review....29
3.5.2 Stopping Rules Guidance....30
4. ELIGIBILITY....30
4.1 INCLUSION CRITERIA....30
4.2 EXCLUSION CRITERIA....30

4.3 PREMATURE TERMINATION OF A PARTICIPANT FROM THE STUDY 31

5. STUDY MEDICATIONS 32

5.1 INVESTIGATIONAL MEDICATION: USTEKINUMAB 32

5.1.1 Formulation and Packaging 32

5.1.2 Placebo Preparations 32

5.1.3 Dosage and Administration 32

5.1.4 Recommended Storage Conditions 33

5.1.5 Toxicity Management for Ustekinumab 33

5.2 INVESTIGATIONAL MEDICATION: ABATACEPT 33

5.2.1 Formulation and Packaging 33

5.2.2 Abatacept Placebo 34

5.2.3 Dosage and Administration 34

5.2.4 Recommended Storage Conditions 34

5.2.5 Toxicity Management for Abatacept 34

5.3 DISCONTINUATION OF STUDY TREATMENT AND STUDY OBSERVATION 34

5.4 CONCOMITANT MEDICATIONS 35

5.4.1 Allowed Medications 35

5.4.2 Prohibited Medications 35

5.4.3 Contraception 36

5.5 DRUG ACCOUNTABILITY 36

5.6 ASSESSMENT OF COMPLIANCE WITH STUDY MEDICATION 36

6. STUDY PROCEDURES 36

6.1 VISIT WINDOWS 36

6.1.1 Scheduled Visits 36

6.1.2 Unscheduled Visits 37

6.1.3 Windows for Ustekinumab Injection 37

6.1.4 Windows for Abatacept Injection 37

6.1.5 Documentation of Psoriasis Relapse 37

6.1.6 Follow-up Visit 37

6.2 ENROLLMENT, RANDOMIZATION, BLINDING, AND UNBLINDING 37

6.2.1 Enrollment 37

6.2.2 Randomization 37

6.2.3 Blinding 38

6.2.4 Unblinding 38

6.3 GENERAL ASSESSMENTS 38
6.4 CLINICAL LABORATORY ASSESSMENTS 39
6.5 DISEASE-SPECIFIC ASSESSMENTS 39
6.6 MECHANISTIC ASSESSMENTS 40

7. MECHANISTIC ASSAYS 40

7.1 RATIONALE FOR IMMUNE STUDIES 40
7.2 PLANNED MECHANISTIC ASSAYS 41
7.2.1 Histological Assessments 41
7.2.2 Gene Expression in Peripheral Blood and Skin Biopsies 41
7.2.3 Multi-Parameter Flow Cytometry (MFC) 42
7.2.4 Serum Cytokine Assays 42
7.2.5 T-cell Repertoire Analysis 42
7.3 OVERVIEW OF DATA ANALYSIS 43
7.3.1 Individual Based Longitudinal Profiling 43
7.3.2 Exploratory Analysis 43
7.4 FUTURE / UNPLANNED STUDIES 43
7.5 SPECIMEN LOGISTICS 44
7.6 SPECIMEN TRACKING PROCEDURES 44
7.7 SPECIMEN STORAGE 44

8. ADVERSE EVENTS 45

8.1 OVERVIEW 45
8.2 PSORIASIS-SPECIFIC SAFETY DEFINITIONS 45
8.2.1 Worsening of Psoriasis 45
8.2.2 Psoriatic Arthritis 45
8.3 NON-DISEASE SPECIFIC SAFETY DEFINITIONS 46
8.3.1 Adverse Event 46
8.3.2 Suspected Adverse Reaction and Adverse Reaction 46
8.3.3 Serious Adverse Event 46
8.3.4 ‘Expected’ versus ‘Unexpected’ Suspected Adverse Reaction 47
8.4 COLLECTING AND RECORDING ADVERSE EVENTS 48
8.4.1 Methods of Collection 48
8.4.2 Collection Period for Adverse Events and Serious Adverse Events 48
8.4.3 Methods of Recording 48
8.5 GRADING AND ATTRIBUTION OF ADVERSE EVENTS 48

8.5.1 Grading 48
8.5.2 Attribution 49
8.6 REPORTING SERIOUS ADVERSE EVENTS 49
8.6.1 Reporting SAEs to the IND Sponsor 49
8.6.2 Reporting SAEs to Health Authorities 50
8.6.3 Reporting SAEs to the DSMB 51
8.6.4 Reporting Pregnancy 51

9. STATISTICAL CONSIDERATIONS AND ANALYTICAL PLAN 51
9.1 ANALYSIS SAMPLES 51
9.2 ANALYSIS OF ENDPOINTS 52
9.2.1 Drop-Outs 52
9.2.2 Primary Endpoint 52
9.2.3 Secondary Endpoints 52
9.2.4 Safety Analysis 54
9.2.5 Follow-up Visit Analysis 55
9.2.6 Medical History 55
9.2.7 Use of Medications 55
9.3 SAMPLE SIZE 55
9.4 REPORTING DEVIATIONS FROM THE ORIGINAL STATISTICAL PLAN 55

10. ACCESS TO SOURCE DATA/DOCUMENTS 56

11. QUALITY CONTROL AND QUALITY ASSURANCE 56

12. ETHICAL CONSIDERATIONS AND COMPLIANCE WITH GOOD CLINICAL PRACTICE 57
12.1 STATEMENT OF COMPLIANCE 57
12.2 INFORMED CONSENT 57
12.3 PRIVACY AND CONFIDENTIALITY 57

13. PUBLICATION POLICY 57

14. REFERENCES 58

APPENDIX 1. SCHEDULE OF EVENTS 63

List of Tables

Table 1 Attribution of Adverse Events……………………………………………………..49
Table 2 Primary/Secondary Endpoints and Analysis Samples………………………………..54

List of Figures

Figure 1 Loss of PASI Response after Ustekinumab Withdrawal……………………………20
Figure 2 Trial Design…………………………………………………………………….27

Abbreviations

ACR	American College of Rheumatology
ADL	Activities of Daily Living
AE	Adverse Event
ALT	Alanine Aminotransferase
ANCOVA	Analysis of Covariance Model
AR	Adverse Reaction
AST	Aspartate Aminotransferase
BCG	Bacillus Calmette-Guérin
BSA	Body Surface Area
CFR	Code of Federal Regulations
CRF	Case Report Form
COPD	Chronic Obstructive Pulmonary Disease
CTLA4	Cytotoxic T-Lymphocyte Antigen 4
DAIT	Division of Allergy, Immunology, and Transplantation
DC	Dendritic Cells
DLQI	Dermatology Life Quality Index
DMARD	Disease-Modifying Antirheumatic Drug
DSMB	Data and Safety Monitoring Board
EAE	Experimental Autoimmune Encephalomyelitis
EDC	Electronic Data Capture
ELISA	Enzyme-linked immunosorbent assay
FDA	Food and Drug Administration
GCP	Good Clinical Practice
HCG	Human Chorionic Gonadotropin
HBV	Hepatitis B Virus
HCV	Hepatitis C Virus

HIV	Human Immunodeficiency Virus
ICH	International Conference on Harmonization
IDO	Indoleamine-pyrrole 2,3-dioxygenase
IgG	Immunoglobulin G
IL	Interleukin
IND	Investigational New Drug
IRB	Institutional Review Board
ITN	Immune Tolerance Network
ITT	Intent to Treat
MACE	Major Adverse Cardiovascular Events
MedDRA	Medical Dictionary for Regulatory Activities
NCI-CTCAE	National Cancer Institute Common Terminology Criteria for Adverse Events
NCT	National Clinical Trial
NIAID	National Institute of Allergy and Infectious Diseases
OCT	Optimal Cutting Temperature
PASE	Psoriatic Arthritis Screening and Evaluation
PASI	Psoriasis Area and Severity Index
PBMC	Peripheral Blood Mononuclear Cell
PCR	Polymerase Chain Reaction
PGA	Physician’s Global Assessment
PP	Per Protocol
PPD	Purified Protein Derivative
PUVA	Psoralen Ultra-Violet A
RA	Rheumatoid Arthritis
RPLS	Reversible Posterior Leukoencephalopathy Syndrome
RPR	Rapid Plasma Reagin
SAE	Serious Adverse Event

SAP	Statistical Analysis Plan
SAR	Suspected Adverse Reaction
SAS	Statistical Analysis System
SDCC	Statistical and Data Coordinating Center
STS	Specimen Tracking System
SUSAR	Serious Unexpected Suspected Adverse Reaction
TCR	T cell receptor
Th	T helper
TNF	Tumor Necrosis Factor
ULN	Upper Limit of Normal
WHO	World Health Organization

Background and Rationale

1.1 BACKGROUND

Psoriasis is a chronic multisystem inflammatory disease predominantly involving the skin and joints, and affecting approximately 2% of the population. Plaque psoriasis is the most common form of psoriasis, affecting 80-90% of psoriasis patients [1]. Most of these patients (approximately 80%) have mild to moderate disease, which is generally treated with a variety of safe and effective topical agents.

A number of systemic therapies are available for moderate-severe psoriasis, defined as involvement of greater than 5-10% body surface area and/or significant interference with activities of daily life due to the regions affected (face, genitals, hands, and feet). These include phototherapy, methotrexate, cyclosporine, and acitretin. Though efficacious, each is associated with risks and contraindications, especially in the setting of comorbidities common among psoriasis patients. A number of US Food and Drug Administration (FDA) approved biologics are also available for first line therapy in moderate-severe psoriasis [1, 2]. These include the anti-tumor necrosis factor (TNF) agents infliximab (Remicade), etanercept (Enbrel), and adalimumab (Humira). In addition, ustekinumab (Stelara) is a monoclonal antibody which targets the inflammatory cytokines IL-12 and IL-23. Withdrawal of the biologic agents is associated with relapse of psoriasis, so ongoing administration is required. No treatment regimen has been identified which induces tolerance or remission.

1.2 SCIENTIFIC RATIONALE

1.2.1 Immunological Basis of Psoriasis

Psoriasis vulgaris is a T-cell mediated, inflammatory disease associated with skin lesions that contain activated Th1, Th17, and Th22 T-cell subsets. Human skin is an organ that contains a specialized immune system composed of differentiated, skin-homing memory T-cells and several types of dendritic antigen presenting cells. Overall, about 98% of memory T-cells differentiated for skin homing reside in the skin in adults, but in a resting state, suggesting they are largely tolerant to common cutaneous and environmental antigens. In psoriasis, there is expansion of cutaneous T-cells, and activation of both dendritic cells (DC) and T-cells, such that cytokines produced by activated T-cells and associated inflammatory pathways are increased. Because the skin is an accessible organ for biopsy, many biomarkers for T-cell activation and disease-related genes have been developed; ex vivo assays of T-cell and DC function are also possible for skin-resident cells.

1.2.2 Rationale for Combining Ustekinumab and Abatacept for Treatment of Psoriasis

One approved therapeutic approach for psoriasis vulgaris involves continuous use of the anti-cytokine antibody ustekinumab, which blocks IL-12 and IL-23 by binding to the common p40 subunit of these cytokines. The clinical response to ustekinumab has been well-documented in the PHOENIX 1, PHOENIX 2, and ACCEPT studies [3-5]. When ustekinumab was compared to placebo in moderate-to-severe psoriasis, most participants in PHOENIX 1 dramatically improved by week 12 [3]. However, when ustekinumab was discontinued at week 40, the improvement in psoriasis scores began

to decline by 44 weeks and the majority of participants relapsed by 64 weeks. Thus, while ustekinumab is highly effective in suppressing disease activity, discontinuation leads to predictable disease relapse, indicating that tolerance has not been achieved.

One approach to inducing longer-lasting T-cell tolerance is co-stimulatory blockade. Co-stimulatory blockade is based on the observation that T-cell activation requires two signals: 1) antigen-specific engagement of the T-cell receptor and 2) co-stimulatory B7/CD28 engagement [6-8]. Antigen-specific engagement of the T-cell receptor leads to T-cell tolerance when the co-stimulatory second signal is blocked. In theory, it might be possible to tolerize disease-mediating T-cells in psoriasis by a sequential approach that 1) decreases overall numbers and the activation state of T-cells and DCs in the skin by IL-12/23 blockade, thus lowering many co-stimulatory pathways that are up-regulated in active disease, and 2) exposes residual T-cells and DCs to B7 co-stimulatory blockade during the period where T-cells would be reactivated by endogenous antigen-presenting DCs.

The proposed drugs to test this tolerogenic strategy are 1) ustekinumab (anti-IL12/23), a monoclonal antibody approved by the FDA and Health Canada for psoriasis, and 2) abatacept (CTLA4-Ig), a co-stimulatory blocking fusion protein approved by the FDA and Health Canada for rheumatoid arthritis (RA) and juvenile rheumatoid arthritis. Abatacept is a fusion protein consisting of the extracellular domain of the CTLA4 ligand for B7 coupled to a modified Fc portion of human Immunoglobulin G (IgG). Although abatacept is not approved for the treatment of psoriasis by either the FDA or Health Canada, a treatment effect in psoriasis participants was demonstrated during early development of the drug [9]. In addition, psoriatic arthritis participants who received abatacept in a randomized controlled phase 2 trial showed improvement in the cutaneous manifestations of psoriasis [10]. In the current proposal, abatacept would be administered to participants only after activated DCs and T-cells have been suppressed by treatment with ustekinumab, increasing the chances for abatacept to induce tolerance in cutaneous T-cells that drive psoriasis.

1.2.3 Assessment of Mechanistic Correlates of Clinical Benefit in Skin

Psoriasis vulgaris is a chronic inflammatory skin disease characterized by increased epidermal thickening and T-cell infiltration. Treatment with abatacept has been associated with reduced cellular activation of T-cells, keratinocytes, DC, and vascular endothelium in the skin [11].

Mechanistic effects of ustekinumab on T-cells, DC, and skin pathology has also been measured, as a sub-study, in the ACCEPT trial that compared randomized treatment with ustekinumab vs. etanercept in moderate-to-severe psoriasis participants [5, 12-14]. Treatment of psoriasis vulgaris participants with ustekinumab for 12 weeks reduced T-cell and DC infiltration, and also reversed pathological epidermal hyperplasia so that treated psoriasis plaques resumed the appearance of non-lesional skin. Quantification of associated T-cells and DCs showed that both sets of cells are significantly reduced by ustekinumab treatment and that overall numbers are similar to levels measured in non-lesional skin at baseline. Cytokine production by DCs and T-cells was also reduced by ustekinumab treatment as determined by quantitative polymerase chain reaction (PCR) measures for mRNAs encoding IL-23, IL-17, IL-22

and interferon-gamma. Overall effects on gene transcription were measured, and more than 2900 disease-defining transcripts were reversed by ustekinumab. Finally, ustekinumab treatment led to a small reduction in circulating Th17 T-cells after 12 weeks of treatment, but similar effects were not produced by etanercept treatment [12-14].

In the proposed study, skin biopsies will be evaluated for cellular and molecular correlates of tolerance using immunohistochemistry for T-cell and DC markers, as well as mRNA microarrays and quantitative PCR to assess gene expression of cytokines and other immune regulatory molecules, according to a well-described approach [15, 16]. A successful outcome of tolerance induction at the tissue level in the proposed study would be defined as reduced production of IL-12/23 and T helper subset cytokines (gamma-interferon, IL-22, and IL-17A/F), along with a reduction of T-cells and DC. Other correlates of tolerance may be observed in skin biopsies, such as an increase of T-regs (assessed by Foxp3 phenotype), an increase of IDO+ regulatory DC, or increases in regulatory molecules such as IL-10, CTLA4, and PD-1. Thus, the trial presents the opportunity to define a molecular signature of tolerance which corresponds to a clinical response to sequential treatment with inflammatory cytokine blockade (ustekinumab) followed by co-stimulatory blockade (abatacept).

1.3 PRECLINICAL AND CLINICAL EXPERIENCE

1.3.1 Preclinical Studies

1.3.1.1 Preclinical studies with ustekinumab

Preclinical studies with administration of recombinant IL-12 demonstrated exacerbation of disease in a murine model of psoriasis [17], and in experimental autoimmune encephalomyelitis (EAE) [18], and monoclonal antibodies to the p40 subunit of IL-12 diminished or prevented disease in preclinical models of colitis, collagen-induced arthritis, and EAE [18-20]. These early studies suggested a role for IL-12 in the pathogenesis of psoriasis and other autoimmune diseases. It was subsequently discovered that IL-12 and IL-23 share the p40 subunit and that IL-23 is implicated in disease pathologies that were previously attributed to IL-12 [21-24].

An extensive preclinical toxicology program was undertaken in cynomolgus macaques during the development of ustekinumab [25]. Ustekinumab was shown to be pharmacologically active in this species, and potential immunomodulatory effects were explored. Due to specific concerns about exacerbation of Th2 mediated disease, ustekinumab was tested and shown not to exacerbate asthmatic responses in macaques. Also, the effect of ustekinumab on the cardiovascular system was examined and found not to be deleterious. In summary, ustekinumab was well tolerated in macaques over a broad therapeutic dosing window.

1.3.1.2 Preclinical studies with abatacept

CTLA4Ig has been tested in a variety of animal models. In early studies, investigators demonstrated that the effect of CTLA4Ig on the immune response to antigen is both dose and timing dependent. When administered within 2 days after antigen priming, CTLA4Ig induces both deletion and non-responsiveness of antigen-specific CD4+ T-

cells [26]. The blockade of B7-CD28 interaction during the secondary exposure of CD4+ T-cells to antigen appears to have a more profound effect on Th1 cells than on Th2 cells at low doses of antigen, but has no effect at high doses of antigen [27]. CD8+ T-cells are more resistant to co-stimulatory blockade than are CD4+ cells [28, 29]. B cells are highly sensitive to co-stimulatory blockade with CTLA4Ig. Both primary and secondary B-cell responses can be abrogated by high doses of CTLA4Ig [30, 31].

1.3.2 Clinical Studies

1.3.2.1 Clinical studies with ustekinumab

1.3.2.1.1 Clinical studies with ustekinumab in psoriasis

The safety and efficacy of ustekinumab in moderate-to-severe psoriasis vulgaris was demonstrated in two large randomized controlled trials [3, 4]. In the PHOENIX 1 trial, ustekinumab was compared to placebo in moderate-to-severe psoriasis, given at weeks 0, 4, and then every 12 weeks [3]. Greater than 60% of treated participants in PHOENIX 1 achieved the primary endpoint of a Psoriasis Area and Severity Index (PASI) 75 score by week 12. The PASI 75 score represents a 75% improvement in PASI [32, 33]. The treatment effect of ustekinumab was apparent by week 2, and maximum efficacy was achieved by week 24.

In the PHOENIX 1 study, ustekinumab was administered at weeks 0, 4, 16, and 28. Then at week 40, participants were randomized to continued ustekinumab or to ustekinumab withdrawal. In the withdrawal participants, the improvement in PASI scores began to decline by 44 weeks, with the decline accelerating after week 52 [3]. Figure 1 shows the cumulative percent of participants in the ustekinumab withdrawal group who experienced a psoriasis relapse in that they lost $\geq$ 50% of the initial week 12 PASI improvement [3]. In contrast, participants maintained on ustekinumab largely remained stable through week 76. Durable long term efficacy was maintained in the PHOENIX 1 trial for 3 years in participants who continued ustekinumab [34].

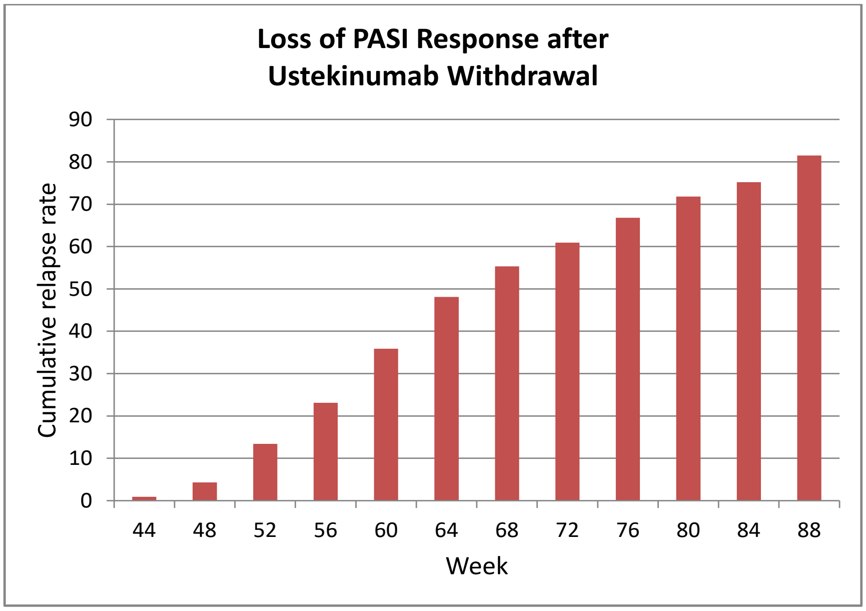

Figure 1 Loss of PASI Response after Ustekinumab Withdrawal

Similar results to those in PHOENIX 1 were observed in the PHOENIX 2 trial [4], which also explored partial response to ustekinumab. Independent predictors of partial response included higher body weight, inadequate response to a biologic agent, longer duration of psoriasis, and psoriatic arthritis. Partial response was associated with lower trough serum drug levels at week 28. However, accelerated dosing every 8 weeks rather than every 12 weeks did not result in improved efficacy in partial responders, except in the participants who received 90 mg of ustekinumab (vs. 45 mg).

A large randomized controlled trial in moderate-to-severe psoriasis (ACCEPT) compared efficacy and safety of ustekinumab vs. etanercept, an inhibitor of tumor necrosis factor [5]. ACCEPT demonstrated superior efficacy of ustekinumab over etanercept, especially at the 90 mg dose of ustekinumab. A response to ustekinumab was also observed in participants who did not respond to etanercept and subsequently crossed over to ustekinumab therapy, as well as those in whom treatment was interrupted following an initial response to ustekinumab.

1.3.2.1.2 Clinical studies with ustekinumab in psoriatic arthritis

Psoriatic arthritis is a manifestation present in approximately 10-25% of psoriasis patients [35, 36]. It is a chronic, inflammatory arthropathy often affecting multiple joints and is associated with substantial morbidity. The efficacy of ustekinumab was examined in a blinded, placebo-controlled, cross-over study in 140 participants with active psoriatic arthritis [37]. The primary endpoint of ACR20 (20% improvement in joint counts and ACR core criteria [38]) was met at week 12, with a significantly greater number of participants showing improvement in arthritis scores with ustekinumab vs. placebo. Skin lesion scores improved with ustekinumab as well, consistent with the results of the PHOENIX 1 and 2 trials. Adverse events (AE) were

similar in the ustekinumab and placebo groups. In a related report, ustekinumab was shown to also improve physical disability and health-related quality of life measures in participants with psoriatic arthritis [39].

1.3.2.1.3 Clinical studies with ustekinumab in Crohn's disease

Ustekinumab has been tested for efficacy and safety in moderate-to-severe Crohn's disease [40, 41]. In an early blinded placebo-controlled study of 104 participants, there was evidence of improved clinical response in participants who had previously been non-responders to the anti-TNF inhibitor infliximab [40].

A follow-up study was conducted in 526 moderate-to-severe refractory Crohn's disease participants whose disease was resistant to anti-TNF treatment [41]. Significantly, more participants met the primary endpoint and demonstrated a clinical response at week 6 to induction therapy with ustekinumab administered at week 0 (39.7% for 6 mg/kg ustekinumab versus 23.5% for placebo). Participants with a response were re-randomized at week 8 to maintenance therapy with 90 mg ustekinumab or placebo administered at weeks 8 and 16. As for the PHOENIX 1 trial in psoriasis, maintenance therapy with ustekinumab was required for a sustained clinical response. Adverse events were generally similar in the ustekinumab and placebo groups.

1.3.2.2 Clinical studies with abatacept

1.3.2.2.1 Clinical studies with abatacept in psoriasis and psoriatic arthritis

The safety and efficacy of abatacept was first shown in a phase 1 clinical trial in psoriasis. This trial was an open-label, dose escalation study in 41 participants with ≥ 10% body surface area involved, and used a dose range of 0.5 to 50 mg/kg [9]. Four doses were given over a 4 week period. Clinical responses were observed at doses of 4 to 8 mg/kg and above. Overall, 63% of study participants who received doses of at least 4 mg/kg achieved a 50% or greater improvement in clinical disease activity, compared with 4% of untreated participants. Abatacept was well-tolerated in this study.

Several mechanistic analyses accompanied this first abatacept trial. Clinical improvement was associated with reduced cellular activation of T-cells, keratinocytes, DC, and vascular endothelium in the skin, supporting the role of co-stimulation in maintaining the pathology of psoriasis [11]. Flow cytometry for B- and T-cell markers revealed no major alterations in peripheral blood lymphocytes, and serum Ig levels were unaffected.

More recently, a randomized controlled phase 2 trial of abatacept in 170 participants with psoriatic arthritis was conducted in which the cutaneous manifestations of psoriasis were assessed [10]. Participants received one of three abatacept dosing regimens or placebo at days 1, 15, and 29, followed by every 28 days thereafter. Compared to placebo, a significantly higher proportion of participants reached the ACR20 endpoint in the higher dose groups for abatacept, and the study met its primary endpoint. Cutaneous manifestations of psoriasis were assessed as secondary

endpoints, and improvement in the PASI scores were observed in all abatacept treatment groups. Safety profiles were equivalent in all treatment arms of the study.

1.3.2.2.2 Clinical studies with abatacept in rheumatoid arthritis

In 2005, abatacept was approved by the FDA for the treatment of adults with moderately to severely active rheumatoid arthritis who have had an inadequate response to one or more disease-modifying antirheumatic drugs (DMARDs). It was approved for use as monotherapy or in combination with DMARDs other than TNF antagonists. This approval came after a series of studies that consistently showed clinical benefit at doses of approximately 10 mg/kg every 4 weeks [42-45].

A phase 3 trial examined the response in participants with RA who had an inadequate response to methotrexate [44]. This study involved 652 participants who were randomized in a 2:1 ratio to receive abatacept 10 mg/kg or abatacept placebo every 4 weeks for 12 months. The ACR20, ACR50, and ACR70 response rates all showed statistically significant improvement for abatacept over placebo at the $p<0.001$ level.

Abatacept was also studied in RA participants who had an inadequate response to tumor necrosis factor (TNF) inhibition [45, 46]. In this study, 393 participants were randomized in a 2:1 ratio to receive abatacept 10 mg/kg or abatacept placebo superimposed on background methotrexate therapy. After six months of treatment, the ACR20 response rate was 50% in the experimental group and 20% in the control group ($p<0.001$); the ACR50 response in treated participants was 20% vs. 4% ($p<0.001$); and the ACR70 response in treated participants was 10% vs. 1.5% ($p<0.003$). The responses observed at six months were maintained over five years in a long term extension study [46]. Adverse events were comparable in both groups.

1.3.2.2.3 Clinical studies with abatacept in other disorders

Abatacept is FDA-approved for the treatment of juvenile idiopathic arthritis in children based on an initial 190 person randomized placebo-controlled trial [47]. A durable clinical response and long term safety were demonstrated in a follow-up long term extension study [48]. Abatacept also slows deterioration in beta cell function in Type 1 Diabetes during the initial six months of treatment, although the effect was not found to be durable over 2 years of treatment [49]. Abatacept was well tolerated but not efficacious in moderate-to-severe Crohn’s disease and ulcerative colitis [50].

Abatacept is currently being tested by the Immune Tolerance Network (ITN) for efficacy and safety in lupus nephritis and multiple sclerosis (MS) (ITN034AI, NCT#00774852 and ITN035AI, NCT#01116427). A trial in MS of a drug similar to abatacept was discontinued early and efficacy results were inconclusive [51].

1.4 SUMMARY OF KNOWN AND POTENTIAL RISKS AND BENEFITS FOR HUMAN PARTICIPANTS

1.4.1 Risks

1.4.1.1 Risks associated with ustekinumab

Ustekinumab is approved by the FDA and Health Canada for treatment of moderate-to-severe plaque psoriasis [52]. Serious infections and malignancies have been

reported with ustekinumab, but are not common. In patients with a genetic deficiency of IL-12/IL-23 there is a particular vulnerability to disseminated infections from mycobacteria and salmonella. Ustekinumab is an inhibitor of the cytokines IL-12 and IL-23 and poses a theoretical risk for vulnerability to these infections. A heightened awareness and early diagnostic evaluations for these infections is indicated. Anaphylaxis and anaphylactoid reactions have also been reported, but are not sufficiently common to support routine use of premedication with ustekinumab. Live vaccines should not be administered with ustekinumab, and Bacillus Calmette-Guérin (BCG) vaccines should not be administered during treatment with ustekinumab, or one year following discontinuation. One case of reversible posterior leukoencephalopathy syndrome (RPLS) was observed during the clinical development program. According to the package insert, the most common adverse events (AE) include nasopharyngitis, upper respiratory infection, headache, and fatigue [52].

An acceptable short term safety profile for ustekinumab was established in the PHOENIX 1, PHOENIX 2, and ACCEPT trials, in which adverse events (AE) and serious adverse events (SAE) were generally similar in the ustekinumab and placebo groups [3-5]. Longer term 3 year safety analyses have recently been reported for ustekinumab in psoriasis from pooled trial results, and showed that the safety profile of ustekinumab is generally maintained over time [53, 54]. Non-melanoma skin cancers have been reported with ustekinumab in psoriasis [53, 55], but rates were comparable to those reported in psoriasis with other biologics [56, 57] and were higher in participants who had been previously treated with PUVA (Psoralen Ultra-Violet A) light therapy. Serious cardiovascular AEs have been reported with ustekinumab [3-5], although a recent integrated safety analysis and a meta-analysis failed to demonstrate an increased risk of major adverse cardiovascular events (MACE) with ustekinumab compared to placebo [58, 59]. In contrast, an increased risk of MACE has been observed with briakinumab, another anti-IL-12,23 monoclonal antibody [60]. Therefore, participants with recent major cardiovascular events will be excluded from this study.

1.4.1.2 Risks associated with abatacept

Abatacept has been studied extensively in participants with RA, and is approved by the FDA and Health Canada for treatment of moderately to severely active RA in combination with DMARDs, but should not be given with TNF antagonists. It is also approved for severe juvenile idiopathic arthritis. The principal potential risk of treatment with abatacept is the suppression of T-cell-mediated immune responses that may increase the incidence of serious infections and malignancy, and both have been reported but are uncommon. Abatacept has been administered in clinical trials without premedication, but, despite this, the infusion tolerability is good. Anaphylaxis and anaphylactoid reactions have been reported. According to the package insert, common adverse events include headache, upper respiratory infection, nasopharyngitis, and nausea. Live vaccines should not be administered concurrently with abatacept, or within 3 months of discontinuation [61].

Abatacept is generally well tolerated with a good safety profile [62]. An integrated safety analysis of abatacept in combination with DMARDs included data from 2944

participants enrolled in five core abatacept trials, for 4764 patient-years of exposure [63]. Overall, rates of AE, SAE, and malignancies were similar in those receiving abatacept vs. placebo. The frequency of serious infections was low in both groups.

The ASSURE trial specifically examined the safety of abatacept in combination with biologic and nonbiologic DMARDs [64]. Participants were not excluded if they had stable comorbid conditions, such as asthma, chronic obstructive pulmonary disease (COPD), diabetes mellitus, and congestive heart failure. Again, rates of AEs and SAEs were similar in those receiving abatacept vs. placebo. Those receiving abatacept experienced more serious infections, all of which were bacterial in origin, than those receiving placebo (2.9% vs. 1.9%). There was evidence in this trial that participants with COPD may have an increased risk of AEs and SAEs involving the respiratory system, including infection, when treated with the combination of abatacept and DMARDs compared with receiving placebo and DMARDs. For this reason, participants with COPD have been excluded from this trial.

1.4.1.3 Risks of combination therapy with ustekinumab and abatacept

In this trial, ustekinumab and abatacept will be administered sequentially rather than combined at the same time. Ustekinumab will be administered at week 0 and week 4, with responding participants randomized to begin receiving abatacept at week 12, or to continue ustekinumab. Although the two study medications will not be administered in combination, there is potential for a limited period of systemically combined ustekinumab and abatacept, since the half-life of ustekinumab ranges from 14.9 to 45.6 days [52].

When two immunomodulatory biologics are administered in combination, there is a potential for synergy and increased risk of AEs and SAEs related to the immunosuppressive properties of the medications. In particular, there is a potentially greater risk of infection and malignancy. When abatacept was administered in combination with TNF antagonists over a one year time period in the ASSURE trial [64], AEs, SAEs, and infections were more frequent than with placebo in combination with the biologics.

However, the ARRIVE trial showed that participants with RA could be safely switched from TNF antagonists to abatacept without a washout period [46, 65]. Similarly, participants in the ACCEPT trial were safely switched from the TNF agent etanercept to ustekinumab without a washout period [5]. The results support the safety of sequential therapy with ustekinumab or abatacept following TNF antagonists, and suggest that systemic combination of these two biologics for a limited time period is not likely to present substantial risk to the participants.

1.4.2 Benefits

All participants in the study will benefit from receiving two doses of ustekinumab, an approved and highly efficacious treatment for psoriasis vulgaris (see section 3.1). Approximately 70% are expected to achieve a 75% or greater improvement in their psoriasis scores [3]. Of those participants who respond to ustekinumab, half will benefit from receiving two additional doses. The other half will receive abatacept, which has been shown in preliminary studies to be effective in psoriasis. Moreover,

participants in the abatacept group may experience a long-lasting disease remission, since abatacept has the potential to induce immunological tolerance.

2. OBJECTIVES

2.1 PRIMARY OBJECTIVE

The primary objective of the study is to determine whether co-stimulatory blockade with abatacept (CTLA4Ig) will induce tolerance and prevent relapses after medication has been discontinued in psoriasis vulgaris when administered following a decrease in the inflammatory response induced with ustekinumab (anti-IL-12/23).

2.2 SECONDARY OBJECTIVES

- Determine whether abatacept is as effective as ustekinumab in maintaining a clinical response induced by ustekinumab.
- Determine whether psoriasis relapses occur later following administration of abatacept compared to ustekinumab.
- Assess the effect of sequential treatment with ustekinumab followed by abatacept on general well-being and quality of life.
- Assess the frequency and severity of AEs and SAEs which occur during sequential treatment with ustekinumab followed by abatacept.

2.3 EXPLORATORY OBJECTIVES

- Assess the effect of sequential treatment with ustekinumab and abatacept on T-cell and dendritic cell subsets in the target organ (skin) and in peripheral blood.
- Assess the effect of sequential treatment with ustekinumab and abatacept on gene expression of cytokines and other immune regulatory molecules in the target organ (skin) and in peripheral blood.

3. STUDY DESIGN

3.1 DESCRIPTION

This trial will be conducted as a prospective, randomized, double-blind, multicenter, active comparator study in a maximum of 140 participants with psoriasis vulgaris to assess the efficacy of abatacept in perpetuating the known treatment benefit of ustekinumab following ustekinumab withdrawal. Efficacy of abatacept to induce prolonged disease remission following cessation of study medication will also be assessed.

The study design is depicted in Figure 2, and has a lead-in period of ustekinumab treatment, with all participants receiving either 45 mg ustekinumab ($\leq$ 100 kg) or 90 mg ustekinumab (> 100 kg) administered subcutaneously at weeks 0 and 4. At week 12, participants will be assessed for a PASI 75 response to ustekinumab (see section 3.3.3.1). Participants who do not achieve a PASI 75 score will be discontinued from the investigation and permitted to seek standard therapy.

The week 12 PASI 75 ustekinumab responders will be randomized 1:1 to receive either abatacept plus ustekinumab placebo or continued ustekinumab plus abatacept placebo. The trial design requires a sample size of 80 randomized participants (see section 9.3). Published results indicate that 67.9% of participants who receive ustekinumab are expected to achieve a PASI 75 or greater response by week 12 [3]. Therefore, 120 participants are expected to be required in order to randomize the 80 required participants. The week 12 PASI 75 response rate will be monitored during the study. If it falls below the expected rate, a maximum of 20 additional participants will be enrolled to ensure that 80 participants are randomized at week 12. The initial enrollment will be set at 120 participants, with a maximum enrollment, if necessary, of 140 participants.

The abatacept treatment group will receive weekly subcutaneous injections of 125 mg abatacept from week 12 to week 39. The abatacept treatment group will also receive subcutaneous ustekinumab placebo at week 16 and week 28, corresponding to the ustekinumab dosing regimen.

The continued ustekinumab treatment group will receive subcutaneous injections of 45 mg ustekinumab ($\leq$ 100 kg) or 90 mg ustekinumab (> 100 kg) at week 16 and week 28. The ustekinumab treatment group will also receive weekly subcutaneous injections of abatacept placebo from week 12 to week 39, corresponding to the abatacept dosing regimen.

Study medication will be discontinued following the week 39 abatacept or abatacept placebo injection, and participants will be observed for disease relapse from week 40 through week 88 (see section 3.3.3.2). Following relapse, participants will be discontinued from observation, and will be permitted to seek standard therapy. There will be a follow-up safety visit 12 weeks after discontinuation.

In addition to clinical observation, 6 millimeter punch skin biopsies will be obtained from lesional skin at weeks 0, 12, 24, 40, 88, and at the time of psoriasis relapse, and from non-lesional skin at weeks 0, 40, 88, and at the time of psoriasis relapse.

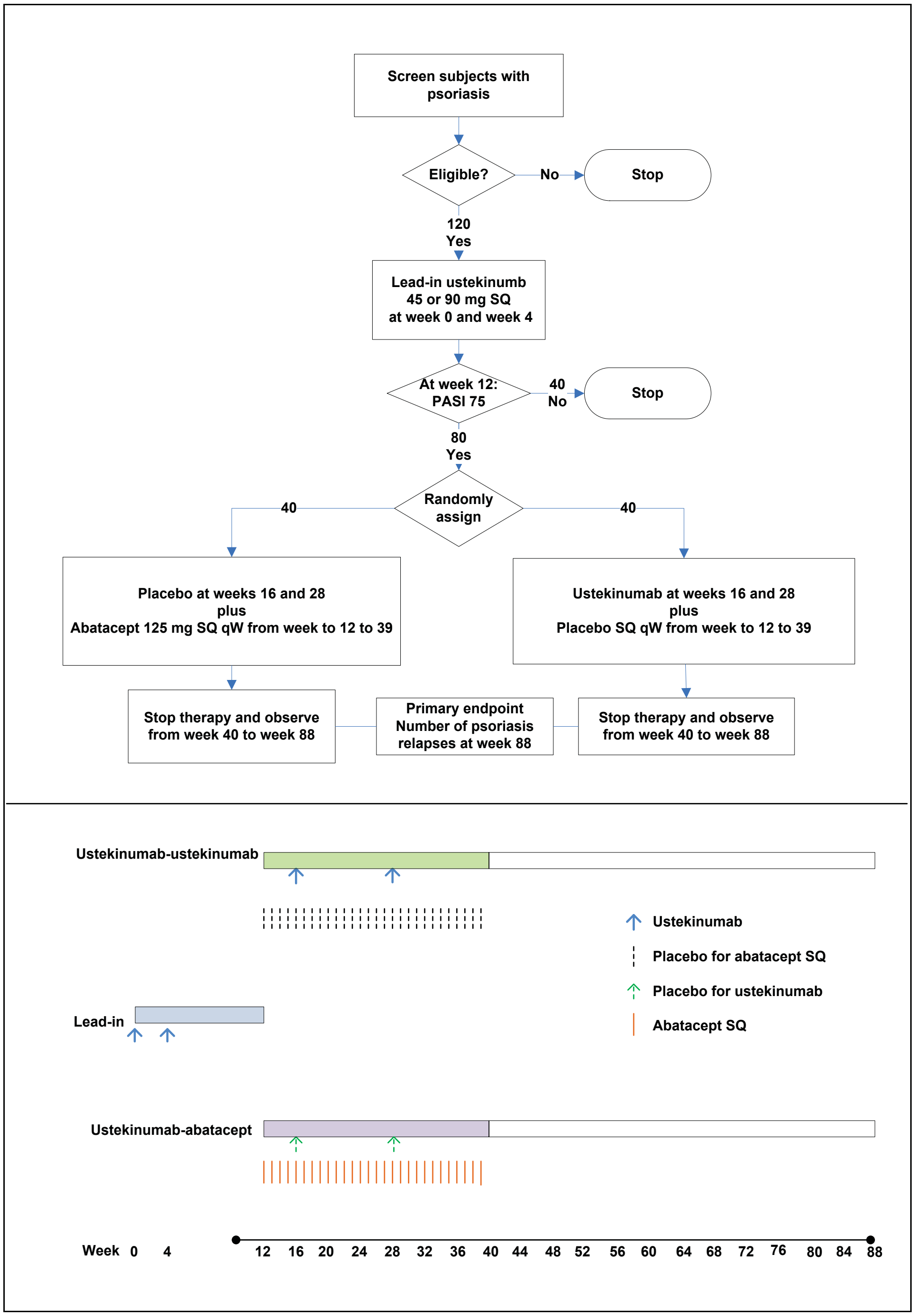

Figure 2 Trial Design

3.2 STUDY DURATION

Total study duration will be 204 weeks:

- Enrollment phase will be 104 weeks.
- Study participation phase will be up to 100 weeks, which includes a treatment phase of 39 weeks, an observation phase of up to 49 weeks, and a follow-up visit 12 weeks after the observation phase is complete.

3.3 STUDY ENDPOINTS

3.3.1 Primary Endpoint

The primary endpoint is the proportion of participants who experience a psoriasis relapse at any time between week 12 and week 88. Psoriasis relapse is defined as loss of $\geq$ 50% of the initial PASI improvement measured at week 12 (see Section 3.3.3.2). The primary endpoint will be assessed in all randomized participants.

3.3.2 Secondary Endpoints

Efficacy:

1. The proportion of randomized participants who experience a psoriasis disease relapse prior to week 40 (see Section 3.3.3.2).
2. The proportion of randomized participants who experience a psoriasis disease relapse between week 28 and week 88 (see Section 3.3.3.2).
3. The proportion of participants who experience a psoriasis disease relapse between week 40 and week 88 (see Section 3.3.3.2).
4. Mean length of time after week 12 to psoriasis relapse.
5. Physician's Global Assessment (PGA) [3] of cleared or minimal at week 40 and week 88.
6. Dermatology Life Quality Index (DLQI) [66, 67] at week 40 and week 88.

Safety:

1. Frequency and severity of all AEs and SAEs.

3.3.3 Definitions

3.3.3.1 Psoriasis response

A psoriasis response measured at week 12 is defined as greater than or equal to a 75% improvement in the PASI, according to the following formula:

Response PASI $\leq$ 0.25 (Baseline PASI)

3.3.3.2 Psoriasis relapse

A psoriasis relapse is defined as loss of $\geq$ 50% of the initial PASI improvement measured at week 12, according to the following formula:

Relapse PASI = Week 12 PASI + [(Baseline PASI –Week 12 PASI)/2]

3.3.4 Study Population

Male and female participants age 18-65 with moderate-to-severe plaque psoriasis will be recruited at multiple sites in the United States and Canada. Participants will be enrolled without regard to gender or race.

3.4 RATIONALE FOR SELECTION OF DRUG, ROUTE, DOSE, AND REGIMEN

Our trial design is built on the framework of the successful phase 3 PHOENIX 1 and PHOENIX 2 trials for ustekinumab in psoriasis [3, 4]. The proposed study is a prospective randomized controlled trial for individuals with moderate-to-severe psoriasis vulgaris. Open label ustekinumab will be administered subcutaneously according to the standard dosing regimen used in the PHOENIX 1 and PHOENIX 2 trials and described in the ustekinumab prescribing information [52].

Abatacept is approved for treatment of RA and juvenile idiopathic arthritis, and has an excellent safety record. Although abatacept is not approved by either the FDA or Health Canada for the treatment of psoriasis, a treatment effect in psoriasis participants was demonstrated during early development of the drug [9]. In addition, psoriatic arthritis participants who received abatacept in a randomized controlled phase 2 trial showed improvement in the cutaneous manifestations of psoriasis [10]. Abatacept will be administered according to the current prescribing information for adult rheumatoid arthritis with weekly doses of subcutaneous abatacept which are not adjusted for weight [61]. Subcutaneous abatacept has been shown to be effective without an intravenous loading dose [68, 69].

Abatacept will be administered starting at week 12 in participants who achieve a PASI 75 or greater response to ustekinumab and are randomized to abatacept. Based on the PHOENIX 1 and 2 trials, approximately 70% of participants are expected to achieve a response by week 12, with relatively little gain of additional responders after this time [3, 4].

3.5 PREMATURE TERMINATION OR SUSPENSION OF THE TRIAL

3.5.1 Ongoing Review

The progress of the study will be monitored by the National Institute of Allergy and Infectious Diseases (NIAID) Autoimmune Data and Safety Monitoring Board (DSMB), which will review safety data and make recommendations regarding continuation, termination, or modification of the study. Based on a 104 week enrollment period and an additional study participation period of 100 weeks, the DSMB will formally review the safety data approximately 6 months after study enrollment begins. Following this initial review, the DSMB will subsequently review the safety data at least yearly. The number of subjects who discontinue study treatment will also be included in the reports prepared for the DSMB.

In addition, safety data will be reviewed by the DSMB when an event occurs that is of sufficient concern to the NIAID medical monitor, ITN physician or protocol chair to

warrant review, or when an event occurs that contributes to a stopping rule listed in section 3.5.2.

3.5.2 Stopping Rules Guidance

If any of the following events occur, the DSMB chair will be notified and a review of safety data will be performed to determine if enrollment in the study should be stopped and/or administration of investigational study medication should be halted:

1. Any death that is at least possibly related to use of the investigational study medication.
2. Two or more of the same Preferred Term grade 3 or greater clinical AEs, or two or more of the same cardiovascular System Organ Class grade 3 or greater AEs, involving different participants that are at least possibly related to use of the investigational study medication.

4. ELIGIBILITY

4.1 INCLUSION CRITERIA

Participants must meet *all* of the following criteria to be eligible for this study:

1. Males or females aged 18-65 years with a diagnosis of plaque psoriasis for at least 6 months.
2. Baseline PASI score ≥ 12.
3. ≥ 10% body surface area psoriasis involvement.
4. Willingness to forgo other available psoriasis therapies, live vaccines, and pregnancy during the trial.
5. Ability and willingness to provide informed consent and comply with study requirements.

4.2 EXCLUSION CRITERIA

Participants who meet any of the following criteria will *not* be eligible for this study:

1. Non-plaque forms of psoriasis.
2. Grade 2 or 3 moderate to severe psoriatic arthritis not adequately managed with non-steroidal anti-inflammatory drugs (NSAIDs). (see section 8.2.2)
3. Myocardial infarction, unstable angina, cerebrovascular accident, or other significant cardiovascular event within the previous one year.
4. Chronic obstructive pulmonary disease.
5. Comorbid condition that requires regular systemic corticosteroid treatment.
6. History of malignancy, except treated basal cell skin carcinoma.
7. Treated basal cell skin carcinoma within the previous 5 years.
8. Severe, progressive, or uncontrolled renal, hepatic, hematological, gastrointestinal, pulmonary, cardiac, or neurological disease, or any other medical condition that, in the investigator's opinion, places the participant at risk by participating in this study.
9. History of recent or ongoing uncontrolled bacterial, viral, fungal, or other opportunistic infections.

10. Evidence of infection with Hepatitis B Virus (HBV), Hepatitis C Virus (HCV), or Human Immunodeficiency Virus (HIV).
11. Positive QuantiFERON-TB Gold test. PPD tuberculin test may be substituted for QuantiFERON-TB Gold test.
12. Severe reaction or anaphylaxis to any human monoclonal antibody.
13. Any previous treatment with agents targeting IL-12 or IL-23, including ustekinumab.
14. Any previous treatment with abatacept.
15. Treatment with biologic agents within previous 3 months prior to visit 0, including adalimumab, etanercept, and infliximab.
16. Treatment with immunosuppressive medications, including methotrexate, cyclosporine, oral retinoids, prednisone, or phototherapy within previous 4 weeks prior to visit 0.
17. Topical psoriasis treatment within previous 2 weeks prior to visit 0, including topical corticosteroids, vitamin D analogues, retinoids, calcineurin inhibitors, salicylic acid, and coal tar.
18. Investigational study medication within previous 6 months prior to visit 0.
19. Liver function test (aspartate aminotransferase [AST], alanine aminotransferase [ALT], or alkaline phosphatase) results that are ≥ 2x the upper limit of normal (ULN).
20. Serum creatinine ≥ 2x the ULN.
21. Any of the following hematologic abnormalities, confirmed by repeat test at least 1 week apart:
    a. White blood count <3,000/µL or >14,000/µL;
    b. Lymphocyte count <1,000/µL;
    c. Neutrophil count <1,500/µL;
    d. Platelet count <150,000 /µL; *or*
    e. Hemoglobin <10 g/dL.
22. Females who are pregnant, lactating, planning on pregnancy during the study period, or unwilling to use a medically acceptable method of birth control.
23. Receipt of a live vaccine (e.g., varicella, measles, mumps, rubella, cold-attenuated intranasal influenza vaccine, and smallpox) in the previous 6 weeks prior to visit 0.
24. BCG vaccines in the previous one year.

4.3 PREMATURE TERMINATION OF A PARTICIPANT FROM THE STUDY

Withdrawal of consent. Participants who withdraw consent for further treatment will be asked if they would be willing to complete an end-of-study visit to include the assessments in visit 22.

Investigator decision. The investigator may choose to withdraw a participant from the study for any reason.

Failure to return. Participants who do not return for visits and who do not respond to repeated attempts by the site staff to have them return will be considered *lost to follow-up*.

Participants who prematurely terminate from the study will not be replaced.

5. Study Medications

5.1 INVESTIGATIONAL MEDICATION: USTEKINUMAB

5.1.1 Formulation and Packaging

Ustekinumab is supplied as a sterile solution in the following forms:

- a single-use 2 ml Type 1 glass vial of 45 mg in 0.5 ml.
- a single-use pre-filled syringe with a 27 gauge fixed passive needle guard and a needle cover that is manufactured using a dry natural rubber (a derivative of latex) with either 45 mg in 0.5 ml or 90 mg in 1.0 ml.

Each 45 mg ustekinumab pre-filled syringe or single-use vial also contains: L-histidine and L-histidine monohydrochloride monohydrate (0.5 mg), Polysorbate 80 (0.02 mg), and sucrose (38 mg) to fill to a final volume of 0.5 mL. Each 90 mg ustekinumab pre-filled syringe also contains: L-histidine and L-histidine monohydrochloride monohydrate (1 mg), Polysorbate 80 (0.04 mg), and sucrose (76 mg) to fill to a final volume of 1 mL. Ustekinumab solution is colorless to slightly yellow in appearance and has a pH of 5.7-6.3, and does not contain preservatives.

Procurement, packaging and delivery of ustekinumab pre-filled syringes and single-use vials will be done by a designated drug distributor under contract to NIAID. The syringes and single-use vials provided to the pharmacy will have study-specific investigational agent labels.

5.1.2 Placebo Preparations

Ustekinumab placebo syringes, which consist of sterile normal saline, will be prepared by the site pharmacist.

5.1.3 Dosage and Administration

Dosage and administration schedule for ustekinumab and ustekinumab placebo is described in section 3.1. Ustekinumab will be administered as a subcutaneous injection on week 0 and week 4. Ustekinumab or ustekinumab placebo will be administered as a subcutaneous injection on week 16 and week 28.

Participants who weigh $\leq$100 kg at study entry will receive 45 mg of ustekinumab or 0.5 ml ustekinumab placebo. Participants who weigh $>$ 100 kg at study entry will receive 90 mg of ustekinumab or 1.0 ml ustekinumab placebo.

Ustekinumab or ustekinumab placebo should be administered according to the instructions provided in the approved package labeling.

US package insert [52]:
http://www.stelarainfo.com/hcp/assets/pdf/PrescribingInformation.pdf

Canada product monograph: http://www.janssen.ca/product/190

5.1.4 Recommended Storage Conditions

Ustekinumab pre-filled syringes and single-use vials must be refrigerated at 2ºC to 8ºC (36ºF to 46ºF). Store ustekinumab pre-filled syringes and single-use vials upright. Keep the product in the original carton to protect from light until the time of use. Do not freeze. Do not shake. Ustekinumab does not contain a preservative; discard any unused portion.

When ustekinumab single-use vials are used, ustekinumab syringes will be prepared by the site pharmacist on the day of injection. Ustekinumab placebo syringes will also be prepared by the site pharmacist on the day of injection.

5.1.5 Toxicity Management for Ustekinumab

Suspend ustekinumab administration if the participant develops an infection or other AE that the investigator judges to be significant. The scheduled dose of ustekinumab at visit 1 (week 4) may be administered within the specified visit window if the infection or AE resolves within this time frame (see section 6.1.1). The scheduled dose of ustekinumab at visit 4 (week 16) and visit 7 (week 28) may be administered within 10 days outside of the visit window if the infection or AE resolves within this time frame.

Discontinue ustekinumab if any of the following occurs:

- A grade 3 or greater AE that the protocol chair and/or the medical monitor, in consultation with the site investigator, judge to be possibly, probably, or definitely related to ustekinumab.
- One dose of ustekinumab is withheld.

If ustekinumab is discontinued, the procedures in Section 5.3 should be followed.

5.2 INVESTIGATIONAL MEDICATION: ABATACEPT

5.2.1 Formulation and Packaging

Abatacept solution for subcutaneous administration is supplied as a sterile, preservative-free, clear, colorless to pale yellow solution with a pH of 6.8 to 7.4. Each single dose of subcutaneous injection provides 125 mg abatacept, dibasic sodium phosphate anhydrous (0.838 mg), monobasic sodium phosphate monohydrate (0.286 mg), poloxamer 188 (8 mg), sucrose (170 mg), and quantity sufficient to 1 mL with water for injection.

Abatacept injection solution for subcutaneous administration is supplied as a single-dose disposable pre-filled glass syringe with flange extender. The Type I glass syringe has a coated stopper and fixed stainless steel needle (5 bevel, 29-gauge thin wall, 1/2-inch needle) covered with a rigid needle shield. The pre-filled syringe provides 125 mg of abatacept in 1 mL. The syringes provided to the pharmacy will have study-specific investigational agent labels.

Abatacept pre-filled syringes will be provided by Bristol Myers Squibb. Packaging and delivery of abatacept pre-filled syringes will be done by a designated drug distributor under contract to NIAID.

5.2.2 Abatacept Placebo

Abatacept placebo compositionally matches abatacept minus the active ingredient, and will be provided by Bristol Myers Squibb, supplied as pre-filled syringes. The pre-filled syringes provided to the pharmacy will have study-specific investigational agent labels.

Packaging and delivery of abatacept placebo pre-filled syringes will be done by a designated drug distributor under contract to NIAID.

5.2.3 Dosage and Administration

As described in section 3.1, abatacept or abatacept placebo will be administered weekly by subcutaneous injection at weeks 12 through 39 at a dose of 125 mg via single use syringe according to the instructions provided in the package insert [61] http://packageinserts.bms.com/pi/pi_orencia.pdf.

5.2.4 Recommended Storage Conditions

Abatacept or abatacept placebo solution supplied in pre-filled syringes should be refrigerated at 2°C to 8°C (36°F to 46°F). Do not use beyond the expiration date on the pre-filled syringe. Protect from light by storing in the original package until time of use. Do not allow the pre-filled syringe to freeze.

5.2.5 Toxicity Management for Abatacept

Suspend abatacept or abatacept placebo administration if the participant develops an infection or other AE that the investigator judges to be significant. If the infection or AE resolves, abatacept or abatacept placebo may be restarted at the next scheduled dose.

Discontinue abatacept or abatacept placebo if any of the following occurs:

- A grade 3 or greater AE that the protocol chair and/or the medical monitor, in consultation with the site investigator, judge to be possibly, probably, or definitely related to abatacept.
- Three sequential doses of abatacept are withheld.
- More than four total doses of abatacept are withheld.

If abatacept is discontinued, the procedures in section 5.3 should be followed.

5.3 DISCONTINUATION OF STUDY TREATMENT AND STUDY OBSERVATION

Study treatment is defined as the dosing and administration of study medication according to study specification. Study observation is defined as study participation after week 39 until the time of discontinuation, or through week 88 if discontinuation

does not occur earlier. Study treatment or study observation will be discontinued for an individual participant if *any* of the following criteria are met:

- The participant meets the criterion for psoriasis relapse (see section 3.3.3.2).
- Discontinuation criteria for ustekinumab are met prior to week 28, or discontinuation criteria for abatacept are met between week 12 and week 39 (see section 5.1.5 and 5.2.5).
- Onset of generalized pustular psoriasis.
- Onset of erythrodermic psoriasis.
- Onset of grade 2 or 3 moderate to severe psoriatic arthritis not adequately managed with NSAIDs (see section 8.2.2) or with intra-articular corticosteroid injection as allowed in section 5.4.1.
- The investigator determines that it is in the participant's best interest to discontinue treatment.
- The participant requests that treatment or observation be halted.
- The participant becomes pregnant.

Further care will be provided according to the judgment and practice of the principal investigator.

At this time the participant will complete all assessments listed for visit 22 in Appendix 1. Randomized participants will be also be asked to return for a follow-up visit 12 weeks later, and will complete the assessments listed for visit D. This will conclude his or her participation in the study.

If study treatment or study observation is discontinued, the NIAID medical monitor should be notified unless the participant discontinues study treatment or observation due to psoriasis relapse, as described in the study design (see section 3.1).

5.4 CONCOMITANT MEDICATIONS

5.4.1 Allowed Medications

Use of the following medications is allowed during study participation:

- One course of oral or inhaled corticosteroids not to exceed 2 weeks may be used at the investigator's discretion for the treatment of non-psoriasis conditions.
- An intra-articular injection of corticosteroids not to exceed one injection every six months may be used at the investigator's discretion for treatment of joint pain.

5.4.2 Prohibited Medications

Use of the following medications is prohibited during study participation:

- All medications listed in the Exclusion Criteria (section 4.2), except as described in section 5.4.1.

- Live vaccines concurrently or within 3 months of study medication discontinuation.
- BCG vaccines concurrently or within one year following study medication discontinuation.

5.4.3 Contraception

All female participants of childbearing age must use a medically acceptable form of contraception during study participation, and must continue its use for 8 weeks after receiving their last dose of study medication.

5.5 DRUG ACCOUNTABILITY

Under US and Canadian regulations (21CFR 312.62 and C.05.012) an investigator is required to maintain adequate records of the disposition of the investigational product, including the date and quantity of drug that was received, the participants to whom drug was dispensed (participant by participant accounting), and an account of any drug accidentally or deliberately destroyed. The investigator will ensure that the investigational product supplies are stored as specified in the protocol and pharmacy manual in a secured area, with access limited to authorized study personnel as described in the clinical study agreement.

Records for receipt, storage, use, and disposition of the study drug will be maintained by the study sites. A drug-dispensing log will be kept current for each participant and will contain the identification of each participant, and the date and quantity of drug dispensed. All remaining unused investigational product will be returned to the sponsor or sponsor's representative after study termination, or destroyed with the permission of the sponsor in accordance with applicable law and study site procedures. If investigational product is to be destroyed locally, the investigator will provide documentation in accordance with sponsor's specifications.

All records regarding disposition of the investigational product will be available for inspection by the clinical trial monitor.

5.6 ASSESSMENT OF COMPLIANCE WITH STUDY MEDICATION

Ustekinumab and ustekinumab placebo will be administered by trained medical staff. Compliance, therefore, will be monitored by medical staff and documented on the case report form (CRF). Abatacept and abatacept placebo will be self-administered by the participant; compliance will be monitored by return of unused syringes to study coordinators and documented on the CRF.

6. STUDY PROCEDURES

6.1 VISIT WINDOWS

6.1.1 Scheduled Visits

Appendix 1 presents the schedule of events for this trial. Visit 0 must occur within 28 days of Visit –1. All other scheduled study visits must occur within the time limits specified below:

Visits 1 through 22: ±5 days

6.1.2 Unscheduled Visits

Unscheduled visits may be performed to document any symptoms or to document a psoriasis relapse during the study. Assessments for an unscheduled visit are listed in Appendix 1. Some of the assessments may be omitted at the discretion of the investigator if they are not indicated.

6.1.3 Windows for Ustekinumab Injection

The schedule for ustekinumab or ustekinumab placebo injection is described in Appendix 1. Ustekinumab or ustekinumab placebo injections must occur with the scheduled visit windows described in section 6.1.1, except as specified in the special circumstances described in section 5.1.5.

6.1.4 Windows for Abatacept Injection

The schedule for abatacept or abatacept placebo injection is described in Appendix 1. Abatacept or abatacept placebo injections must occur within 72 hours of the scheduled injection.

6.1.5 Documentation of Psoriasis Relapse

Psoriasis relapse may be documented at a scheduled or an unscheduled visit. Psoriasis relapse is a criterion for discontinuation of study treatment and study observation (see section 5.3). All assessments listed at visit 22 should be completed.

6.1.6 Follow-up Visit

Randomized participants will be asked to return for a follow-up visit D 12 weeks following discontinuation from the study, as described in section 5.3, or 12 weeks after completing the study at week 88. All assessments listed at visit D should be completed. The visit window for visit D is specified below.

Visit D: ±7 days

6.2 ENROLLMENT, RANDOMIZATION, BLINDING, AND UNBLINDING

6.2.1 Enrollment

Participants who meet the eligibility criteria will be enrolled at study visit 0.

6.2.2 Randomization

Participants who meet the response criteria of PASI 75 (see section 3.3.3.1) will be randomly assigned to treatment in either the ustekinumab group or the abatacept group. Randomization will be accomplished through a password-protected, web-based, randomization system (RhoRAND™) maintained by the Statistical and Data Coordinating Center (SDCC).

Random assignment will be stratified according to PASI score 12-20 versus PASI score > 20 at visit 0. Stratification based on the PASI score at visit 0 was chosen

because the study is evaluating a sequential strategy consisting of a lead-in phase with open label ustekinumab, followed by either abatacept or continued ustekinumab.

6.2.3 Blinding

Blinding will be maintained for all study participants and trial personnel from the time of randomization at week 12 throughout the study, with the exception of the site pharmacist. In the event that pre-filled ustekinumab syringes rather than single-use vials are supplied for the week 16 and week 28 blinded ustekinumab doses, one unblinded trained medical professional at each site who will administer ustekinumab or ustekinumab placebo at week 16 and week 28 will also be required. This unblinded individual will not otherwise be involved in any other aspect of the study. The week 16 and week 28 doses of ustekinumab and ustekinumab placebo will be delivered from the pharmacy and administered in masked fashion in order to preserve blinding of participants and all other site personnel.

6.2.4 Unblinding

Unblinding before the study is completed will occur only if a participant's well-being is threatened and the investigator believes unblinding is necessary to protect the participant.

Before treatment assignment for an individual participant is unblinded, the investigator must confer with the NIAID medical monitor. The investigator will notify the protocol chair of the unblinding event, and the medical monitor will notify the study management team.

The emergency unblinding will be recorded and reported to the DSMB. A full account of the event will be recorded, including the date and time of the emergency, the reason for the decision to unblind, and the names of the medical monitor and others who were notified of the emergency. During site visits, the site monitor must verify that the medical monitor was notified and that a written account was completed. The reasons for unblinding of a participant's treatment will be included in the final study report.

Immune Tolerance Network (ITN) and NIAID approval is required for unblinding the treatment of an individual participant or subgroups of participants for unplanned interim analyses to support DSMB reviews and final analysis.

An exception to the above rule is that Investigational New Drug (IND) Safety Reports will be reported to the FDA, Health Canada, DSMB, and Institutional Review Boards (IRB) or ethics review committee in an unblinded fashion as requested by current ICH and local guidance.

6.3 GENERAL ASSESSMENTS

- Informed consent. Written informed consent will be obtained before any study assessments or procedures are performed.
- Randomization.
- Demographics: age and ethnicity.

- Medical history: to determine if the there are any clinically significant diseases or medical procedures other than the disease under study.
- Psoriasis history to determine time of diagnosis.
- Comprehensive physical examination.
- Brief physical examination: a physical examination focused on participant's current complaints and clinical status at the study visit will be conducted.
- Adverse events: participants will be assessed for AEs.
- Concomitant medications: all concomitant medications will be recorded.
- Vital signs: weight, temperature, blood pressure, respiration, and pulse will be obtained at all visits.

6.4 CLINICAL LABORATORY ASSESSMENTS

- Serum Chemistry (AST, ALT, bilirubin, alkaline phosphatase, random glucose)
- Hematology (CBC, differential and platelet count)
- Serum Creatinine
- Rapid Plasma Reagin (RPR)
- Serum Human Chorionic Gonadotropin (HCG)
- QuantiFERON-TB Gold, unless test has been performed within 30 days of visit -1 and documented test results are available. (PPD tuberculin skin test may be substituted for the QuantiFERON-TB Gold.)
- Urinalysis
- STAT Urine Human Chorionic Gonadotropin (HCG)
- HIV, unless test has been performed within 30 days of visit -1 and documented test results are available.
- Hepatitis B (surface antibody, core antibody, and surface antigen), unless test has been performed within 30 days of visit -1 and documented test results are available.
- Hepatitis C (RNA or antibody), unless test has been performed within 30 days of visit -1 and documented test results are available.
- Chest x-ray, unless x-ray has been performed within 30 days of visit -1 and documented test results are available.

6.5 DISEASE-SPECIFIC ASSESSMENTS

- Psoriasis Area and Severity Index (PASI) [32, 33]
- Physician's Global Assessment (PGA) [3]
- Dermatology Life Quality Index (DLQI) [66, 67]

http://www.dermatology.org.uk/quality/dlqi/quality-dlqi-questionnaire.html

- Psoriatic Arthritis Screening and Evaluation (PASE) [70, 71]
- Skin photography (optional)

6.6 MECHANISTIC ASSESSMENTS

- Lesional and non-lesional six millimeter punch skin biopsy. Two four millimeter biopsies can be substituted for each six millimeter biopsy. The week 24 lesional skin biopsy and the relapse non-lesional biopsy are considered optional. In participants who do not experience a psoriasis relapse, the week 88 non-lesional biopsy will be considered optional.
- Plasma/Serum assays
- PBMC assays
- RNA assays

See section 7 for detailed discussion of mechanistic assays.

7. MECHANISTIC ASSAYS

7.1 RATIONALE FOR IMMUNE STUDIES

Psoriasis vulgaris is a chronic inflammatory skin disease characterized by increased epidermal thickening and T-cell infiltration. Clinical response to ustekinumab in psoriasis vulgaris has been associated with the restoration on skin biopsy of near normal levels of T-cells and DC, elimination of most activated DC, suppression of T-cell cytokine levels, and reversal of pathological epidermal hyperplasia so that treated psoriasis plaques resumed the appearance of non-lesional skin [5]. Treatment with abatacept has also been associated with reduced cellular activation of T-cells, keratinocytes, DCs, and vascular endothelium in the skin [11].

In this study, skin and blood samples will be collected with the objective of analyzing circulating cytokine levels, cellular infiltration, and genetic signatures of disease and tolerance. It is hypothesized that tolerance can be induced by 1) decreasing T-cell and DC numbers and activation (ustekinumab) and 2) prevention of T-cell reactivation (abatacept). The primary mechanistic objective for the study will be the evaluation of cellular and molecular correlates of tolerance in skin. The skin biopsies will be evaluated using immunohistochemistry for T-cell and DC markers, as well as mRNA microarrays and quantitative PCR to assess gene expression of cytokines and other immune regulatory molecules, according to a well-described approach [15, 16].

A successful outcome of tolerance induction at the tissue level in the proposed study would be defined as reduced production of IL-12/23 and T helper subset cytokines (gamma-interferon, IL-22, and IL-17A/F), along with a reduction of T-cells and DC. Other correlates of tolerance may be observed in skin biopsies, such as an increase of T-regs (assessed by Foxp3 phenotype), an increase of IDO+ regulatory DC, or increases in regulatory molecules such as IL-10, CTLA4, and PD-1. Thus, the trial

presents the opportunity to define a molecular signature of tolerance which corresponds to a clinical response to sequential treatment with inflammatory cytokine blockade (ustekinumab) followed by co-stimulatory blockade (abatacept).

7.2 PLANNED MECHANISTIC ASSAYS

Samples will be collected from psoriasis participants at weeks 0, 12, 24, 40, 56, 72, 88, and at the time of psoriasis relapse. Six millimeter skin biopsies will be collected from lesional and non-lesional (unaffected) skin and cut into two halves, one half for RNA isolation and one half for immunohistochemical analysis. Alternatively, two four millimeter biopsies can be substituted for each six millimeter biopsy. Blood will be collected in ABI Tempus™ blood collection tubes for gene expression analysis and in heparinized tubes for PBMC isolation and plasma characterization. Samples will be shipped for processing, storage, and/or analysis to the Core Laboratories as specified in the General Information description of the ITN Tolerance Assay Core Facilities. The laboratory at Rockefeller University has extensive experience processing skin biopsies for immunohistochemical analysis and gene expression analysis [15, 16, 72, 73].

7.2.1 Histological Assessments

The laboratory at Rockefeller University has worked out conditions for the evaluation of infiltrating T-cell subsets and DC in skin biopsies [72, 74, 75]. Therefore, lesional and non-lesional (unaffected) skin biopsies will be submerged in OCT compound upon collection and shipped to Rockefeller for processing and analysis. Skin sections will be stained with antibodies directed against T-cell, dendritic cell, and cytokines. Cellular infiltration will be compared between treatment groups to evaluate the efficacy of treatment on reducing T-cell subsets and DC in lesional skin. Lesional skin biopsies will additionally be compared to non-lesional skin biopsies from the same participant.

7.2.2 Gene Expression in Peripheral Blood and Skin Biopsies

Systemic treatment with biologics has previously been shown to modulate gene expression in psoriasis vulgaris participants [76]. The accessibility of skin provides the opportunity to evaluate the direct effect of treatment on the target organ. Whole blood will additionally be collected in ABI Tempus™ blood collection tubes to evaluate changes in the periphery due to immunomodulation of the disease or the systemic nature of the treatment. Skin biopsies and whole blood will be collected from enrolled participants to evaluate genomic changes following treatment with ustekinumab or the combination of ustekinumab followed by abatacept. RNA will be isolated, and gene expression of selected cytokines and other immune regulatory molecules will be further evaluated using quantitative PCR as previously described [77].

Biopsy tissue will be compared pre- and post-treatment to determine the effect of treatment on the global gene expression in participants with psoriasis. Since drug effects may confound mechanistic observations, care needs to be taken in the interpretation of data comparing participants treated with ustekinumab compared to ustekinumab followed by abatacept. Therefore, comparisons at baseline will be

important to demonstrate that all participants have similar inflammatory signatures prior to treatment.

7.2.3 Multi-Parameter Flow Cytometry (MFC)

Flow cytometry will be done at ITN laboratories to analyze the peripheral blood immune-cell-phenotype and identify specific cells or cellular subsets that correlate with tolerance or other clinical phenotype. We hypothesize that the combination of ustekinumab followed by abatacept will decrease plasmacytoid dendritic cells and Th17 cells, while up-regulating regulatory T-cell populations.

Peripheral blood will be collected and shipped to the ITN core laboratory for flow cytometric analysis. Frozen flow cytometry experiments will use banked specimens to investigate longitudinal changes in T-cells, monocytes, and dendritic cells. Circulating T-cells, DC, monocytes, and other inflammatory cells will be characterized by staining cells with antibodies directed against immune markers, such as CD19, CD3, CD4, CD8, CD25, CD45RA, CD45RO, CD11b/Mac-1, CD11c, CD16, CCR6, CTLA-4, IDO, PD-1, CLA, HLA-DR, BDCA1, BDCA2, CD80, CD86, CD28, IL-22, IFN-g, IL-17, IL-13, Foxp3, and CD127. Proposed frozen flow antibody panels will be finalized and validated by the ITN flow core prior to starting assays. Levels of circulating cells will be compared between the baseline and time points following treatment. Additional comparisons will be made between treatment groups to evaluate effectiveness of ustekinumab or ustekinumab followed by abatacept on circulating cells.

7.2.4 Serum Cytokine Assays

Participant serum/plasma will be analyzed for circulating levels of cytokines, such as IL-1β, IL-2, IL-4, IL-5, IL-6, IL-7, IL-8, IL-10, IL-12p70, IL-13, IL-17, G-CSF, GM-CSF, IFN-γ, MCP-1, MIP1β, and TNF-α, using the Bio-Plex Pro Human Cytokine 17-plex assay (Bio-Rad) or equivalent. Additionally, circulating levels of additional inflammatory mediators, such as MIP-3α/CCL20, IL-22, IL-12/23p40, and IL-23p19 will be evaluated by enzyme-linked immunosorbent assay (ELISA).

Serum cytokine levels for individual cytokines and inflammatory mediators will be compared between baseline and the time points following treatment. Additionally, levels will be evaluated for correlations with circulating T-cell and monocyte populations. Additional comparisons will be made between treatment groups to evaluate effectiveness of ustekinumab or ustekinumab followed by abatacept on circulating levels of inflammatory mediators.

7.2.5 T-cell Repertoire Analysis

Psoriasis is a chronic inflammatory skin disease characterized by epidermal hypoproliferation and dermal infiltration leading to the formation of raised scaly plaques or “lesions.” The dermal infiltrate of psoriatic lesions is predominantly characterized by increased numbers of plasmacytoid dendritic cells and T-cells (both CD4+ and CD8+ T-cells). Current biological therapies (i.e. adalimumab, etanercept) targeting the inflammatory process provide a clinical benefit and disease resolution, however, relapse and lesion materialization occurs in the absence of continued therapy due, in part, to a remnant disease signature in the skin [77]. The proposed treatment

paradigm of ustekinumab followed by abatacept is hypothesized to disrupt this interaction by inhibiting the inflammatory response of the pDC and preventing T-cell activation by blocking co-stimulation, thereby leading to disease resolution and remission. Using T-cell receptor (TCR) repertoire sequencing, we will evaluate whether therapeutic treatment and the subsequent immunological disruption modulates the antigen specificity of infiltrating and resident T-cells in psoriatic skin. In order to characterize the repertoire of autoreactive T-cells and determine whether disease relapse involves the same T-cells, TCR sequencing will be performed on skin biopsies collected from enrolled patients at baseline, following therapeutic treatment, and during disease relapse in those that fail to maintain remission.

7.3 OVERVIEW OF DATA ANALYSIS

This study is designed to assess the ability of combination therapy to induce sustained disease resolution as evidenced clinically by a decreased PASI score and sustained remission. Mechanistic data collected as secondary endpoints will be analyzed as both cohort and individual participant-based longitudinal profiling which includes graphic plotting and descriptive statistics. Exploratory analysis is also planned to discover potential molecular and cellular patterns that might be linked to disease resolution.

7.3.1 Individual Based Longitudinal Profiling

Mechanistic samples from participants enrolled in this study will be assayed as described in the individual sections above with the aim to identify specific cell phenotypes, intracellular responses and /or gene sets (tissue and/or peripheral blood) that correlate with successful disease resolution. These data may also be assessed in support of evaluating the safety of the treatment regimen. Graphic plots will facilitate longitudinal monitoring of relevant cellular populations, immune cell repertoire, and gene expression etc., in conjunction with the treatment process, adverse events and clinical outcomes. Descriptive statistics will be provided as appropriate.

7.3.2 Exploratory Analysis

The study participants may be grouped based on primary clinical outcome (disease resolution success and failure). The primary objective of exploratory analysis is to discover molecular, cellular, and immunogenetic patterns among the participants at clinical milestones during the study that may provide insights into potential association with the suppression of the inflammatory process in skin and disease resolution. Pattern recognition methods including hierarchical clustering and principal component analysis may be used for the analysis. The exploratory analysis will be conducted on individual assay datasets (flow cytometry, gene expression, histological assessment assays, etc.) as well as integrated multi-assay data.

7.4 FUTURE / UNPLANNED STUDIES

Specimens stored during the trial may be used in future assays to reevaluate biological responses as research tests are developed over time. Additionally, samples may be used for assays/experiments outside the scope of this proposal, such as investigation of differences in the T-cell receptor (TCR) repertoire as evaluated by sequencing, proteomics or other assays that may emerge and be compelling. Re-evaluations or new assays will only be performed on samples of participants who have consented for

future research. The ITN sample sharing policy will apply for the provision of samples to study or outside investigators (www.immunetolerance.org).

7.5 SPECIMEN LOGISTICS

The clinical sites will be trained in collection, processing, shipment, and tracking of mechanistic research specimens. The ITN will monitor specimen quality, shipping compliance etc. and retrain the clinical site if not producing optimum quality mechanistic samples. The Rockefeller University clinical site will process mechanistic samples for gene expression and histological analysis according to the ITN standard operating procedures and use the ITN Specimen Tracking System (STS) software to identify and track all mechanistic specimens. The site is required to have its own laboratory equipment for use in standard ITN procedures, such as a centrifuge for spinning primary blood tubes, a micropipettor to aliquot specimens and freezer to store frozen specimens until they can be shipped. Additional mechanistic samples will be shipped to ITN repositories/ core labs, per ITN standard procedures. All shipping will conform to country-specific regulations for Diagnostic Specimens.

7.6 SPECIMEN TRACKING PROCEDURES

The ITN will track all mechanistic specimens until the final disposition of all material is known. Samples will remain in the ITN repository until used for assays or destroyed.

7.7 SPECIMEN STORAGE

Samples sent to the ITN repository will be stored under specific conditions to maintain long-term sample integrity, as well as specimen tracking from receipt to shipment to alternate locations. The ITN STS will be used to track date of shipment, location shipped to, carrier, items shipped, amount shipped, barcode numbers, protocol number, and associated comments about each individual specimen. Storage temperature, location, processing, aliquoting, and freeze/thaw events may also be recorded.

If the study subject allows storage, the subject's specimens will be stored indefinitely. The subject can change their mind at any time and have their stored specimens destroyed by notifying the study physician in writing. In such cases, the site coordinator would send all requests for sample destruction to the ITN. The site will receive confirmation that the specimen was destroyed as requested. If the subject's samples have already been analyzed, then the data will be used as part of the overall analysis. The subject can only request to have samples destroyed if they still exist, i.e. have not already been used in an experiment.

Specimens at the ITN core or repository can only be transferred to another destination with appropriate authorization per ITN standard procedures. The following items are checked prior to authorization for the purpose of accessing/transferring the specimen (within study assay as defined by the protocol or future studies): 1) evaluation of subject consent for the purpose provided 2) verification of specimen identifiers, and 3) quality and quantity of the specimen.

8. ADVERSE EVENTS

8.1 OVERVIEW

The investigator is responsible for the detection and documentation of events meeting the criteria and definition of an AE or SAE as described in sections 8.2 and 8.3 in this protocol. All AEs and SAEs will be recorded in the source documents and on the appropriate electronic CRF(s). All data will be reviewed periodically by the DSMB, which may provide recommendations to NIAID about withdrawing any participant and/or terminating the study because of safety concerns.

Adverse events that are classified as serious according to the definition of health authorities must be reported promptly and appropriately to the NIAID, ITN, principal investigators in the trial, IRBs or ethics review committees, and health authorities as appropriate. This section defines the types of AEs and outlines the procedures for appropriately collecting, grading, recording and reporting them. Information in this section complies with 21CFR 312; Division 5 of the Canadian Food and Drug Regulations; International Conference on Harmonization (ICH) Guideline E2A*: Clinical Safety Data Management: Definitions and Standards for Expedited Reporting*; and ICH Guideline E-6: *Guidelines for Good Clinical Practice*; and applies the standards set forth in the National Cancer Institute (NCI) *Common Terminology Criteria for Adverse Events Version 4.03* (published June 14, 2010). (This document is referred to herein as the "NCI-CTCAE manual.")

8.2 PSORIASIS-SPECIFIC SAFETY DEFINITIONS

8.2.1 Worsening of Psoriasis

Psoriasis severity will be assessed by PASI and recorded on a designated electronic CRF. Worsening psoriasis will not be collected as an AE. Psoriasis relapse is defined in section 3.3.3.2, and will be a criteria for discontinuation of study medication as described in section 5.3.

8.2.2 Psoriatic Arthritis

New onset or worsening psoriatic arthritis will be graded according to the following functional scale:

Grade 1: Mild pain with inflammation, erythema, or joint swelling.

Grade 2: Moderate pain associated with signs of inflammation, erythema, or joint swelling; limiting instrumental activities of daily living (ADL).

Grade 3: Severe pain associated with signs of inflammation, erythema, or joint swelling; irreversible joint damage; disabling; limiting self-care ADL.

8.3 NON-DISEASE SPECIFIC SAFETY DEFINITIONS

8.3.1 Adverse Event

An AE is any occurrence or worsening of an undesirable or unintended sign, symptom, laboratory finding, or disease that occurs during participation in the trial. An AE will be followed until it resolves or until 30 days after a participant terminates from the study, whichever comes first. All AEs will be recorded as specified in section 8.4.3 whether they are or are not related to disease progression or study participation.

8.3.2 Suspected Adverse Reaction and Adverse Reaction

Suspected adverse reaction (SAR) means any adverse event for which there is a reasonable possibility that the study drug caused the adverse event. For the purposes of safety reporting, “reasonable possibility” means there is evidence to suggest a causal relationship between the drug and the adverse reaction. A suspected adverse reaction implies a lesser degree of certainty about causality than adverse reaction, which means any adverse event caused by a drug (21 CFR 312.32(a) and ICH E2A).

An adverse reaction (AR) means any adverse event caused by a study drug. Adverse reactions are a subset of all suspected adverse reaction for which there is reason to conclude that the drug caused the event.

8.3.3 Serious Adverse Event

An AE or SAR is considered “serious” if, in the view of either the investigator or Division of Allergy, Immunology, and Transplantation (DAIT)/NIAID it results in any of the following outcomes (21 CFR 312.32(a), Division 5 of the Canadian Food and Drug Regulations, and ICH E2A):

- Death: A death that occurs during the study or that comes to the attention of the investigator during the protocol-defined follow-up period must be reported whether it is considered treatment related or not.
- A life-threatening event: An AE or SAR is considered “life-threatening” if, in the view of either the investigator or DAIT/NIAID, its occurrence places the subject at immediate risk of death. It does not include an AE or SAR that, had it occurred in a more severe form, might have caused death.
- Inpatient hospitalization or prolongation of existing hospitalization.
- Persistent or significant incapacity or substantial disruption of the ability to conduct normal life functions.
- An event that requires intervention to prevent permanent impairment or damage. An important medical event that may not result in death, be life threatening, or require hospitalization may be considered an SAE when, based on appropriate medical judgment, it may jeopardize the participant and may require medical or surgical intervention to prevent one of the outcomes listed above.
- Congenital anomaly or birth defect.

If an event meets any of the above serious adverse event definitions, regardless of the relationship of the event to study drug, the event must be reported to the sponsor as described in section 8.6.1.

8.3.3.1 Events considered study specific SAEs

- Potential drug induced liver injury (DILI) defined as:
  - ALT or AST elevation > 3 times ULN.

    AND
  - Total bilirubin > 2 times ULN, without initial findings of cholestasis (elevated serum alkaline phosphatase),

    AND
  - No other immediately apparent possible causes of ALT or AST elevation and hyperbilirubinemia, including, but not limited to, viral hepatitis, pre-existing chronic or acute liver disease, or the administration of other drug(s) known to be hepatotoxic.
- Suspected transmission of an infectious agent (e.g. any organism, virus or infectious particle, pathogenic or non-pathogenic) via the study drug.

If an event meets any of the above criteria, regardless of the relationship of the event to study drug, the event must be reported to the sponsor as described in section 8.6.1.

8.3.3.2 Non-serious study specific events tracked as SAEs

- Any cancer.
- Overdose.

If an event meets any of the above criteria, regardless of the relationship of the event to study drug, the event must be reported to the sponsor as described in section 8.6.1. If such an event does not meet serious criteria as described in Section 8.3.3, it will nonetheless be reported on the SAE form as a non-serious event and entered into the safety database for tracking purposes.

8.3.4 'Expected' versus 'Unexpected' Suspected Adverse Reaction

A SAR is considered "expected" when it is listed in the investigator brochure, the package insert or the protocol. A SAR is considered "unexpected" when its nature (specificity), severity, or rate of occurrence is not consistent with applicable product information as described in the safety information provided in the investigator brochure, the package insert, the Canada product monograph, or the protocol (21 CFR 312.32(a), Division 5 of the Canadian Food and Drug Regulations, and ICH E2A). A serious unexpected suspected adverse reaction is referred to as a SUSAR. For this study, expectedness will be determined by product information provided in the package insert for ustekinumab and abatacept [52, 61].

8.4 COLLECTING AND RECORDING ADVERSE EVENTS

8.4.1 Methods of Collection

Adverse events may be collected as follows:

- Observing the participant.
- Questioning the participant in an objective manner.
- Receiving an unsolicited complaint from the participant.

An abnormal value or result from a clinical or laboratory evaluation (e.g., a radiograph, an ultrasound, or an electrocardiogram) can also indicate an AE if it is a Grade 2 or greater AE per CTCAE criteria. The evaluation that produced the value or result should be repeated until that value or result returns to normal or can be explained and the participant's safety is not at risk.

8.4.2 Collection Period for Adverse Events and Serious Adverse Events

All AEs and SAEs will be collected from visit -1 until 30 days after the participant completes the study (visit 22) or prematurely withdraws from the study. AEs and SAEs will also be collected at visit D.

8.4.3 Methods of Recording

8.4.3.1 Recording AEs

Throughout the study, the investigator will record all clinical AEs and all laboratory AEs ≥ grade 2 on the appropriate eCRF regardless of their relation to study participation. The investigator will treat participants experiencing AEs appropriately and observe them at suitable intervals until their symptoms resolve or their status stabilizes.

8.4.3.2 Recording SAEs

Serious AEs will be recorded on the SAE eCRF and health authorities will be notified as outlined in section 8.6.2.

8.5 GRADING AND ATTRIBUTION OF ADVERSE EVENTS

8.5.1 Grading

The study site will grade the severity of AEs experienced by study participants according to the criteria set forth in the NCI-CTCAE manual (v 4.03 published June 14, 2010). This manual provides a common language to describe levels of severity, to analyze and interpret data, and to articulate the clinical significance of all AEs.

Adverse events will be graded on a scale from 1 to 5 according to the following standards in the NCI-CTCAE manual:

- Grade 1 = mild adverse event.
- Grade 2 = moderate adverse event.
- Grade 3 = severe and undesirable adverse event.

- Grade 4 = life-threatening or disabling adverse event.
- Grade 5 = death.

For additional information and a printable version of the NCI-CTCAE manual, go to http://ctep.cancer.gov/reporting/ctc.html

8.5.2 Attribution

Adverse events will be categorized for their relation to the following study medications:

- ustekinumab
- abatacept

The principal investigator will make the initial determination of the relation, or attribution, of an AE to study drug and will record the initial determination on the appropriate eCRF and/or SAE reporting form. The relation of an AE to study drug will be determined using definitions in the table below. Final determination of attribution for safety reporting will be decided by DAIT/NIAID.

Table 1 Attribution of Adverse Events

Code	Descriptor	Definition
Unrelated Category		
1	Unrelated	The adverse event is clearly not related.
2	Unlikely	The adverse event is unlikely related.
Related Categories		
3	Possible	The adverse event has a reasonable possibility of being related; there is evidence to suggest a causal relationship.
4	Probable	The adverse event is likely related.
5	Definite	The adverse event is clearly related.

8.6 REPORTING SERIOUS ADVERSE EVENTS

8.6.1 Reporting SAEs to the IND Sponsor

The following process for reporting an SAE ensures compliance with 21CFR 312, Division 5 of the Canadian Food and Drug Regulations, and ICH guidelines. After learning that a participant has experienced an SAE, the principal investigator or designee will report the SAE via the electronic SAE report form (SAE eCRF) within 24 hours of becoming aware of the event. The initial SAE eCRF should include as much information as possible, but at a minimum must include the following:

- AE term
- Study drug treatment
- Relationship to study medications
- Reason why the event is serious

- Supplementary CRF pages must be current at the time of SAE reporting: medical history, concomitant medications, demographics, study drug administration, death.

As additional details become available, the SAE eCRF should be updated and submitted. Every time the SAE eCRF is submitted, it should be electronically signed by the investigator.

For additional information regarding SAE reporting, contact Rho Product Safety:

Rho Product Safety
6330 Quadrangle Drive, Suite 500
Chapel Hill, NC 27517
Toll-free - (888) 746-7231
SAE Fax Line: 1-888-746-3293
Email: rho_productsafety@rhoworld.com

8.6.2 Reporting SAEs to Health Authorities

After the SAE has been reported by the principal investigator and assessed by the sponsor of the application with regulatory authorities, the sponsor of the application with regulatory authorities must report the event to the appropriate health authorities using one of these 2 options:

- **Standard reporting (IND annual report).** This option applies if the AE is classified as one of the following:
  - Serious, expected, suspected adverse reaction described in section 8.3.2, 8.3.3, and section 8.3.4.
  - Serious and not a suspected adverse reaction described in section 8.3.2 and 8.3.3.
- **Expedited reporting (Expedited safety report).** This option applies if the AE is classified as one of the following:
  1. Serious and unexpected suspected adverse reaction (SUSAR) described in section 8.3.3, and unexpected per sections 8.3.2, 8.3.3, and 8.3.4.

     The sponsor must report any suspected adverse reaction that is both serious and unexpected. The sponsor must report an adverse event as a suspected adverse reaction only if there is evidence to suggest a causal relationship between the study drug and the adverse event, such as:

     - A single occurrence of an event that is uncommon and known to be strongly associated with drug exposure (e.g., angioedema, hepatic injury, or Stevens-Johnson Syndrome);
     - One or more occurrences of an event that is not commonly associated with drug exposure, but is otherwise uncommon in the population exposed to the drug (e.g., tendon rupture);

- An aggregate analysis of specific serious adverse events observed in a clinical trial (such as known consequences of the underlying disease or condition under investigation or other events that commonly occur in the study population independent of drug therapy) that indicates those events occur more frequently in the drug treatment group than in a concurrent or historical control group.

2. Any findings from clinical or epidemiological studies, analysis of data pooled across multiple studies, published or unpublished scientific papers, or from animal or in vitro testing that would result in a safety-related change in the protocol, informed consent, investigator brochure, or other aspects of the overall conduct of the trial will be reported.

Expedited safety reports must be reported by DAIT/NIAID to the appropriate health authorities within 15 calendar days; fatal or immediately life-threatening, serious, unexpected, suspected adverse reactions must be reported within 7 calendar days.

All principal investigators must report SAEs to their respective IRBs or ethics review committees as mandated by them.

Final Study Report: A complete summary of safety information (including both Standard and Expedited reports as defined above) is included in the final study report to be submitted to both US FDA and Health Canada at the closure of the protocol.

8.6.3 Reporting SAEs to the DSMB

The NIAID and ITN will provide the DSMB with data of all SAEs on an ongoing basis, including quarterly reports of all SAEs.

8.6.4 Reporting Pregnancy

The principal investigator should be informed immediately of any pregnancy and all available pregnancy information should be entered into the electronic data capture (EDC) system within 24 hours of becoming aware of the event. The investigator should counsel the participant and discuss the risks of continuing with the pregnancy and the possible effects on the fetus. Monitoring of the participant should continue until the conclusion of the pregnancy. Follow-up information detailing the outcome of the pregnancy should be entered into the EDC system as it becomes available. Any premature termination of the pregnancy will be reported.
Pregnancies are tracked as SAEs for tracking purposes only. Any pregnancy complication or elective termination of a pregnancy for medical reasons will be recorded as an AE or SAE as described in sections 8.4 and 8.6, respectively.

9. Statistical Considerations and Analytical Plan

9.1 ANALYSIS SAMPLES

Intent to treat (ITT) sample will be defined as all participants who are found to be eligible after the initial 12 week lead-in period and undergo random assignment.
Per protocol (PP) sample will be defined as treated participants in the ITT sample who receive study regimen, as defined below, until 39 weeks:

- Receive 2 injections of ustekinumab during lead-in period.
- Receive 2 injections of ustekinumab or ustekinumab placebo during the treatment period.
- Receive at least 24 of the 28 abatacept or abatacept placebo injections prescribed.

Safety sample (SS) will be defined as all participants who receive at least one dose of study treatment after enrollment into the study.

9.2 ANALYSIS OF ENDPOINTS

9.2.1 Drop-Outs

The analysis of the primary and secondary endpoints will treat subjects who drop out of the study prior to week 88 as having experienced a psoriasis relapse at the time of study withdrawal. Sensitivity analyses will be performed to assess the effect of the drop-out assumptions on the analysis results by repeating the primary endpoint analysis but with different assumptions applied to the drop-out subjects. The primary endpoint analysis will be repeated, but with drop-out subjects assumed to have not experienced a psoriasis relapse at the time of study withdrawal, and with drop-out subjects as having a missing psoriasis relapse status.

9.2.2 Primary Endpoint

The proportion of participants who experience a psoriasis relapse at any evaluation between week 12 and week 88, as defined in section 3.3.1, will be analyzed for the ITT and PP samples using a logistic regression model with the participant's relapse status (relapse or no relapse) as the dependent variable and treatment as the independent variable. PASI score at Visit 0 (12-20 or >20) will be used as a covariate in the logistic regression model due to the inclusion of this variable as a stratification factor in the randomization. The ITT sample analysis will be the primary endpoint.

The duration of disease prior to screening (i.e. time from onset of psoriasis to screening visit) will also be used as an additional covariate in the logistic regression model.

9.2.3 Secondary Endpoints

- The proportion of participants who experience a psoriasis disease relapse at any evaluation between week 12 and week 40, as defined in section 3.3.2, will be analyzed for the ITT and PP samples using a logistic regression model similar to the one used in the primary endpoint analysis.
- The proportion of participants who experience a psoriasis disease relapse at any evaluation between week 28 and week 88, as defined in Section 3.3.2, will be analyzed for the ITT and PP samples using a logistic regression model similar to the one used in the primary endpoint analysis. Due to inherent bias, caution will be exercised in interpreting the results.
- The proportion of participants who experience a psoriasis disease relapse at any evaluation between week 40 and week 88, as defined in section 3.3.2, will be

analyzed for the ITT and PP samples using a logistic regression model similar to the one used in the primary endpoint analysis. Due to inherent bias, caution will be exercised in interpreting the results.

- The length of time until psoriasis relapse, as defined in section 3.3.2, in the intervals from week 12 to 40 and from week 12 to 88 will be analyzed for the ITT and PP samples using a survival analysis model. The exact time of relapse will not be known precisely. It will only be known to have occurred at some point since the previous evaluation visit. Thus, the grouped survival model [78, 79] will be applied using a logistic regression approach. The same covariates as described above will be included. The predicted survival curves for the 2 treatment sequences will be derived from this model.
- The proportions of participants who are classified as cleared or minimal in the PGA score at week 40 and at week 88, as defined in section 3.3.2, will be analyzed for the ITT and PP samples using a logistic regression model similar to the one used in the primary endpoint analysis. The participant's PGA score (0 or 1 vs. 2+) will be the dependent variable in the model. PGA scores assessed at the follow-up Visit D will not be included in this endpoint, and will be analyzed separately (section 9.2.5).
- The change in Dermatology Life Quality Index (DLQI) at week 40 and at week 88, as defined in section 3.3.2, will be analyzed for the ITT and PP samples using an analysis of covariance model (ANCOVA) with the change in DLQI score as the dependent variable, treatment as the independent variable, and baseline DLQI score, screening PASI score and disease duration at screening as covariates.

Table 2 summarizes the samples and analysis week intervals for which each secondary endpoint will be analyzed.

Table 2 Primary/Secondary Endpoints and Analysis Samples

Primary / Secondary Endpoints	Analysis Week Interval	ITT	PP	SS
Proportion Psoriasis Relapse	12 to 88	X	X	
	12 to 40	X	X	
	28 to 88	X	X	
	40 to 88	X	X	
Time to Psoriasis Relapse	12 to 40	X	X	
	12 to 88	X	X	
Physician Global Assessment	12 to 40	X	X	
	12 to 88	X	X	
Dermatology Life Quality Index	12 to 40	X	X	
	12 to 88	X	X	
Safety Analysis	12 to 88			X

9.2.4 Safety Analysis

Descriptive analysis will be performed on the safety sample. AEs collected at the follow-up Visit D will not be included in the safety analysis, and will be analyzed separately (section 9.2.5). Following standard ITN practice, safety information will not be imputed.

Safety will be analyzed in each treatment group through the reporting of AEs, vital signs, physical examinations, and changes in routine laboratory values.
All AEs will be classified by body system and preferred term, according to a standardized thesaurus: Medical Dictionary for Regulatory Activities (MedDRA). The severity of AEs will be classified using the NCI-CTCAE toxicity scale. The total number of events and the number of participants experiencing AEs will be summarized by body system and preferred term for each treatment group and overall.

Adverse events will also be summarized by maximum severity and relationship to the study drug for each treatment group and overall. Separate data listings will be provided for serious AEs, treatment-related AEs and AEs leading to study discontinuation.

Abnormal vital signs, physical examinations results, and laboratory values that are deemed clinically significant by the investigators will be graded by the NCI-CTCAE toxicity scale and reported as AEs.

For each treatment group and overall, physical examination results will be summarized by body system and visit.

Descriptive statistics of laboratory values and the change from baseline of laboratory values will be presented for each treatment group and overall. Laboratory measurements include serum chemistry, urinalysis and hematology.

9.2.5 Follow-up Visit Analysis

A separate descriptive analysis will be performed on the data collected at the follow-up visit D (AEs, PGA scores, and concomitant medications).

9.2.6 Medical History

Medical history within the past 12 months—including the existence of current signs and symptoms—will be collected for each body system.

9.2.7 Use of Medications

All medications taken by or administered to study participants beginning 30 days before enrollment and continuing throughout the study will be collected. All medications used will be coded according to the World Health Organization (WHO) drug dictionary. The number and percentage of participants receiving prior and concomitant medications/therapies will be presented overall and by medication class.

9.3 SAMPLE SIZE

The required study size is driven by the treatment comparison during the interval from Week 12-88. Based on results of the PHOENIX 1 study [3], 80% of participants randomized at week 12 to ustekinumab, and subsequently discontinued from study medication at week 40, are expected to relapse by week 88. A rate of 50% relapse is expected in participants randomized to abatacept at week 12, for a differential of 30 percentage points. The type-I error was set to $\alpha = .05$ (two-sided) and the type-II error rate was set to $\beta = 0.20$ representing power at 80%. Assuming equal allocation, and applying standard techniques for binary outcomes [80], a sample of 39 participants is required in each treatment group, for a total of 78 randomized participants. This is rounded up to 80 participants. As discussed above in section 9.2.1, drop-outs are considered to have relapsed, so there is no need to inflate the sample size to take drop-outs into consideration. To be eligible for the randomized phase, participants must respond to ustekinumab therapy during the lead-in phase. Considering the two PHOENIX studies [3, 4], a response rate between 67.9% and 71.1% can be expected in this study. Thus, it is anticipated that a total of $80 \div 0.679 = 118$ participants will be enrolled into the study. This is rounded up to 120 participants. To provide some protection against incorrect modeling assumptions, the response rate will be monitored during the study. If it falls below 67.9%, more participants will be recruited to ensure that 80 participants are ultimately randomized at Week 12. This notwithstanding, no more than 140 participants will be enrolled into the lead-in phase.

9.4 REPORTING DEVIATIONS FROM THE ORIGINAL STATISTICAL PLAN

The principal features of both the study design and the plan for statistical data analysis are outlined in this protocol and in the statistical analysis plan (SAP). Any change in these features requires either a protocol or an SAP amendment, which is subject to

review by the DSMB, the study sponsor(s), and the health authorities. These changes will be described in the final study report as appropriate.

10. Access to Source Data/Documents

The investigational sites participating in this study will maintain the highest degree of confidentiality permitted for the clinical and research information obtained from participants in this clinical trial. Medical and research records should be maintained at each site in the strictest confidence. However, as a part of the quality assurance and legal responsibilities of an investigation, the investigational sites must permit authorized representatives of the ITN, sponsor, and health authorities to examine (and to copy when required by applicable law) clinical records for the purposes of quality assurance reviews, audits, and evaluation of the study safety and progress. Unless required by the laws permitting copying of records, only the coded identity associated with documents or other participant data may be copied (and any personally identifying information must be obscured). Authorized representatives as noted above are bound to maintain the strict confidentiality of medical and research information that may be linked to identified individuals. The investigational sites will normally be notified in advance of auditing visits.

11. Quality Control and Quality Assurance

The principal investigator is required to keep accurate records to ensure that the conduct of the study is fully documented. The principal investigator is required to ensure that all eCRFs are completed for every participant entered in the trial.
The sponsor is responsible for regular inspection of the conduct of the trial, for verifying adherence to the protocol, and for confirming the completeness, consistency, and accuracy of all documented data.

The eCRFs will be completed online via a web-based EDC system that has been validated and is compliant with Part 11 Title 21 of the Code of Federal Regulations. Some data requirements will be addressed outside the EDC using statistical analysis system (SAS®) software. Data queries will be issued and resolved within the EDC system or SAS®.

Study staff at the site will enter information into the electronic CRFs, and the data will be stored remotely at a central database. Data quality will be ensured through the EDC system's continuous monitoring of data and real-time detection and correction of errors. All elements of data entry (i.e. time, date, verbatim text, and the name of the person performing the data entry) will be recorded in an electronic audit trail to allow all changes in the database to be monitored and maintained in accordance with US and Canadian regulations.

Study staff will enter data from a study visit on the relevant eCRFs within 3 days following the visit or the time when data become available.

Skin photographs will be transferred to the SDCC using a secure file transfer process and stored in a password-protected environment.

12. Ethical Considerations and Compliance with Good Clinical Practice

12.1 STATEMENT OF COMPLIANCE

This trial will be conducted in compliance with the protocol, current GCP guidelines — adopting the principles of the Declaration of Helsinki — and all applicable regulatory requirements.

Prior to study initiation, the protocol and the informed consent documents will be reviewed and approved by the sponsor and the appropriate health authorities and ethics review committee or institutional review board (IRB). Any amendments to the protocol or consent materials must also be approved by the Sponsor, the IRB or ethics review committee, and submitted to FDA before they are implemented in the US. Amendments in Canada must be approved by the relevant ethics committees and Health Canada before being implemented.

12.2 INFORMED CONSENT

The informed consent form is a means of providing information about the trial to a prospective participant and allows for an informed decision about participation in the study. All participants must read, sign, and date a consent form before participating in the study, taking the study drug, and/or undergoing any study-specific procedures. If a participant does not speak and read English, the consent materials must be translated into the appropriate language.

The informed consent form must be updated or revised whenever important new safety information is available, whenever the protocol is amended, and/or whenever any new information becomes available that may affect participation in the trial.

A copy of the informed consent will be given to a prospective participant for review. The study investigator, in the presence of a witness, will review the consent and answer questions. The participant will be informed that participation is voluntary and that he/she may withdraw from the study at any time, for any reason.

12.3 PRIVACY AND CONFIDENTIALITY

A participant's privacy and confidentiality will be respected throughout the study. Each participant will be assigned a sequential identification number. This number, rather than the participant's name, will be used to collect, store, and report participant information.

13. Publication Policy

The ITN policy on publication of study results will apply to this study. Authorized participants may find details regarding the policy statement on the ITN internet website at http://www.immunetolerance.org.

14. References

1. Menter, A., et al., Guidelines of care for the management of psoriasis and psoriatic arthritis: Section 1. Overview of psoriasis and guidelines of care for the treatment of psoriasis with biologics. J Am Acad Dermatol, 2008. **58**(5): p. 826-50.
2. Menter, A., et al., Guidelines of care for the management of psoriasis and psoriatic arthritis: section 6. Guidelines of care for the treatment of psoriasis and psoriatic arthritis: case-based presentations and evidence-based conclusions. J Am Acad Dermatol, 2011. **65**(1): p. 137-74.
3. Leonardi, C.L., et al., Efficacy and safety of ustekinumab, a human interleukin-12/23 monoclonal antibody, in patients with psoriasis: 76-week results from a randomised, double-blind, placebo-controlled trial (PHOENIX 1). Lancet, 2008. **371**(9625): p. 1665-74.
4. Papp, K.A., et al., Efficacy and safety of ustekinumab, a human interleukin-12/23 monoclonal antibody, in patients with psoriasis: 52-week results from a randomised, double-blind, placebo-controlled trial (PHOENIX 2). Lancet, 2008. **371**(9625): p. 1675-84.
5. Griffiths, C.E., et al., Comparison of ustekinumab and etanercept for moderate-to-severe psoriasis. N Engl J Med, 2010. **362**(2): p. 118-28.
6. Mueller, D.L., M.K. Jenkins, and R.H. Schwartz, Clonal expansion versus functional clonal inactivation: a costimulatory signalling pathway determines the outcome of T cell antigen receptor occupancy. Annu Rev Immunol, 1989. **7**: p. 445-80.
7. Bour-Jordan, H., et al., Intrinsic and extrinsic control of peripheral T-cell tolerance by costimulatory molecules of the CD28/ B7 family. Immunol Rev, 2011. **241**(1): p. 180-205.
8. Watanabe, N. and H. Nakajima, *Coinhibitory molecules in autoimmune diseases.* Clin Dev Immunol, 2012. **2012**: p. 269756.
9. Abrams, J.R., et al., CTLA4Ig-mediated blockade of T-cell costimulation in patients with psoriasis vulgaris. J Clin Invest, 1999. **103**(9): p. 1243-52.
10. Mease, P., et al., Abatacept in the treatment of patients with psoriatic arthritis: results of a six-month, multicenter, randomized, double-blind, placebo-controlled, phase II trial. Arthritis Rheum, 2011. **63**(4): p. 939-48.
11. Abrams, J.R., et al., Blockade of T lymphocyte costimulation with cytotoxic T lymphocyte-associated antigen 4-immunoglobulin (CTLA4Ig) reverses the cellular pathology of psoriatic plaques, including the activation of keratinocytes, dendritic cells, and endothelial cells. J Exp Med, 2000. **192**(5): p. 681-94.
12. Krueger, J., K Li, F Baribaud, M Suarez-Farinas, C Brodmerkel, The molecular profile of psoriatic skin in responders to ustekinumab or etanercept following twelve weeks of treatment: Results from the ACCEPT Trial. Abstract, World Psoriasis and Psoriatic Arthritis Conference, 2009.
13. Krueger, J., K Li, F Baribaud, M Suarez-Farinas, C Brodmerkel, Determining the extent to which clinically effective treatment, ustekinumab or etanercept, reverses the molecular disease profile of psoriatic skin: Comparisons of lesional, non-lesional and normal skin. Abstract, Society of Investigative Dermatology, 2010.
14. Krueger, J.G., T Kikuchi, A Fidelus-Gort, R Khatcherian, I Novitskaya, C Brodmerkel, *The Immune Phenotype of Therapeutic IL-12/IL-23 Blockade in*

Psoriasis Patients Treated with Ustekinumab. Abstract, Society of Investigative Dermatology, 2009.
15. Haider, A.S., et al., Identification of cellular pathways of "type 1," Th17 T cells, and TNF- and inducible nitric oxide synthase-producing dendritic cells in autoimmune inflammation through pharmacogenomic study of cyclosporine A in psoriasis. J Immunol, 2008. **180**(3): p. 1913-20.
16. Johnson-Huang, L.M., et al., Post-therapeutic relapse of psoriasis after CD11a blockade is associated with T cells and inflammatory myeloid DCs. PLoS One, 2012. **7**(2): p. e30308.
17. Hong, K., et al., IL-12, independently of IFN-gamma, plays a crucial role in the pathogenesis of a murine psoriasis-like skin disorder. J Immunol, 1999. **162**(12): p. 7480-91.
18. Leonard, J.P., K.E. Waldburger, and S.J. Goldman, Prevention of experimental autoimmune encephalomyelitis by antibodies against interleukin 12. J Exp Med, 1995. **181**(1): p. 381-6.
19. Davidson, N.J., et al., IL-12, but not IFN-gamma, plays a major role in sustaining the chronic phase of colitis in IL-10-deficient mice. J Immunol, 1998. **161**(6): p. 3143-9.
20. Malfait, A.M., et al., Blockade of IL-12 during the induction of collagen-induced arthritis (CIA) markedly attenuates the severity of the arthritis. Clin Exp Immunol, 1998. **111**(2): p. 377-83.
21. Cua, D.J., et al., Interleukin-23 rather than interleukin-12 is the critical cytokine for autoimmune inflammation of the brain. Nature, 2003. **421**(6924): p. 744-8.
22. Gran, B., et al., IL-12p35-deficient mice are susceptible to experimental autoimmune encephalomyelitis: evidence for redundancy in the IL-12 system in the induction of central nervous system autoimmune demyelination. J Immunol, 2002. **169**(12): p. 7104-10.
23. Murphy, C.A., et al., Divergent pro- and antiinflammatory roles for IL-23 and IL-12 in joint autoimmune inflammation. J Exp Med, 2003. **198**(12): p. 1951-7.
24. Langrish, C.L., et al., IL-23 drives a pathogenic T cell population that induces autoimmune inflammation. J Exp Med, 2005. **201**(2): p. 233-40.
25. Benson, J.M., et al., Therapeutic targeting of the IL-12/23 pathways: generation and characterization of ustekinumab. Nat Biotechnol, 2011. **29**(7): p. 615-24.
26. Judge, T.A., et al., *The in vivo mechanism of action of CTLA4Ig.* J Immunol, 1996. **156**(6): p. 2294-9.
27. Schweitzer, A.N. and A.H. Sharpe, Studies using antigen-presenting cells lacking expression of both B7-1 (CD80) and B7-2 (CD86) show distinct requirements for B7 molecules during priming versus restimulation of Th2 but not Th1 cytokine production. J Immunol, 1998. **161**(6): p. 2762-71.
28. Newell, K.A., et al., Cutting edge: blockade of the CD28/B7 costimulatory pathway inhibits intestinal allograft rejection mediated by CD4+ but not CD8+ T cells. J Immunol, 1999. **163**(5): p. 2358-62.
29. Zimmermann, C., et al., *Antiviral immune responses in CTLA4 transgenic mice.* J Virol, 1997. **71**(3): p. 1802-7.
30. Linsley, P.S., et al., Immunosuppression in vivo by a soluble form of the CTLA-4 T cell activation molecule. Science, 1992. **257**(5071): p. 792-5.
31. Reddy, B., et al., The effect of CD28/B7 blockade on alloreactive T and B cells after liver cell transplantation. Transplantation, 2001. **71**(6): p. 801-11.

32. Fredriksson, T. and U. Pettersson, *Severe psoriasis--oral therapy with a new retinoid.* Dermatologica, 1978. **157**(4): p. 238-44.
33. Ashcroft, D.M., et al., Clinical measures of disease severity and outcome in psoriasis: a critical appraisal of their quality. Br J Dermatol, 1999. **141**(2): p. 185-91.
34. Kimball, A.B., et al., Long-term efficacy of ustekinumab in patients with moderate-to-severe psoriasis: results from the PHOENIX 1 trial through up to 3 years. Br J Dermatol, 2012. **166**(4): p. 861-72.
35. Gelfand, J.M., et al., Epidemiology of psoriatic arthritis in the population of the United States. J Am Acad Dermatol, 2005. **53**(4): p. 573.
36. Lloyd, P., C. Ryan, and A. Menter, *Psoriatic arthritis: an update.* Arthritis, 2012. **2012**: p. 176298.
37. Gottlieb, A., et al., Ustekinumab, a human interleukin 12/23 monoclonal antibody, for psoriatic arthritis: randomised, double-blind, placebo-controlled, crossover trial. Lancet, 2009. **373**(9664): p. 633-40.
38. Felson, D.T., et al., American College of Rheumatology. Preliminary definition of improvement in rheumatoid arthritis. Arthritis Rheum, 1995. **38**(6): p. 727-35.
39. Kavanaugh, A., et al., Effect of ustekinumab on physical function and health-related quality of life in patients with psoriatic arthritis: a randomized, placebo-controlled, phase II trial. Curr Med Res Opin, 2010. **26**(10): p. 2385-92.
40. Sandborn, W.J., et al., A randomized trial of Ustekinumab, a human interleukin-12/23 monoclonal antibody, in patients with moderate-to-severe Crohn's disease. Gastroenterology, 2008. **135**(4): p. 1130-41.
41. Sandborn, W.J., et al., Ustekinumab induction and maintenance therapy in refractory Crohn's disease. N Engl J Med, 2012. **367**(16): p. 1519-28.
42. Moreland, L.W., et al., Costimulatory blockade in patients with rheumatoid arthritis: a pilot, dose-finding, double-blind, placebo-controlled clinical trial evaluating CTLA-4Ig and LEA29Y eighty-five days after the first infusion. Arthritis Rheum, 2002. **46**(6): p. 1470-9.
43. Kremer, J.M., et al., Treatment of rheumatoid arthritis by selective inhibition of T-cell activation with fusion protein CTLA4Ig. N Engl J Med, 2003. **349**(20): p. 1907-15.
44. Kremer, J.M., et al., Effects of abatacept in patients with methotrexate-resistant active rheumatoid arthritis: a randomized trial. Ann Intern Med, 2006. **144**(12): p. 865-76.
45. Genovese, M.C., et al., Abatacept for rheumatoid arthritis refractory to tumor necrosis factor alpha inhibition. N Engl J Med, 2005. **353**(11): p. 1114-23.
46. Genovese, M.C., et al., Longterm safety and efficacy of abatacept through 5 years of treatment in patients with rheumatoid arthritis and an inadequate response to tumor necrosis factor inhibitor therapy. J Rheumatol, 2012. **39**(8): p. 1546-54.
47. Ruperto, N., et al., Abatacept in children with juvenile idiopathic arthritis: a randomised, double-blind, placebo-controlled withdrawal trial. Lancet, 2008. **372**(9636): p. 383-91.
48. Ruperto, N., et al., Long-term safety and efficacy of abatacept in children with juvenile idiopathic arthritis. Arthritis Rheum, 2010. **62**(6): p. 1792-802.
49. Orban, T., et al., Co-stimulation modulation with abatacept in patients with recent-onset type 1 diabetes: a randomised, double-blind, placebo-controlled trial. Lancet, 2011. **378**(9789): p. 412-9.

50. Sandborn, W.J., et al., *Abatacept for Crohn's disease and ulcerative colitis.* Gastroenterology, 2012. **143**(1): p. 62-69 e4.
51. Viglietta, V., et al., CTLA4Ig treatment in patients with multiple sclerosis: an open-label, phase 1 clinical trial. Neurology, 2008. **71**(12): p. 917-24.
52. *Stelera [Package Insert].* Janssen Biotech, Inc., Horsham, Pennsylvania, January 2013.
53. Gordon, K.B., et al., Long-term safety experience of ustekinumab in patients with moderate to severe psoriasis (Part II of II): results from analyses of infections and malignancy from pooled phase II and III clinical trials. J Am Acad Dermatol, 2012. **66**(5): p. 742-51.
54. Lebwohl, M., et al., Long-term safety experience of ustekinumab in patients with moderate-to-severe psoriasis (Part I of II): results from analyses of general safety parameters from pooled Phase 2 and 3 clinical trials. J Am Acad Dermatol, 2012. **66**(5): p. 731-41.
55. Young, L. and D. Czarnecki, The rapid onset of multiple squamous cell carcinomas in two patients commenced on ustekinumab as treatment of psoriasis. Australas J Dermatol, 2012. **53**(1): p. 57-60.
56. Burmester, G.R., et al., Adalimumab safety and mortality rates from global clinical trials of six immune-mediated inflammatory diseases. Ann Rheum Dis, 2009. **68**(12): p. 1863-9.
57. Papp, K.A., et al., Assessment of the long-term safety and effectiveness of etanercept for the treatment of psoriasis in an adult population. J Am Acad Dermatol, 2012. **66**(2): p. e33-45.
58. Reich, K., et al., Cardiovascular safety of ustekinumab in patients with moderate to severe psoriasis: results of integrated analyses of data from phase II and III clinical studies. Br J Dermatol, 2011. **164**(4): p. 862-72.
59. Ryan, C., et al., Association between biologic therapies for chronic plaque psoriasis and cardiovascular events: a meta-analysis of randomized controlled trials. JAMA, 2011. **306**(8): p. 864-71.
60. Langley, R.G., et al., Safety results from a pooled analysis of randomized, controlled phase II and III clinical trials and interim data from an open-label extension trial of the interleukin-12/23 monoclonal antibody, briakinumab, in moderate to severe psoriasis. J Eur Acad Dermatol Venereol, 2012.
61. *Orencia [Package Insert].* Bristol-Meyers Squibb Company, Princeton, New Jersey, December 2011.
62. Khraishi, M., A. Russell, and W.P. Olszynski, *Safety profile of abatacept in rheumatoid arthritis: a review.* Clin Ther, 2010. **32**(11): p. 1855-70.
63. Sibilia, J. and R. Westhovens, Safety of T-cell co-stimulation modulation with abatacept in patients with rheumatoid arthritis. Clin Exp Rheumatol, 2007. **25**(5 Suppl 46): p. S46-56.
64. Weinblatt, M., et al., Safety of the selective costimulation modulator abatacept in rheumatoid arthritis patients receiving background biologic and nonbiologic disease-modifying antirheumatic drugs: A one-year randomized, placebo-controlled study. Arthritis Rheum, 2006. **54**(9): p. 2807-16.
65. Schiff, M., et al., The 6-month safety and efficacy of abatacept in patients with rheumatoid arthritis who underwent a washout after anti-tumour necrosis factor therapy or were directly switched to abatacept: the ARRIVE trial. Ann Rheum Dis, 2009. **68**(11): p. 1708-14.

66. Finlay, A.Y. and G.K. Khan, Dermatology Life Quality Index (DLQI)--a simple practical measure for routine clinical use. Clin Exp Dermatol, 1994. **19**(3): p. 210-6.
67. Basra, M.K., et al., The Dermatology Life Quality Index 1994-2007: a comprehensive review of validation data and clinical results. Br J Dermatol, 2008. **159**(5): p. 997-1035.
68. Kaine, J., et al., Evaluation of abatacept administered subcutaneously in adults with active rheumatoid arthritis: impact of withdrawal and reintroduction on immunogenicity, efficacy and safety (phase Iiib ALLOW study). Ann Rheum Dis, 2012. **71**(1): p. 38-44.
69. Schiff, M., Alten, R., Weinblatt, M., Nash, P., Fleischmann, R., Durez, P., Kaine, J., Delaet, I., Kelly, S., Maldonado, M., Patel, S., Genovese, M.C., *Weekly Subcutaneous Abatacept Confers Comparable Onset of Treatment Response and Magnitude of Efficacy Improvement Over 6 Months When Administered with or without an Intravenous Abatacept Loading Dose.* American College of Rheumatology Annual Meeting Abstract 2547, 2012.
70. Husni, M.E., et al., The PASE questionnaire: pilot-testing a psoriatic arthritis screening and evaluation tool. J Am Acad Dermatol, 2007. **57**(4): p. 581-7.
71. Dominguez, P.L., et al., Validity, reliability, and sensitivity-to-change properties of the psoriatic arthritis screening and evaluation questionnaire. Arch Dermatol Res, 2009. **301**(8): p. 573-9.
72. Krueger, J.G., et al., IL-17A is essential for cell activation and inflammatory gene circuits in subjects with psoriasis. J Allergy Clin Immunol, 2012. **130**(1): p. 145-54 e9.
73. Suarez-Farinas, M., et al., Expanding the Psoriasis Disease Profile: Interrogation of the Skin and Serum of Patients with Moderate-to-Severe Psoriasis. J Invest Dermatol, 2012.
74. Papp, K.A., et al., Brodalumab, an anti-interleukin-17-receptor antibody for psoriasis. N Engl J Med, 2012. **366**(13): p. 1181-9.
75. Papp, K.A., et al., Anti-IL-17 Receptor Antibody AMG 827 Leads to Rapid Clinical Response in Subjects with Moderate to Severe Psoriasis: Results from a Phase I, Randomized, Placebo-Controlled Trial. J Invest Dermatol, 2012. **132**(10): p. 2466-9.
76. Zaba, L.C., et al., Effective treatment of psoriasis with etanercept is linked to suppression of IL-17 signaling, not immediate response TNF genes. J Allergy Clin Immunol, 2009. **124**(5): p. 1022-10 e1-395.
77. Suarez-Farinas, M., et al., Resolved psoriasis lesions retain expression of a subset of disease-related genes. J Invest Dermatol, 2011. **131**(2): p. 391-400.
78. Efron, B., Logistic regression, survival analysis, and the Kaplan-Meier Curve. J Amer Stat Assoc, 1988. **83**: p. 414-25.
79. D'Agostino, R.B., et al., Relation of pooled logistic regression to time dependent Cox regression analysis: the Framingham Heart Study. Stat Med, 1990. **9**(12): p. 1501-15.
80. Machin, D., Campbell, M.J., Tan, S.B., Tan, S.H., *Samples Size Tables for Clinical Studies*. 3rd. ed2009, Oxford: Blackwell Science Ltd.

APPENDIX 1. SCHEDULE OF EVENTS

**Study Week**		**0**	**4**	**8**	**12**	**16**	**20**	**24**	**28**	**32**	**36**	**39**
**Visit Number**	**-1**	**0**	**1**	**2**	**3**	**4**	**5**	**6**	**7**	**8**	**9**	**---**
**GENERAL ASSESSMENTS**												
Informed Consent	x											
Randomization					x							
Demographics (age and ethnicity)	x											
Medical History	x											
Psoriasis History	x											
Comprehensive Physical Exam	x											
Brief Physical Exam		x	x	x	x	x	x	x	x	x	x	
Adverse Events	x	x	x	x	x	x	x	x	x	x	x	
Concomitant Medications	x	x	x	x	x	x	x	x	x	x	x	
Vital Signs	x	x	x	x	x	x	x	x	x	x	x	
**CLINICAL LABORATORY ASSESSMENTS**												
Serum Chemistry (AST, ALT, bilirubin, alkaline phosphatase, random glucose)	x				x			x				
Hematology	x				x			x	x			
Serum Creatinine	x				x			x				
RPR	x											
Serum HCG	x											
QuantiFERON -TB Gold	x											
Urinalysis	x											
STAT Urine HCG		x	x	x	x	x	x	x	x	x	x	
HIV	x											
Hepatitis B & C	x											
Chest X-ray	x											
**PSORIASIS ASSESSMENTS**												
PASI[1]	x	x	x	x	x	x	x	x	x	x	x	
PGA		x			x			x				
DLQI		x			x			x				
PASE	x	x	x	x	x	x	x	x	x	x	x	
Skin Photography[3]		x			x							
**STUDY DRUG ADMINISTRATION**												
Ustekinumab		x	x									
Ustekinumab/ Ustekinumab Placebo						x			x			
Abatacept/Abatacept Placebo[2]					x	x	x	x	x	x	x	x
**MECHANISTIC LABORATORY ASSESSMENTS**												
Lesional Punch Skin Biopsy		x			x			x[4]				
Non-lesional Punch Skin Biopsy		x										
Plasma/Serum Assays		x			x			x				
PBMC Assays		x			x			x				
RNA Assays		x			x			x				

**Study Week**	**40**	**44**	**48**	**52**	**56**	**60**	**64**	**68**	**72**	**76**	**80**	**84**	**88**		
**Visit Number**	**10**	**11**	**12**	**13**	**14**	**15**	**16**	**17**	**18**	**19**	**20**	**21**	**22**	**D[5]**	**U[7]**

[1] Each PASI assessment will include the Body Surface Area psoriasis involvement.
[2] SQ Abatacept is given weekly from week 12-39.
[3] Skin Photography is optional.
[4] Lesional Punch Skin Biopsy is optional at week 24 visit.

**GENERAL ASSESSMENTS**															
Informed Consent															
Randomization															
Demographics (age and ethnicity)															
Medical History															
Psoriasis History															
Comprehensive Physical Exam															
Brief Physical Exam	x	x	x	x	x	x	x	x	x	x	x	x	x		x
Adverse Events	x	x	x	x	x	x	x	x	x	x	x	x	x	x	x
Concomitant Medications	x	x	x	x	x	x	x	x	x	x	x	x	x	x	x
Vital Signs	x	x	x	x	x	x	x	x	x	x	x	x	x		x
**CLINICAL LABORATORY ASSESSMENTS**															
Serum Chemistry (AST, ALT, bilirubin, alkaline phosphatase, random glucose)	x								x						x
Hematology	x				x				x				x		x
Serum Creatinine	x														
RPR															
Serum HCG															
QuantiFERON -TB Gold															
Urinalysis															
STAT Urine HCG				x									x		x
HIV															
Hepatitis B & C															
Chest X-ray															
**PSORIASIS ASSESSMENTS**															
PASI[1]	x	x	x	x	x	x	x	x	x	x	x	x	x		x
PGA	x				x				x				x	x	
DLQI	x				x				x				x		
PASE	x	x	x	x	x	x	x	x	x	x	x	x	x		x
Skin Photography[3]	x												x		
**STUDY DRUG ADMNISTRATION**															
Ustekinumab															
Ustekinumab/ Ustekinumab Placebo															
Abatacept/Abatacept Placebo[2]															
**MECHANISTIC LABORATORY ASSESSMENTS**															
Lesional Punch Skin Biopsy	x												x		
Non-lesional Punch Skin Biopsy	x												x[6]		
Plasma/Serum Assays	x				x				x				x		
PBMC Assays	x				x				x				x		
RNA Assays	x				x				x				x		

[5] The follow-up visit D will occur in randomized participants 12 weeks after discontinuation of study treatment or study observation (see section 5.3).
Visit D will occur 12 weeks after Visit 22 for participants who complete Visit 22.
[6] The week 88 non-lesional biopsy is considered optional.
[7] Unscheduled visits may be performed to document any symptoms or to document a psoriasis relapse during the study (see section 6.1.2).

STATISTICAL ANALYSIS PLAN
Final Version
21 November 2017

Efficacy of Ustekinumab (Anti-IL-12/23) followed by Abatacept (CTLA4-Ig) for the Treatment of Psoriasis Vulgaris

PROTOCOL NUMBER ITN059AI

SPONSOR

Division of Allergy, Immunology, and Transplantation
National Institute of Allergy and Infectious Diseases – NIH
5601 Fishers Lane
Rockville, MD 20852
Telephone: (301) 496-5717
Fax: (301) 402-3573

PREPARED BY

Rho, Inc.
6330 Quadrangle Drive
Chapel Hill, NC 27517
Telephone: (919) 408-8000
Fax: (919) 408-0999

DOCUMENT VERSION CONTROL

Version Number	Date	Comments/Changes
1.0	21 November 2017	Final

TABLE OF CONTENTS

1. PROTOCOL SYNOPSIS 6

2. INTRODUCTION 10

3. GENERAL ANALYSIS AND REPORTING CONVENTIONS 11

4. ANALYSIS SAMPLES 13

5. STUDY SUBJECTS 14

5.1 Disposition of Subjects 14
5.2 Demographic and Other Baseline Characteristics 14

6. STUDY OPERATIONS 15

6.1 Protocol Deviations 15
6.2 Treatment Adherence 15
6.2.1 Ustekinumab 15
6.2.2 Abatacept 15

7. ENDPOINT EVALUATION 16

7.1 Overview of Efficacy Analysis Methods 16
7.1.1 Multicenter Studies 16
7.1.2 Assessment Time Windows 16
7.1.3 Summary of Primary and Secondary Efficacy Analysis Methods 16
7.2 Primary Endpoint 19
7.2.1 Computation of the Primary Endpoint 19
7.2.2 Primary Analysis of the Primary Endpoint 19
7.2.3 Sensitivity Analyses of the Primary Endpoint 20
7.3 Secondary Endpoints and Analyses 20
7.3.1 Psoriasis Relapse and PASI 20
7.3.2 Physician's Global Assessment (PGA) 22
7.3.3 Dermatology Life Quality Index (DLQI) 23

8. SAFETY EVALUATION 24

8.1 Overview of Safety Analysis Methods 24
8.2 Adverse Events 24
8.3 Deaths and Serious Adverse Events 26
8.4 Pregnancies 26
8.5 Clinical Laboratory Evaluation 26
8.6 Vital Signs, Physical Findings, and Other Observations Related to Safety 26
8.6.1 Vital Signs 26
8.6.2 Physical Examinations 26

9. OTHER ANALYSES 27

9.1 Psoriatic Arthritis Screening and Evaluation (PASE) 27
9.2 Use of Medications 27
9.3 Medical History 27
9.4 Assessments Collected at Follow-up Visit D 27
9.4.1 Physician's Global Assessment (PGA) 28
9.4.2 Adverse Events 28
9.4.3 Use of Medications 28

10. INTERIM ANALYSES AND DATA MONITORING 29

11. CHANGES TO THE ANALYSES PLANNED IN THE PROTOCOL........................30
11.1 Psoriasis Relapse and PASI secondary endpoint analysis 30
11.2 Dermatology Life Quality Index (DLQI) secondary endpoint analysis 30
12. REFERENCES 31
13. APPENDICES 32
13.1 Study Flow Chart 32
13.2 Schedule of Events 33

1. PROTOCOL SYNOPSIS

**Title**	Efficacy of Ustekinumab (Anti-IL-12/23) followed by Abatacept (CTLA4-Ig) for the Treatment of Psoriasis Vulgaris
**Sponsor**	NIAID
**Conducted by**	Immune Tolerance Network
**Protocol Chair**	James G. Krueger, MD, PhD
**Study Treatment**	Sequential treatment with open label ustekinumab, followed by either continued ustekinumab and abatacept placebo, or abatacept and ustekinumab placebo.
**Study Design**	This trial will be conducted as a prospective, randomized, double-blind, multicenter, active comparator study in a maximum of 140 participants with psoriasis vulgaris to assess the efficacy of abatacept in perpetuating the known treatment benefit of ustekinumab following ustekinumab withdrawal. Efficacy of abatacept to induce prolonged disease remission following cessation of study medication will also be assessed.<br><br>The study design has a lead-in period of weight-based ustekinumab treatment, with all participants receiving either 45 mg ustekinumab (≤ 100 kg) or 90 mg ustekinumab (> 100 kg) administered subcutaneously at weeks 0 and 4. At week 12, participants will be assessed for a PASI 75 response to ustekinumab. Participants who do not achieve a PASI 75 score will be discontinued from the investigation and permitted to seek standard therapy.<br><br>The week 12 PASI 75 ustekinumab responders will be randomized 1:1 to receive either abatacept plus ustekinumab placebo or continued ustekinumab plus abatacept placebo. The trial design requires a sample size of 80 randomized participants. Published results indicate that 67.9% of participants who receive ustekinumab are expected to achieve a PASI 75 or greater response by week 12. Therefore, 120 participants are expected to be required in order to randomize the 80 required participants. The week 12 PASI 75 response rate will be monitored during the study. If it falls below the expected rate, a maximum of 20 additional participants will be enrolled to ensure that 80 participants are randomized at week 12. The initial enrollment will be set at 120 participants, with a maximum enrollment, if necessary, of 140 participants.

	The abatacept treatment group will receive weekly subcutaneous injections of 125 mg abatacept from week 12 to week 39. The abatacept treatment group will also receive subcutaneous ustekinumab placebo at week 16 and week 28, corresponding to the ustekinumab dosing regimen.<br><br>The continued ustekinumab treatment group will receive subcutaneous injections of 45 mg ustekinumab (≤ 100 kg) or 90 mg ustekinumab (> 100 kg) at week 16 and week 28. The ustekinumab treatment group will also receive weekly subcutaneous injections of abatacept placebo from week 12 to week 39, corresponding to the abatacept dosing regimen.<br><br>Study medication will be discontinued following the week 39 abatacept or abatacept placebo injection, and participants will be observed for disease relapse from week 40 through week 88. Following relapse, participants will be discontinued from observation, and will be permitted to seek standard therapy. There will be a follow-up safety visit 12 weeks after discontinuation.<br><br>In addition to clinical observation, 6 millimeter punch skin biopsies will be obtained from lesional skin at weeks 0, 12, 24, 40, 88, and at the time of psoriasis relapse, and from non-lesional skin at weeks 0, 40, 88, and at the time of psoriasis relapse.
**Study Conduct**	The study will be conducted at multiple sites in the United States and Canada. The study participation duration will be up to 100 weeks, which includes a treatment phase of 39 weeks, an observation phase of up to 49 weeks, and a follow-up visit 12 weeks after the observation phase is complete.
**Primary Objective**	The primary objective of the study is to determine whether co-stimulatory blockade with abatacept (CTLA4Ig) will induce tolerance and prevent relapses after medication has been discontinued in psoriasis vulgaris when administered following a decrease in the inflammatory response induced with ustekinumab (anti-IL-12/23).
**Primary Endpoint**	The primary endpoint is the proportion of participants who experience a psoriasis relapse at any time between week 12 and week 88. Psoriasis relapse is defined as loss of ≥ 50% of the initial PASI improvement measured at week 12. The primary endpoint will be assessed in all randomized participants.

**Secondary Endpoints**	**Efficacy:**<br>1. The proportion of randomized participants who experience a psoriasis disease relapse prior to week 40.<br>2. The proportion of participants who experience a psoriasis disease relapse between week 28 and week 88.<br>3. The proportion of participants who experience a psoriasis disease relapse between week 40 and week 88.<br>4. Mean length of time after week 12 to psoriasis relapse.<br>5. Physician's Global Assessment (PGA) of cleared or minimal at week 40 and week 88.<br>6. Dermatology Life Quality Index (DLQI) at week 40 and week 88.<br><br>**Safety:**<br>1. Frequency and severity of all AEs and SAEs.
**Inclusion Criteria**	Patients *must meet all* of the following criteria to be eligible for this study:<br>1. Males or females aged 18-65 years with a diagnosis of plaque psoriasis for at least 6 months.<br>2. Baseline PASI score $\geq$ 12.<br>3. $\geq$ 10% body surface area psoriasis involvement.<br>4. Willingness to forgo other available psoriasis therapies, live vaccines, and pregnancy during the trial.<br>5. Ability and willingness to provide informed consent and comply with study requirements.
**Exclusion Criteria**	Patients who *meet any* of the following criteria will *not* be eligible for this study:<br>1. Non-plaque forms of psoriasis.<br>2. Grade 2 or 3 moderate to severe psoriatic arthritis not adequately managed with non-steroidal anti-inflammatory drugs (NSAIDs).<br>3. Myocardial infarction, unstable angina, cerebrovascular accident, or other significant cardiovascular event within the previous one year.<br>4. Chronic obstructive pulmonary disease.<br>5. Comorbid condition that requires regular systemic corticosteroid treatment.<br>6. History of malignancy, except treated basal cell skin carcinoma.<br>7. Treated basal cell skin carcinoma within the previous 5 years.<br>8. Severe, progressive, or uncontrolled renal, hepatic, hematological, gastrointestinal, pulmonary, cardiac, or

neurological disease, or any other medical condition that, in the investigator's opinion, places the participant at risk by participating in this study.

9. History of recent or ongoing uncontrolled bacterial, viral, fungal, or other opportunistic infections.
10. Evidence of infection with Hepatitis B Virus (HBV), Hepatitis C Virus (HCV), or Human Immunodeficiency Virus (HIV).
11. Positive QuantiFERON-TB Gold test. PPD tuberculin test may be substituted for QuantiFERON-TB Gold test.
12. Severe reaction or anaphylaxis to any human monoclonal antibody.
13. Any previous treatment with agents targeting IL-12 or IL-23, including ustekinumab.
14. Any previous treatment with abatacept.
15. Treatment with biologic agents within previous 3 months prior to visit 0, including adalimumab, etanercept, and infliximab.
16. Treatment with immunosuppressive medications, including methotrexate, cyclosporine, oral retinoids, prednisone, or phototherapy within previous 4 weeks prior to visit 0.
17. Topical psoriasis treatment within previous 2 weeks prior to visit 0, including topical corticosteroids, vitamin D analogues, retinoids, calcineurin inhibitors, salicylic acid, and coal tar.
18. Investigational study medication within previous 6 months prior to visit 0.
19. Liver function test (aspartate aminotransferase [AST], alanine aminotransferase [ALT], or alkaline phosphatase) results that are ≥ 2x the upper limit of normal (ULN).
20. Serum creatinine ≥ 2x the ULN.
21. Any of the following hematologic abnormalities, confirmed by repeat test at least 1 week apart:
    a. White blood count <3,000/μL or >14,000/μL;
    b. Lymphocyte count <1,000/μL;
    c. Neutrophil count <1,500/μL;
    d. Platelet count <150,000 /μL; *or*
    e. Hemoglobin <10 g/dL.
22. Females who are pregnant, lactating, planning on pregnancy during the study period, or unwilling to use a medically acceptable method of birth control.
23. Receipt of a live vaccine (e.g., varicella, measles, mumps, rubella, cold-attenuated intranasal influenza vaccine, and smallpox) in the previous 6 weeks prior to visit 0.
24. BCG vaccines in the previous one year.

2. INTRODUCTION

This statistical analysis plan (SAP) only includes analyses related to the clinical endpoints outlined in the protocol. Mechanistic analyses will be performed at the Immune Tolerance Network (ITN), and a separate analysis plan will be created to detail the planned analyses. Relevant clinical data from the study will be submitted to the ITN Biomarker and Discovery Research (BDR) to augment the mechanistic analyses.

3. GENERAL ANALYSIS AND REPORTING CONVENTIONS

The following analyses and reporting conventions will be used:

- Study phase definitions:
  - The lead-in phase refers to clinical data collected from the date of first dose of study medication (visit 0, week 0) until the day before the randomization visit (visit 3, week 12).
  - The blinded treatment phase refers to clinical data collected from the date of the randomization visit (visit 3, week 12) until the day before the first observation phase visit (visit 10, week 40).
  - The observation phase refers to clinical data collected from the date of the first observation phase visit (visit 10, week 40) until the end of study visit (visit 22, week 88).
- Randomized treatment groups:
  - Abatacept treatment arm refers to the subjects who are randomized to receive weekly subcutaneous injections of abatacept (125 mg) from weeks 12 through 39 and receive subcutaneous injections of ustekinumab placebo at weeks 16 and 28.
  - Ustekinumab treatment arm refers to the subjects who are randomized to receive weekly subcutaneous injections of abatacept placebo from weeks 12 through 39 and receive subcutaneous injections of ustekinumab (45 mg or 90 mg) at weeks 16 and 28.
- Categorical variables will be summarized using counts (n) and percentages (%) and will be presented in the form "n (%)." Percentages will be rounded to one decimal place.
- Numeric variables will be summarized using n, mean, standard deviation (SD), median, minimum (min), maximum (max). The min/max will be reported at the same level of significant digits as original data. The mean and median will be reported at one more significant digit than the precision of the data, and SD will be reported at two more significant digits than the precision of the data.
- The median will be reported as the average of the two middle numbers if the dataset contains an even number of observations.
- Test statistics including *t* and *z* test statistics will be reported to two decimal places.
- *P*-values will be reported to three decimal places if greater than or equal to 0.001. If less than 0.001, the value will be reported as "<0.001." A *p*-value can be reported as "1.000" only if it is exactly 1.000 without rounding. A *p*-value can be reported as "0.000" only if it is exactly 0.000 without rounding.

If departures from these general conventions are present in the specific evaluations section of this SAP, then those conventions will take precedence over these general conventions.

4. ANALYSIS SAMPLES

Intent to treat (ITT) sample will be defined as all participants who are found to be eligible after the initial 12 week lead-in period and undergo random assignment. Any randomized subject who fails to meet any objectively measured entry criterion assessed prior to randomization will be excluded per the guidelines described for the "full analysis set" in ICH E9 Section V.5.2.1.

Per protocol (PP) sample will be defined as treated participants in the ITT sample who receive study regimen, as defined below, until 39 weeks:

- Receive 2 injections of ustekinumab during lead-in period.
- Receive 2 injections of ustekinumab or ustekinumab placebo during the treatment period.
- Receive at least 24 of the 28 abatacept or abatacept placebo injections prescribed.

A blinded data review panel will evaluate deviations from the protocol including, for example, violations of entry criteria, departures from assigned treatment regimen, or administration of study procedures outside the specified visit windows. The panel may exclude subjects with serious protocol deviations from the PPS sample.

Safety sample (SS) will be defined as all participants who receive at least one dose of study treatment after enrollment into the study.

5. STUDY SUBJECTS

5.1 Disposition of Subjects

The disposition of all enrolled subjects will be summarized in tables and listed.

The numbers and percentages of subjects enrolled, randomized, in each analysis sample, and completing the lead-in phase, blinded treatment phase, and the observation phase, as well as reasons for early termination from the study will be presented by treatment group within each randomization stratum and overall. For subjects discontinuing study treatment early, the reasons for discontinuing study treatment early will also be presented. A CONSORT diagram will be prepared to graphically display the disposition changes by treatment group throughout the phases of the study. Disposition of all enrolled subjects will be listed by randomization stratum, treatment group and subject.

5.2 Demographic and Other Baseline Characteristics

Summary descriptive statistics for baseline and demographic characteristics will be reported by treatment group within each randomization stratum (PASI score at week 0: 12-20 and >20) and overall for enrolled and randomized subjects and for the ITT and PP samples. Characteristics to be summarized include age, race, ethnicity, sex, body weight at screening, height, baseline Psoriasis Area and Severity Index (PASI), baseline Physician's Global Assessment (PGA) and baseline Dermatology Life Quality Index (DLQI). Demographic and baseline characteristic data for all enrolled subjects will also be presented in a data listing by randomization stratum, treatment group and subject.

6. STUDY OPERATIONS

6.1 Protocol Deviations

The number of major protocol deviations, the number of each type of deviation, the number of site level deviations, the number of consented subjects with at least one deviation, the number of deviations leading to termination of a subject from the study, and the number of deviations resulting in an AE will be summarized over all sites.

Protocol deviations will be sorted by site and subject, and listed with information such as type of deviation, date of occurrence, details of the deviation, steps taken to address the deviation, whether the subject was terminated from the study, whether the deviation resulted in an adverse event, who identified the deviation, and whether the deviation met IRB reporting requirements.

6.2 Treatment Adherence

Treatment adherence will be summarized for the ITT sample in the lead-in phase and for the ITT sample by treatment group within each randomization stratum and overall in the blinded treatment phase. The total number of injections received by each subject will be calculated along with the percent of the expected injections received for each study medication. Study drug administration data will be listed by randomization stratum, treatment group and subject.

6.2.1 Ustekinumab

During the lead-in phase of the study, open-label ustekinumab will be administered by site personnel as a subcutaneous injection on week 0 and week 4. Subjects who weigh ≤ 100 kg at screening will be administered 45 mg of ustekinumab and subjects who weigh > 100 kg at screening will be administered 90 mg of ustekinumab.

During the blinded treatment phase, ustekinumab or ustekinumab placebo will be administered by site personnel as a subcutaneous injection on week 16 and week 28. Subjects who weigh ≤ 100 kg at screening will be administered 45 mg of ustekinumab or 0.5 ml of ustekinumab placebo. Subjects who weigh > 100 kg at screening will be administered 90 mg of ustekinumab or 1.0 ml of ustekinumab placebo.

The date and time of injection, dose administered and information on missed injections will be recorded by site staff at the time of injection.

6.2.2 Abatacept

During the blinded treatment phase, subjects will be instructed to self-administer weekly subcutaneous injections of abatacept (125 mg in 1 mL) or abatacept placebo (1 mL) for weeks 12 through 39 via pre-filled single use syringes. The date of injection and information on missed injections will be recorded by the subject on a diary at the time of injection.

7. ENDPOINT EVALUATION

7.1 Overview of Efficacy Analysis Methods

7.1.1 Multicenter Studies

Study subjects were recruited from 10 study sites. Study data will be analyzed as a whole. Because randomization was not stratified by clinical site, it will not be included as a covariate in the analyses.

7.1.2 Assessment Time Windows

Visit 0 (week 0) must occur within 28 days of visit –1 (screening visit). All other scheduled study visits for visit 0 (week 0) through visit 22 (week 88) should occur within ±5 days of the expected visit date.

Randomized subjects who discontinue from the study, as described in section 5.3 of the protocol, will be asked to complete the early discontinuation visit (visit 22) assessments. Randomized subjects will be asked to return for Follow-up Visit D 12 weeks after completing the early discontinuation visit or 12 weeks after completing the study at week 88. All assessments listed at Visit D should be completed. Visit D should occur within ±7 days of the expected visit date.

Data collected outside of the assessment time windows will not be excluded from the analyses.

Unscheduled visits may also be performed throughout the study to document any new symptoms. The results captured in any unscheduled visit will not be included in summary displays by visit, but will be displayed in any subject level plots over time and in listings. Early discontinuation visits are recorded in the database as an unscheduled visit but will be handled differently than other unscheduled visits. Early discontinuation visit evaluations will be applied to the closest scheduled missed visit for summary displays but will be labeled as an early discontinuation visit in listings.

7.1.3 Summary of Primary and Secondary Efficacy Analysis Methods

Table 7-2 Table of Endpoints and Analysis Methods

Endpoint	Analysis Method
**Primary Endpoint**	
Proportion of subjects who experience a psoriasis relapse between weeks 12 and 88 (ITT sample)	Analysis: Logistic regression model on treatment<br>Covariates:<br>• PASI at week 0 (12-20, >20)<br>• Duration of disease prior to screening
**Sensitivity Analyses for Primary Endpoint**	
Primary endpoint (PP sample)	Same as primary
Primary endpoint (ITT sample; Drop outs = no relapse)	Same as primary

Primary endpoint (ITT sample; Drop outs = missing relapse status)	Same as primary
**Secondary Endpoints**	
Proportion of subjects who experience a psoriasis relapse between weeks 12 and 40 (ITT sample)	Same as primary
Proportion of subjects who experience a psoriasis relapse between weeks 12 and 40 (ITT sample; Drop outs = no relapse)	Same as primary
Proportion of subjects who experience a psoriasis relapse between weeks 12 and 40 (ITT sample; Drop outs = missing relapse status)	Same as primary
Proportion of subjects who experience a psoriasis relapse between weeks 12 and 40 (PP sample)	Same as primary
Proportion of subjects who experience a psoriasis relapse between weeks 28 and 88 (ITT sample)	Same as primary
Proportion of subjects who experience a psoriasis relapse between weeks 28 and 88 (ITT sample; Drop outs = no relapse)	Same as primary
Proportion of subjects who experience a psoriasis relapse between weeks 28 and 88 (ITT sample; Drop outs = missing relapse status)	Same as primary
Proportion of subjects who experience a psoriasis relapse between weeks 28 and 88 (PP sample)	Same as primary
Proportion of subjects who experience a psoriasis relapse between weeks 40 and 88 (ITT sample)	Same as primary
Proportion of subjects who experience a psoriasis relapse between weeks 40 and 88 (ITT sample; Drop outs = no relapse)	Same as primary
Proportion of subjects who experience a psoriasis relapse between weeks 40 and 88 (ITT sample; Drop outs = missing relapse status)	Same as primary
Proportion of subjects who experience a psoriasis relapse between weeks 40 and 88 (PP sample)	Same as primary
Length of time until psoriasis relapse between weeks 12 and 40 (ITT sample)	Analysis: Grouped survival analysis model on treatment<br>Covariates:<br>• PASI at week 0 (12-20, >20)<br>• Duration of disease prior to screening

Length of time until psoriasis relapse between weeks 12 and 40 (ITT sample; Drop outs = censored at the time of drop-out)	Analysis: Grouped survival analysis model on treatment<br>Covariates:<br>• PASI at week 0 (12-20, >20)<br>• Duration of disease prior to screening
Length of time until psoriasis relapse between weeks 12 and 40 (PP sample)	Analysis: Grouped survival analysis model on treatment<br>Covariates:<br>• PASI at week 0 (12-20, >20)<br>• Duration of disease prior to screening
Length of time until psoriasis relapse between weeks 12 and 88 (ITT sample)	Analysis: Grouped survival analysis model on treatment<br>Covariates:<br>• PASI at week 0 (12-20, >20)<br>• Duration of disease prior to screening
Length of time until psoriasis relapse between weeks 12 and 88 (ITT sample; Drop outs = censored at the time of drop-out)	Analysis: Grouped survival analysis model on treatment<br>Covariates:<br>• PASI at week 0 (12-20, >20)<br>• Duration of disease prior to screening
Length of time until psoriasis relapse between weeks 12 and 88 (PP sample)	Analysis: Grouped survival analysis model on treatment<br>Covariates:<br>• PASI at week 0 (12-20, >20)<br>• Duration of disease prior to screening
Proportion of participants who are classified as cleared or minimal in the PGA average score at week 40 (ITT sample)	Same as primary
Proportion of participants who are classified as cleared or minimal in the PGA average score at week 40 (PP sample)	Same as primary
Proportion of participants who are classified as cleared or minimal in the PGA average score at week 88 (ITT sample)	Same as primary
Proportion of participants who are classified as cleared or minimal in the PGA average score at week 88 (PP sample)	Same as primary
Change in DLQI from week 12 to week 40 (ITT sample)	Analysis: ANCOVA on treatment<br>Covariates:<br>• DLQI at week 0<br>• PASI at week 0 (12-20, >20)<br>• Duration of disease prior to screening
Change in DLQI from week 12 to week 40 (PP sample)	Analysis: ANCOVA on treatment<br>Covariates:<br>• DLQI at week 0<br>• PASI at week 0 (12-20, >20)<br>• Duration of disease prior to screening

Change in DLQI from week 12 to week 88 (ITT sample)	Analysis: ANCOVA on treatment<br>Covariates:<br>• DLQI at week 0<br>• PASI at week 0 (12-20, >20)<br>• Duration of disease prior to screening
Change in DLQI from week 12 to week 88 (PP sample)	Analysis: ANCOVA on treatment<br>Covariates:<br>• DLQI at week 0<br>• PASI at week 0 (12-20, >20)<br>• Duration of disease prior to screening

7.2 Primary Endpoint

7.2.1 Computation of the Primary Endpoint

The primary endpoint is the proportion of subjects who experience a psoriasis relapse at any evaluation between week 12 and week 88.

Psoriasis relapse is defined as loss of ≥ 50% of the initial Psoriasis Area and Severity Index (PASI) improvement measured at week 12. The threshold for when a subject's psoriasis worsens to the point of meeting the criterion for a relapse is calculated as:

Relapse PASI criterion = Week 12 PASI + [(Week 0 PASI –Week 12 PASI)/2]

where: Week 0 PASI is the subject's PASI score at the week 0 visit

Week 12 PASI is the subject's PASI score as the week 12 visit

Any PASI score assessed after the week 12 visit that is ≥ relapse PASI criterion qualifies as a psoriasis relapse for this study.

PASI is assessed at every visit except follow-up visit D.

7.2.2 Primary Analysis of the Primary Endpoint

The primary analysis for the primary endpoint will be performed on the ITT sample and is designed to test the following hypotheses:

- Null hypothesis: The proportion of subjects who experience a psoriasis relapse at any evaluation between week 12 and week 88 is equivalent for the abatacept and ustekinumab treatment arms
- Alternative hypothesis: The proportion of subjects who experience a psoriasis relapse at any evaluation between week 12 and week 88 differs between the abatacept and ustekinumab treatment arms

The proportions of subjects will be compared between the two treatment arms by a logistic regression model with the participant's relapse status (relapse or no relapse) as the dependent variable and treatment as the independent variable. The randomization stratum (PASI score at week 0: 12-20 or >20) will be used as a covariate in the logistic regression model due to the inclusion of this variable as a stratification factor in the randomization. The duration of disease prior to screening (i.e. time from onset of

psoriasis to screening visit), centered about its median, will be used as an additional covariate in the logistic regression model.

For the primary analysis of the primary endpoint, subjects who terminate early from the study (drop-outs) will be treated as having experienced a psoriasis relapse.

7.2.3 Sensitivity Analyses of the Primary Endpoint

The primary analysis method described above will be repeated using the PP sample, if different from the ITT sample.

Additional sensitivity analyses will be performed on the ITT sample to assess the effect of the drop-out assumptions on the analysis results by repeating the primary endpoint analysis but with different assumptions applied to the drop-out subjects. Subjects who terminate early from the study due to meeting the psoriasis relapse criterion or due to worsening psoriasis will be treated as having experienced a psoriasis relapse in all sensitivity analyses. Subjects who terminate early from the study for any other reason will be included in the following sensitivity analysis assumptions:

- Drop-out subjects assumed to have not experienced a psoriasis relapse
- Drop-out subjects treated as having a missing psoriasis relapse status

The primary endpoint analysis will be repeated, but with each of the above sensitivity analysis assumptions applied to the drop-out subjects. .

7.3 Secondary Endpoints and Analyses

The secondary analyses will support the primary analysis by providing a deeper understanding of events. P-values for all inferential analyses will be presented as a description of strength of evidence of relationships rather than tests of hypotheses. As such, no corrections for multiplicity are planned. All secondary endpoint analyses will be performed on the ITT and PP samples for subjects with available data unless otherwise stated.

Listings will be prepared for all efficacy assessments with the subjects in the ITT sample. All efficacy listings will be sorted in order of randomization stratum, treatment group, subject identifier, and time of assessment (e.g., visit, time, and/or event).

7.3.1 Psoriasis Relapse and PASI

In addition to the primary and sensitivity analyses for the primary endpoint, the following analyses on psoriasis relapse and PASI are planned:

- PASI scores will be summarized over time by treatment group within each randomization stratum and overall.
- PASI scores will be plotted over time by treatment group within each randomization stratum in spaghetti plots showing individual subject scores and in plots showing the means and standard errors.
- Change in PASI score from week 12 (randomization) to each post-randomization visit will be summarized by treatment group within each randomization stratum and overall.

- The proportion of participants who experience a psoriasis disease relapse at any evaluation between week 12 and week 40 will be analyzed using a logistic regression model similar to the one used in the primary endpoint analysis. The analysis will be performed on the ITT and PP samples and will be repeated on the following drop-out assumptions:
    - Drop-out subjects assumed to have experienced a psoriasis relapse (ITT and PP samples)
    - Drop-out subjects assumed to have not experienced a psoriasis relapse (ITT sample only)
    - Drop-out subjects treated as having a missing psoriasis relapse status (ITT sample only)
- The proportion of participants who experience a psoriasis disease relapse at any evaluation between week 28 and week 88 will be analyzed using a logistic regression model similar to the one used in the primary endpoint analysis. Due to inherent bias with respect to the time intervals and treatment administration periods, caution will be exercised in interpreting the results of these analyses. There may be inherent bias in the evaluation between week 28 and week 88 since the last dose of ustekinumab or ustekinumab placebo will be administered at week 28 but the abatacept or abatacept placebo doses continue until week 39. The analysis will be performed on subjects in the ITT and PP samples with at least one PASI score evaluation between week 28 and week 88, and will be repeated on the following drop-out assumptions:
    - Drop-out subjects assumed to have experienced a psoriasis relapse (ITT and PP samples)
    - Drop-out subjects assumed to have not experienced a psoriasis relapse (ITT sample only)
    - Drop-out subjects treated as having a missing psoriasis relapse status (ITT sample only)
- The proportion of participants who experience a psoriasis disease relapse at any evaluation between week 40 and week 88 will be analyzed using a logistic regression model similar to the one used in the primary endpoint analysis. Due to inherent bias with respect to the time intervals and treatment administration periods, caution will be exercised in interpreting the results of these analyses. There may be inherent bias in the evaluation between week 40 and week 88 since the last dose of abatacept or abatacept placebo will be administered at week 39, 11 weeks after the last dose of ustekinumab or ustekinumab placebo was administered. The analysis will be performed on subjects in the ITT and PP samples with at least one PASI score evaluation between week 40 and week 88, and will be repeated on the following drop-out assumptions:
    - Drop-out subjects assumed to have experienced a psoriasis relapse (ITT and PP samples)

  - Drop-out subjects assumed to have not experienced a psoriasis relapse (ITT sample only)
  - Drop-out subjects treated as having a missing psoriasis relapse status (ITT sample only)
- The length of time until psoriasis relapse in the intervals from week 12 to 40 and week 12 to 88 will be analyzed using a grouped survival analysis model (Allison, 2010). The logistic regression model will use the participant's relapse status (relapse or no relapse) as the dependent variable and treatment as the independent variable. Each post-randomization study visit from week 16 through week 88 up to the interval when relapse occurred (if any) will be included as a separate observation in the dataset. Similar to the primary analysis, randomization stratum and duration of disease prior to screening will also be included in the model as covariates. The predicted survival curves for the 2 treatment sequences will be derived from unadjusted survival estimates by treatment group within each stratum. The analysis will be performed on the ITT and PP samples and will be repeated on the following drop-out assumptions:
  - Drop-out subjects assumed to have experienced a psoriasis relapse (ITT and PP samples)
  - Drop-out subjects treated as being censored at the time of drop-out (ITT sample only)
-

7.3.2 Physician's Global Assessment (PGA)

The Physician's Global Assessment (PGA) will be assessed at weeks 0, 12, 24, 40, 56, 72 and 88. The PGA assesses the severity of the subject's psoriasis in 3 components: induration, erythema and scaling. Each component is given a score from 0 to 5 based on the majority of the subject's psoriasis lesions. PGA component scores are classified as follows:

0 = Clear
1 = Minimal
2 = Mild
3 = Moderate
4 = Marked
5 = Severe

The 3 component scores are averaged to obtain the PGA average score. A PGA average score < 1.5 will be classified as clear/minimal. The following analyses are planned:

- PGA scores will be summarized and plotted over time by treatment group within each randomization stratum and overall.
- The proportions of participants who are classified as cleared or minimal in the PGA score at week 40 and week 88 will be analyzed using a logistic regression model similar to the one used in the primary endpoint analysis. The participant's PGA score category (clear/minimal vs. mild or worse) will be the dependent

variable in the model and treatment group will be the independent variable. Covariate variables in the model will be the randomization stratum and duration of disease prior to screening.

7.3.3 Dermatology Life Quality Index (DLQI)

The Dermatology Life Quality Index (DLQI) will be assessed at weeks 0, 12, 24, 40, 56, 72 and 88. The DLQI is a questionnaire that assesses a subject's quality of life with respect to skin conditions in the areas of symptoms and feelings, daily activities, leisure, work and school, personal relationships and treatment. Responses to each question range from 'Not at all' (score = 0) to 'Very much' (score = 3). The DLQI score is the sum of the scores for all 10 questions and has the following interpretation:

0-1 = No effect at all on patient's life

2-5 = Small effect on patient's life

6-10 = Moderate effect on patient's life

11-20 = Very large effect on patient's life

21-30 = Extremely large effect on patient's life

The following analyses are planned:

- DLQI scores will be summarized and plotted over time by treatment group within each randomization stratum.
- Change in DLQI score from week 12 (randomization) to each post-randomization visit will be summarized by treatment group within each randomization stratum and overall.
- Change in DLQI score from week 12 (randomization) to week 40 will be analyzed using an analysis of covariance model (ANCOVA) with the change in DLQI score as the dependent variable, treatment as the independent variable, and DLQI score at week 0, randomization stratum and duration of disease prior to screening as covariates. This analysis will be repeated for the change in DLQI score from week 12 (randomization) to week 88.

8. SAFETY EVALUATION

8.1 Overview of Safety Analysis Methods

All safety analyses will be carried out using the safety sample defined in Section 4 unless otherwise noted. Missing safety information will not be imputed. These analyses will not be stratified by site.

Listings will be prepared for all safety assessments with the subjects in the safety sample. All safety listings will be sorted in order of randomization stratum, treatment group, subject identifier, and time of assessment (e.g., visit, time, and/or event).

8.2 Adverse Events

All AEs will be classified by system organ class and preferred term, according to the Medical Dictionary for Regulatory Activities [MedDRA] version 17.0. The severity of AEs will be classified using the National Cancer Institute’s (NCI’s) Common Toxicity Criteria for Adverse Events (CTCAE) version 4.03. Each AE is entered on the electronic case report form (eCRF) once at the highest severity. AEs will be collected from screening through study termination. Treatment-emergent AEs will be identified as those with an onset date on or after the first dose of study medication as well as those with onset before first dose but that continued and worsened in severity after first dose. If the start of the AE in relation to the start of study medication cannot be established (e.g., the start date for the AE is missing), then the AE will be considered treatment-emergent. All data tabulations will be of only treatment-emergent events while non-treatment-emergent AEs will be included in the listings.

All AE summaries will be reported separately for the lead-in phase and post-randomization phase. Summaries for the lead-in phase will only be reported overall, since all subjects received open-label ustekinumab during this study phase. The post-randomization phase will consist of treatment-emergent AEs occurring during the blinded treatment phase and the observation phase.

An overall summary table will report the number and percentage of AEs and the number and percentage of subjects experiencing an AE by treatment group within each stratum and overall.

- All AEs
- AEs indicated as serious
- AEs with an outcome of death
- AEs indicated as study specific events (AESIs)
- AEs by severity
- AEs that lead to study drug discontinuation
- AEs by relatedness to each study drug

Additional AE summary tables will report the number and event rate per person-year of AEs, event rate ratios, the number and percentage of subjects experiencing an AE,

relative risks and p-values for treatment group comparisons. Separate summaries will be prepared for each randomization stratum and overall. The relative risks (and rate ratios) will be calculated as Abatacept treatment group percent (or event rate) divided by Ustekinumab treatment group percent (or event rate). Fisher's exact test will be used to compare the treatment groups for the proportions of subjects experiencing the events. Treatment group comparisons for the numbers of distinct episodes will be derived from a Poisson regression model with the number of events as the dependent variable, and treatment group as the dependent variable. The *ln* link together with the Poisson distribution will be applied; the *ln* of the amount of follow-up will be included as an offset to account for differential amount of follow-up contributed by the different subjects. The amount of person-year follow-up time for each subject will be calculated as:

$$person - year\ follow - up = \frac{last\ day\ in\ study\ phase - first\ day\ in\ study\ phase}{365.25}$$

The summary tables will contain the following categories of events:

- All AEs
- AEs indicated as serious
- AEs with an outcome of death
- AEs with severity Grade 3 or higher
- AEs indicated as study specific events (AESIs)
- AEs that lead to study drug discontinuation
- AEs that were reported as being related to a study drug
- AEs by system organ class and preferred term, where at least 5 AEs occurred in either treatment group

In addition, AEs classified by MedDRA system organ class and preferred term will be summarized by treatment group within each randomization stratum and overall for each of the following:

- All AEs
- AEs by severity
- AEs by relationship to study drug

Summary tables will present the number of events and event rate per person-year as well as the number and percentage of subjects experiencing the events. If a subject experiences the same AE on multiple occasions, the event will be counted once for each occurrence when reporting the number of AEs. When reporting the number of subjects experiencing the events, a subject will only be counted once if they experience an event within the particular SOC or preferred term. Percentages will be based on the number of subjects in the safety population.

Separate data listings will be provided for treatment-related AEs and AEs leading to study drug discontinuation.

8.3 Deaths and Serious Adverse Events

Serious adverse events (SAEs) will be listed by randomization stratum, treatment group and subject. If any subjects die, a listing will be created and will include the primary cause of death and time to death.

8.4 Pregnancies

Pregnancies that occurred in enrolled subjects during study participation will be listed by treatment group and subject. The listing will include date of last menstrual period, expected date of delivery, and date and type of pregnancy outcome.

8.5 Clinical Laboratory Evaluation

Clinical laboratory measurements include serum chemistry, urinalysis, and hematology. Results will be converted to standardized units where possible.

The following analyses are planned:

- Laboratory data will be plotted by treatment group within randomization stratum to show patterns over time. Summary statistics including 25th percentile, median, and 75th percentile will be plotted for each visit by treatment group and randomization stratum. Lines connecting individual subject results from subjects with grade 2 or higher values will be overlaid on each figure. For lab results that are not gradable, results from subjects with values outside of 2 *upper limit of normal or 0.5*lower limit of normal will be overlaid. Tests with qualitative results (such as "present" or "positive") will not be plotted.
- Change in laboratory results from baseline at each laboratory collection visit will be summarized by treatment group within each randomization stratum and overall.

8.6 Vital Signs, Physical Findings, and Other Observations Related to Safety

8.6.1 Vital Signs

Vital sign results will be plotted by treatment group within each randomization stratum to show patterns over time. Summary statistics including 25th percentile, median, and 75th percentile will be plotted for each visit. Lines connecting individual subject results from subjects with grade 2 or higher values will be overlaid on each figure.

8.6.2 Physical Examinations

Physical examination results of normal, abnormal, and not done will be summarized as frequencies and percentages by body system and visit by treatment group within each randomization stratum and overall. Body systems classified as abnormal in the physical exams will be listed.

9. OTHER ANALYSES

9.1 Psoriatic Arthritis Screening and Evaluation (PASE)

The Psoriatic Arthritis Screening and Evaluation (PASE) will be assessed at every visit except follow-up visit D. The PASE assesses the severity of the subject's psoriatic arthritis in the areas of symptoms and function. Responses to each question range from 'Strongly Disagree' (score = 1) to 'Strongly Agree' (score = 5). Higher PASE scores indicate higher severity of psoriatic arthritis. The PASE score is the sum of the scores for all 15 questions.

The following analyses are planned in the ITT sample:

- PASE scores will be summarized and plotted over time by treatment group within each randomization stratum and overall.
- Change in PASE score from week 12 (randomization) to week 88 will be analyzed using an analysis of covariance model (ANCOVA) with the PASE score as the dependent variable, treatment as the independent variable, and PASE score at week 0, randomization stratum and disease duration prior to screening as covariates.
- Change in PASE score from week 12 (randomization) to each post-randomization visit will be summarized by treatment group within each randomization stratum and overall.
- PASE scores will be listed by randomization stratum, treatment group, subject and visit.

9.2 Use of Medications

Medications will be coded according to the World Health Organization (WHO) Drug Dictionary (version V2014.01). The number and percentage of subjects receiving prior and concomitant medications will be presented overall and by medication class. When reporting the number of subjects receiving the medication, a subject will only be counted once if they ever received the medication within the medication class. Percentages will be based on the number of subjects in the safety sample. Concomitant medications will be presented in a data listing and prohibited medications will be flagged. Medications that were started prior to week 0 will be denoted with an asterisk.

9.3 Medical History

Body systems classified as abnormal in the medical history eCRF will be listed by randomization stratum, treatment group, subject and body system.

9.4 Assessments Collected at Follow-up Visit D

For randomized subjects, a limited number of safety assessments will be performed 12 weeks after the subject terminates the study (Follow-up Visit D).

9.4.1 Physician's Global Assessment (PGA)

The PGA scores collected at Follow-up Visit D will be summarized by treatment group and overall. The PGA scores at this visit will be included in the PGA subject listing specified in the secondary endpoint analyses in section 7.3.2.

9.4.2 Adverse Events

The adverse events collected at Follow-up Visit D will be summarized by MedDRA system organ class and preferred term for each treatment group and overall. The adverse events collected at this visit will be included in the adverse event listings, as applicable, specified in the safety analyses in sections 8.2 and 8.3.

9.4.3 Use of Medications

The medication usage collected at Follow-up Visit D will be summarized by medication class and overall. The medications collected at this visit will be included in the medication listing specified in the planned analyses in section 9.2.

10. INTERIM ANALYSES AND DATA MONITORING

The progress of the study will be monitored by the Autoimmune Data and Safety Monitoring Board (DSMB). The Autoimmune DSMB will be chartered to review safety data and to make recommendations regarding continuation, termination, or modification of the study. The DSMB will formally review the safety data at least yearly. The discontinuation of study treatment will also be periodically reported to the DSMB.

In addition, safety data will be reviewed by the DSMB when an event occurs that is of sufficient concern to the National Institute of Allergy and Infectious Diseases (NIAID) medical monitor or protocol chair to warrant review, or when an event occurs that could contribute to a predefined stopping rule specified in the protocol.

11. CHANGES TO THE ANALYSES PLANNED IN THE PROTOCOL

11.1 Psoriasis Relapse and PASI secondary endpoint analysis

Protocol section 9.2.3 Secondary Endpoints plans for psoriasis relapse analyses with the assumption that drop-out subjects are assumed to have experienced a psoriasis relapse. We have added additional sensitivity analyses similar to the sensitivity analyses planned for the primary endpoint analysis in which the analyses are repeated with drop-out subjects assumed to have not experienced a psoriasis relapse at the time of study withdrawal, and with drop-out subjects as having a missing psoriasis relapse status.

11.2 Dermatology Life Quality Index (DLQI) secondary endpoint analysis

Protocol section 9.2.3 Secondary Endpoints plans for the following analysis of the change in Dermatology Life Quality Index (DLQI):

The change in Dermatology Life Quality Index (DLQI) at week 40 and at week 88, as defined in section 3.3.2, will be analyzed for the ITT and PP samples using an analysis of covariance model (ANCOVA) with the change in DLQI score as the dependent variable, treatment as the independent variable, and baseline DLQI score, screening PASI score and disease duration at screening as covariates.

In section 7.3.3 Dermatology Life Quality Index (DLQI) of this Statistical Analysis Plan, we have changed the PASI score covariate for the planned ANCOVA model to be more consistent with the covariates used in the other secondary endpoint analyses. The covariates for the ANCOVA model now include DLQI score at week 0, randomization stratum (PASI score at week 0: 12-20 or >20) and duration of disease prior to screening.

12. REFERENCES

Allison, P. D. (2010). Analysis of Tied of Discrete Data with PROC LOGISTIC. In *Survival Analysis Using SAS: A Practical Guide* (pp. 235-256). Cary, NC: SAS Institute, Inc.

13. APPENDICES

13.1 Study Flow Chart

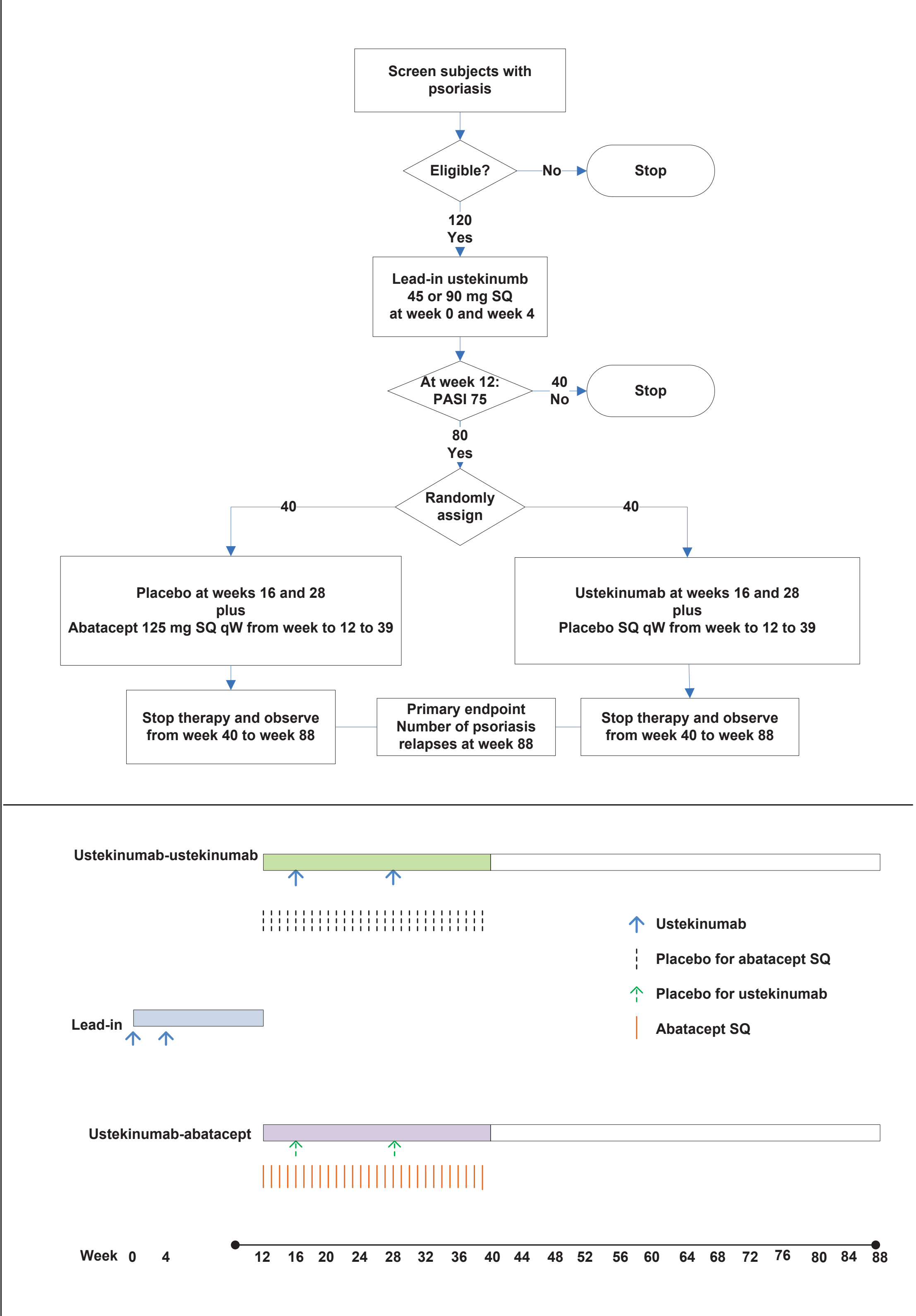

13.2 Schedule of Events

**Study Week**		**0**	**4**	**8**	**12**	**16**	**20**	**24**	**28**	**32**	**36**	**39**
**Visit Number**	**-1**	**0**	**1**	**2**	**3**	**4**	**5**	**6**	**7**	**8**	**9**	**---**
**GENERAL ASSESSMENTS**												
Informed Consent	x											
Randomization					x							
Demographics (age and ethnicity)	x											
Medical History	x											
Psoriasis History	x											
Comprehensive Physical Exam	x											
Brief Physical Exam		x	x	x	x	x	x	x	x	x	x	
Adverse Events	x	x	x	x	x	x	x	x	x	x	x	
Concomitant Medications	x	x	x	x	x	x	x	x	x	x	x	
Vital Signs	x	x	x	x	x	x	x	x	x	x	x	
**CLINICAL LABORATORY ASSESSMENTS**												
Serum Chemistry (AST, ALT, bilirubin, alkaline phosphatase, random glucose)	x				x			x				
Hematology	x				x			x	x			
Serum Creatinine	x				x			x				
RPR	x											
Serum HCG	x											
QuantiFERON -TB Gold	x											
Urinalysis	x											
STAT Urine HCG		x	x	x	x	x	x	x	x	x	x	
HIV	x											
Hepatitis B & C	x											
Chest X-ray	x											

**Study Week**		**0**	**4**	**8**	**12**	**16**	**20**	**24**	**28**	**32**	**36**	**39**
**Visit Number**	**-1**	**0**	**1**	**2**	**3**	**4**	**5**	**6**	**7**	**8**	**9**	**---**
**PSORIASIS ASSESSMENTS**												
PASI[1]	x	x	x	x	x	x	x	x	x	x	x	
PGA		x			x			x				
DLQI		x			x			x				
PASE	x	x	x	x	x	x	x	x	x	x	x	
Skin Photography[2]		x			x							
**STUDY DRUG ADMINISTRATION**												
Ustekinumab		x	x									
Ustekinumab/ Ustekinumab Placebo						x			x			
Abatacept/Abatacept Placebo[3]					x	x	x	x	x	x	x	x
**MECHANISTIC LABORATORY ASSESSMENTS**												
Lesional Punch Skin Biopsy		x			x			x[4]				
Non-lesional Punch Skin Biopsy		x										
Plasma/Serum Assays		x			x			x				
PBMC Assays		x			x			x				
RNA Assays		x			x			x				

[1] Each PASI assessment will include the Body Surface Area psoriasis involvement.

[2] Skin Photography is optional.

[3] SQ Abatacept is given weekly from week 12-39.

[4] Lesional Punch Skin Biopsy is optional at week 24 visit.

Study Week	40	44	48	52	56	60	64	68	72	76	80	84	88		
**Visit Number**	**10**	**11**	**12**	**13**	**14**	**15**	**16**	**17**	**18**	**19**	**20**	**21**	**22**	**D[5]**	**U[6]**
**GENERAL ASSESSMENTS**															
Informed Consent															
Randomization															
Demographics (age and ethnicity)															
Medical History															
Psoriasis History															
Comprehensive Physical Exam															
Brief Physical Exam	x	x	x	x	x	x	x	x	x	x	x	x	x		x
Adverse Events	x	x	x	x	x	x	x	x	x	x	x	x	x	x	x
Concomitant Medications	x	x	x	x	x	x	x	x	x	x	x	x	x	x	x
Vital Signs	x	x	x	x	x	x	x	x	x	x	x	x	x		x
**CLINICAL LABORATORY ASSESSMENTS**															
Serum Chemistry (AST, ALT, bilirubin, alkaline phosphatase, random glucose)	x								x						x
Hematology	x				x				x				x		x
Serum Creatinine	x														
RPR															
Serum HCG															
QuantiFERON -TB Gold															
Urinalysis															
STAT Urine HCG				x									x		x
HIV															

[5] The follow-up visit D will occur in randomized participants 12 weeks after discontinuation of study treatment or study observation (see section 5.3). Visit D will occur 12 weeks after Visit 22 for participants who complete Visit 22.

[6] Unscheduled visits may be performed to document any symptoms or to document a psoriasis relapse during the study (see section 6.1.2).

**Study Week**	**40**	**44**	**48**	**52**	**56**	**60**	**64**	**68**	**72**	**76**	**80**	**84**	**88**		
**Visit Number**	**10**	**11**	**12**	**13**	**14**	**15**	**16**	**17**	**18**	**19**	**20**	**21**	**22**	**D[5]**	**U[6]**
Hepatitis B & C															
Chest X-ray															
**PSORIASIS ASSESSMENTS**															
PASI[1]	x	x	x	x	x	x	x	x	x	x	x	x	x		x
PGA	x				x				x				x	x	
DLQI	x				x				x				x		
PASE	x	x	x	x	x	x	x	x	x	x	x	x	x		x
Skin Photography	x												x		
**STUDY DRUG ADMNISTRATION**															
Ustekinumab															
Ustekinumab/ Ustekinumab Placebo															
Abatacept/Abatacept Placebo[3]															
**MECHANISTIC LABORATORY ASSESSMENTS**															
Lesional Punch Skin Biopsy	x												x		
Non-lesional Punch Skin Biopsy	x												x[7]		
Plasma/Serum Assays	x				x				x				x		
PBMC Assays	x				x				x				x		
RNA Assays	x				x				x				x		

[7] The week 88 non-lesional biopsy is considered optional.
