# Supplementary material for: Effect of Costimulatory Blockade With Abatacept After Ustekinumab Withdrawal in Patients With Moderate to Severe Plaque Psoriasis: The PAUSE Randomized Clinical Trial
Source: JAMA Dermatol. 2021 Oct 13;157(11):1–11. doi: 10.1001/jamadermatol.2021.3492 (PMC8515260; doi:10.1001/jamadermatol.2021.3492)
Supplement: Supplement 2. — eFigure 1. Study Design eTable 1. Demographic and Clinical Characteristics eTable 2. Summary of Treatment Emergent Adverse Events after Randomization (Safety Analysis Set) eTable 3. Summary of Preferred Terms for Serious Adverse Events in the Lead‐In Phase (Safety Analysis Set) eTable 4. Summary of Preferred Terms for Serious Adverse Events in the Post‐Randomization Phase (Safety Analysis Set) eFigure 2. Time to Psoriasis Relapse (ITT Population) eFigure 3. Modulation of the Disease Transcriptome (PSTR) in Skin and Relevant Cytokines in Serum in Participants Eligible for Randomization (≥PASI75) eFigure 4. Ustekinumab Modulates Targets of Abatacept in Lesional Skin eFigure 5. Abatacept Fails to Maintain Suppression of IL17A in Resolving Lesions Following Ustekinumab Withdrawal in Participants That Completed Study Treatment eFigure 6. Treatment Effect of Abatacept on Serum IL‐10 and IL‐2 in PAUSE Is Consistent With ACCLAIM eTable 5. Limits of Blank (LOB) and Lower Limit of Quantitation (LLOQ) of the MSD, LLC Multi‐plex Immunoassay [file jamadermatol-e213492-s002.pdf]

Supplementary Online Content

Harris KM, Smilek DE, Byron M, et al. Effect of costimulatory blockade with abatacept after ustekinumab withdrawal in moderate to severe plaque psoriasis: the PAUSE randomized clinical trial. *JAMA Dermatol.* Published online October 13, 2021. doi:10.1001/jamadermatol.2021.3492

eFigure 1. Study Design
eTable 1. Demographic and Clinical Characteristics
eTable 2. Summary of Treatment Emergent Adverse Events after Randomization (Safety Analysis Set)
eTable 3. Summary of Preferred Terms for Serious Adverse Events in the Lead-In Phase (Safety Analysis Set)
eTable 4. Summary of Preferred Terms for Serious Adverse Events in the Post-Randomization Phase (Safety Analysis Set)
eFigure 2. Time to Psoriasis Relapse (ITT Population)
eFigure 3. Modulation of the Disease Transcriptome (PSTR) in Skin and Relevant Cytokines in Serum in Participants Eligible for Randomization (≥PASI75)
eFigure 4. Ustekinumab Modulates Targets of Abatacept in Lesional Skin
eFigure 5. Abatacept Fails to Maintain Suppression of IL17A in Resolving Lesions Following Ustekinumab Withdrawal in Participants That Completed Study Treatment
eFigure 6. Treatment Effect of Abatacept on Serum IL-10 and IL-2 in PAUSE Is Consistent With ACCLAIM
eTable 5. Limits of Blank (LOB) and Lower Limit of Quantitation (LLOQ) of the MSD, LLC Multi-plex Immunoassay

This supplementary material has been provided by the authors to give readers additional information about their work.

© 2021 Harris KM et al. et al. *JAMA Dermatology*.

eFigure 1. Study Design

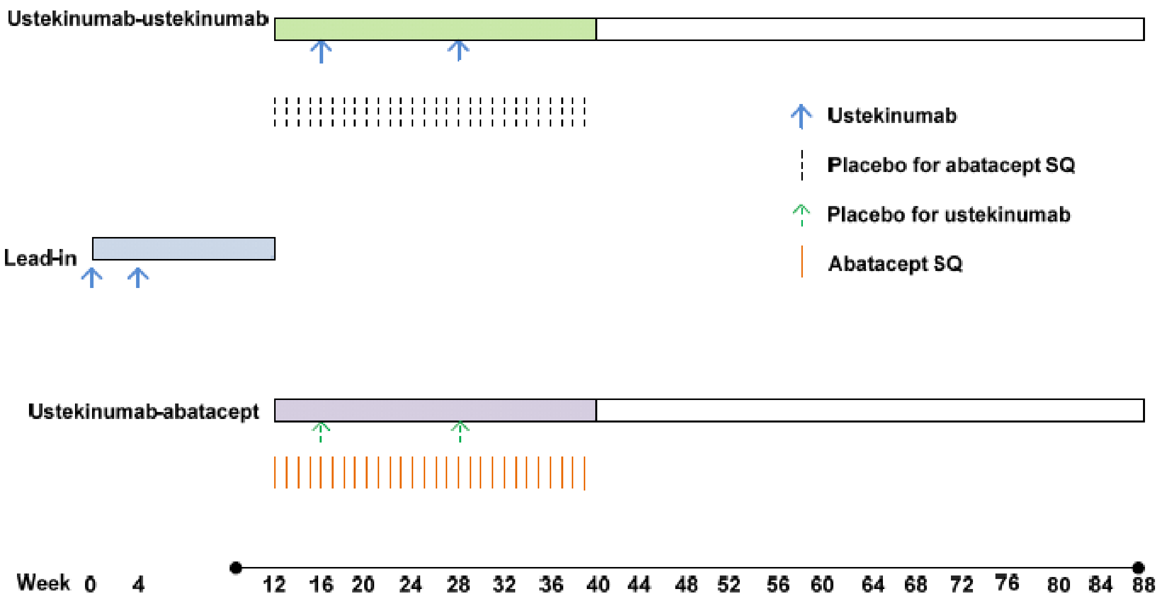

The trial consisted of a lead-in phase (weeks 0 to 12), a randomized treatment phase (weeks 12 to 40), and an observation phase (weeks 40 to 88). During the lead-in phase, participants received subcutaneous ustekinumab at weeks 0 and 4. At week 12, eligible participants were randomized to treatment with abatacept or continued ustekinumab. The ustekinumab-abatacept group received abatacept administered weekly from weeks 12 to 39, and ustekinumab placebo at weeks 16 and 28.The ustekinumab-ustekinumab group received ustekinumab at Weeks 16 and 28, and abatacept placebo weekly from weeks 12 to 39. Participants were observed for psoriasis relapse until the final study visit at week 88.

© 2021 Harris KM et al. et al. *JAMA Dermatology*.

eTable 1. Demographic and Clinical Characteristics

	No. (%) of Participants by Treatment Arm			
**Characteristic**	**Abatacept (n=45)**	**Ustekinumab (n=46)**	**Not Randomized (n=17)**	**Total (n=108)**
Age, mean (SD), y	49.4 (10.50)	44.1 (11.83)	42.8 (14.99)	46.1 (12.08)
Sex				
Male	27 (60.0)	32 (69.6)	14 (82.4)	73 (67.6)
Female	18 (40.0)	14 (30.4)	3 (17.6)	35 (32.4)
Race				
White or Caucasian	36 (80.0)	42 (91.3)	17 (100.0)	95 (88.0)
Black or African American	3 (6.7)	3 (6.5)	0	6 (5.6)
Asian	3 (6.7)	1 (2.2)	0	4 (3.7)
Other	1 (2.2)	0	0	1 (0.9)
Multiple Races	2 (4.4)	0	0	2 (1.9)
Ethnicity				
Hispanic or Latino	3 (6.7)	7 (15.2)	2 (11.8)	12 (11.1)
Not Hispanic or Latino	42 (93.3)	39 (84.8)	15 (88.2)	96 (88.9)
Weight, mean (SD), kg	94.5 (21.72)	97.8 (19.76)	104.5 (24.75)	97.5 (21.48)
Height, mean (SD), cm	170.3 (10.93)	172.6 (9.50)	174.6 (7.27)	171.9 (9.87)
BMI, mean (SD), kg/m^2	32.5 (6.73)	33.0 (7.16)	34.1 (6.78)	33.0 (6.88)
Week 0 PASI				
Mean (SD)	19.2 (8.12)	20.0 (8.24)	21.2 (7.70)	19.9 (8.06)
12–20[a]	31 (68.9)	32 (69.6)	8 (47.1)	71 (65.7)
>20[a]	14 (31.1)	14 (30.4)	9 (52.9)	37 (34.3)
Week 12 PASI				
No.	45	46	12	103
Mean (SD)	2.2 (1.81)	1.9 (2.00)	11.1 (7.77)	3.1 (4.27)

[a] Stratification factors at randomization (week 12)

© 2021 Harris KM et al. et al. *JAMA Dermatology*.

eTable 2. Summary of Treatment Emergent Adverse Events after Randomization (Safety Analysis Set)

	**No. of Participants (%)** [a]	
	**Abatacept**	**Ustekinumab**
	N = 45	N = 46
Any Adverse Events	28 (62.2)	22 (47.8)
Any Serious Events	2 (4.4)	5 (10.9)
Any Grade 3+ Events	4 (8.9)	7 (15.2)
Any Adverse Event Leading to Treatment Discontinuation	1 (2.2)	2 (4.3)
	**No. of Events (Event Rate)** [b]	
	**P-Y** [c] **= 29.6**	**P-Y = 36.9**
All Adverse Events	59 (1.995)	59 (1.601)
All Serious Events	2 (0.068)	9 (0.244)
All Grade 3+ Events	4 (0.135)	11 (0.298)
All Adverse Events Leading to Treatment Discontinuation	1 (0.034)	3 (0.081

[a] Number of participants (%) reporting at least one treatment emergent adverse event.

[b] Event rate calculated as the number of events / person-years of exposure.

[c] Person-years of exposure calculated as the total follow-up time since randomization across all participants within each treatment group.

© 2021 Harris KM et al. et al. *JAMA Dermatology*.

eTable 3. Summary of Preferred Terms for Serious Adverse Events in the Lead-In Phase (Safety Analysis Set)

	**Total Enrolled**	
	**No. of Participants (%)** [a]	**No. of Events (Event Rate)** [b]
	**N = 108**	**P-Y** [c] **= 25.5**
All Serious Adverse Events	2 (1.9)	2 (0.078)
Cervical spinal stenosis	1 (0.9)	1 (0.039)
Vertigo	1 (0.9)	1 (0.039)

[a] Number of participants (%) reporting at least one serious adverse event for the preferred term.
[b] Event rate calculated as the number of events / person-years of exposure.
[c] Person-years of exposure calculated as the total follow-up time between enrollment (Week 0) and randomization (Week 12) across all participants

© 2021 Harris KM et al. et al. *JAMA Dermatology*.

eTable 4. Summary of Preferred Terms for Serious Adverse Events in the Post-Randomization Phase (Safety Analysis Set)

	**No. of Participants (%)** [a]		**No. of Events (Event Rate)** [b]	
	**Abatacept**	**Ustekinumab**	**Abatacept**	**Ustekinumab**
	**N = 45**	**N = 46**	**P-Y** [c] **= 29.6**	**P-Y = 36.9**
All Serious Adverse Events	2 (4.4)	5 (10.9)	2 (0.068)	9 (0.244)
Chronic obstructive pulmonary disease	0	1 (2.2)	0	2 (0.054)
Back pain	0	1 (2.2)	0	1 (0.027)
Cholecystitis acute	0	1 (2.2)	0	1 (0.027)
Diabetic ketoacidosis	1 (2.2)	0	1 (0.034)	0
Gastroenteritis	1 (2.2)	0	1 (0.034)	0
Lumbar spinal stenosis	0	1 (2.2)	0	1 (0.027)
Post procedural complication	0	1 (2.2)	0	1 (0.027)
Postoperative wound infection	0	1 (2.2)	0	1 (0.027)
Pulmonary embolism	0	1 (2.2)	0	1 (0.027)
Renal cell carcinoma	0	1 (2.2)	0	1 (0.027)

[a] Number of participants (%) reporting at least one serious adverse event for the preferred term.
[b] Event rate calculated as the number of events / person-years of exposure.
[c] Person-years of exposure calculated as the total follow-up time since randomization across all participants within each treatment group

© 2021 Harris KM et al. et al. *JAMA Dermatology*.

eFigure 2. Time to Psoriasis Relapse (ITT Population)

A. Maximum PASI Improvement of at least 90% (PASI 90) by Week 12

Abatacept

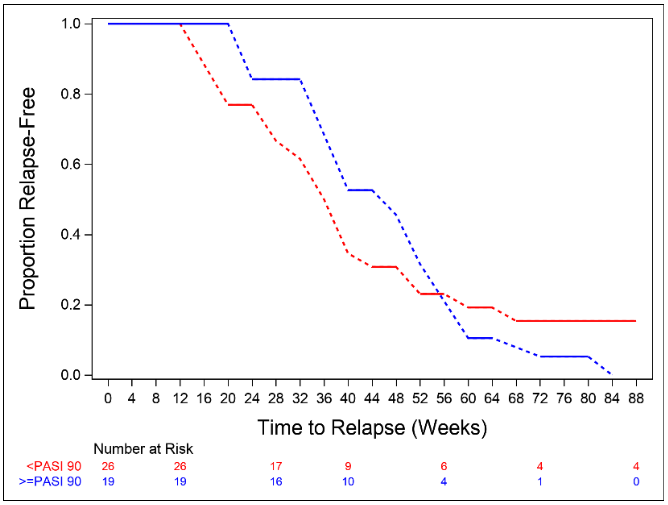

Ustekinumab

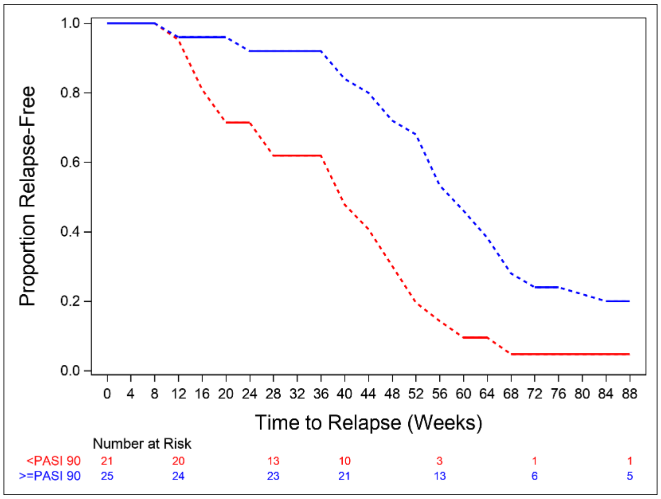

B. Maximum PASI Improvement of at least 90% (PASI 90) by Week 88

Abatacept

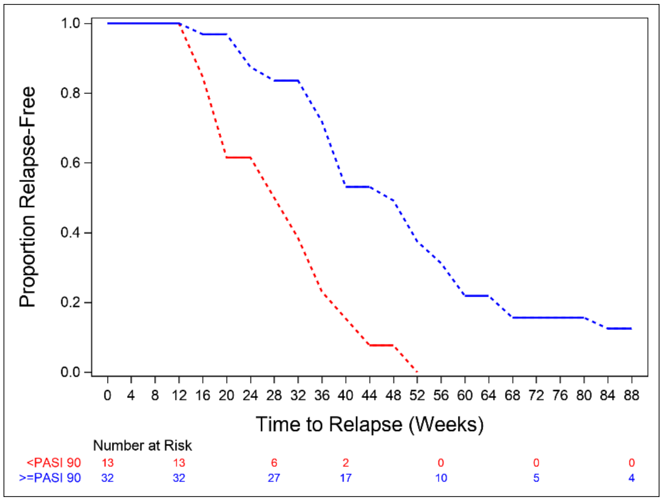

Ustekinumab

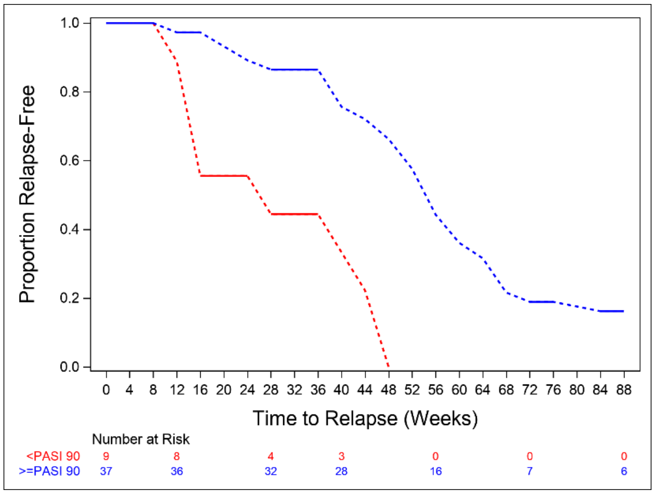

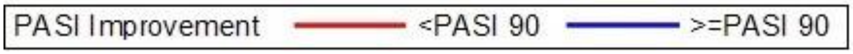

A. Participants who achieved a maximum improvement in PASI scores of at least 90% by Week 12 compared to PASI score at Week 0 (baseline) are indicated as ≥ PASI 90. Participants who did not achieve a maximum improvement in PASI scores of at least 90% by Week 12 compared to PASI score at Week 0 are indicated as <PASI 90. B. Participants who achieved a maximum improvement in PASI scores of at least 90% at any point during study participation (through Week 88) compared to PASI score at Week 0 (baseline) are indicated as ≥ PASI 90. Participants who did not achieve a maximum improvement in PASI scores of at least 90% at any point during study participation compared to PASI score at Week 0 are indicated as <PASI 90.

© 2021 Harris KM et al. et al. *JAMA Dermatology*.

eFigure 3. Modulation of the Disease Transcriptome (PSTR) in Skin and Relevant Cytokines in Serum in Participants Eligible for Randomization (≥PASI75)

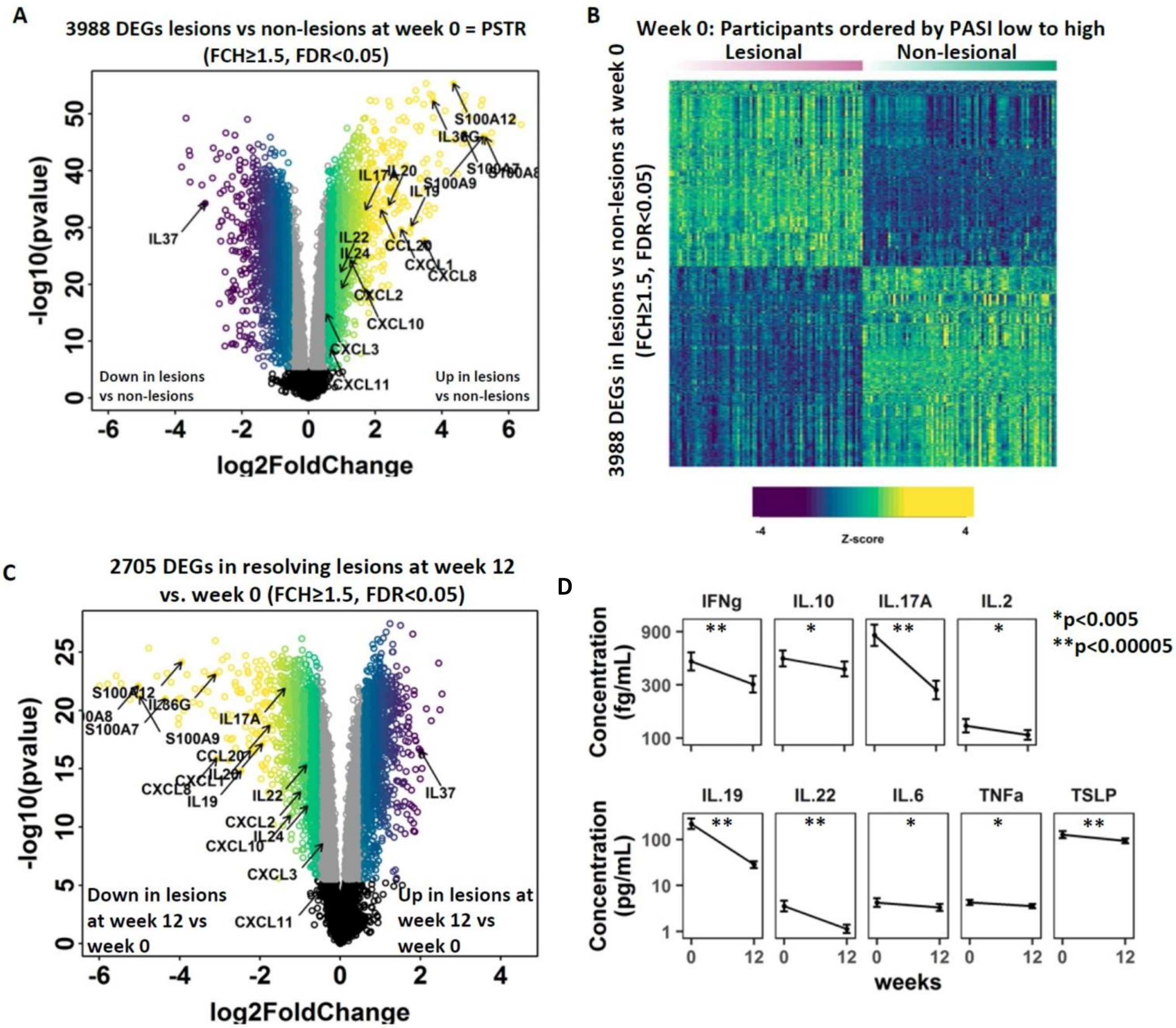

(A) Volcano plot shows the log2 Fold Change of all genes at week 0 in non-leisonal versus lesional skin with genes in the PSTR (FCR≥1.5, FDR<0.05) in color, and highlight the location of IL17A, genes in the pathogenic keratinocyte response pathway, KRT77, IL34, and CCL27. (B) Heatmap shows z-transformed log expression values of DE genes in the PSTR (from A) in paired lesional versus nonlesional skin using the same color gradient in panel A. (C) Volcano plot shows the log2 Fold Change of all genes in resolving lesions at week 12 versus lesions at week 0 with DE genes in colors and highlighted genes from panel A. (D) Line plot shows the mean concentrations of serum cytokines evaluated. Error bars display the 95% confidence intervals. P values determined by paired T-test on log Change from baseline at week 12.

© 2021 Harris KM et al. et al. *JAMA Dermatology*.

eFigure 4. Ustekinumab Modulates Targets of Abatacept in Lesional Skin

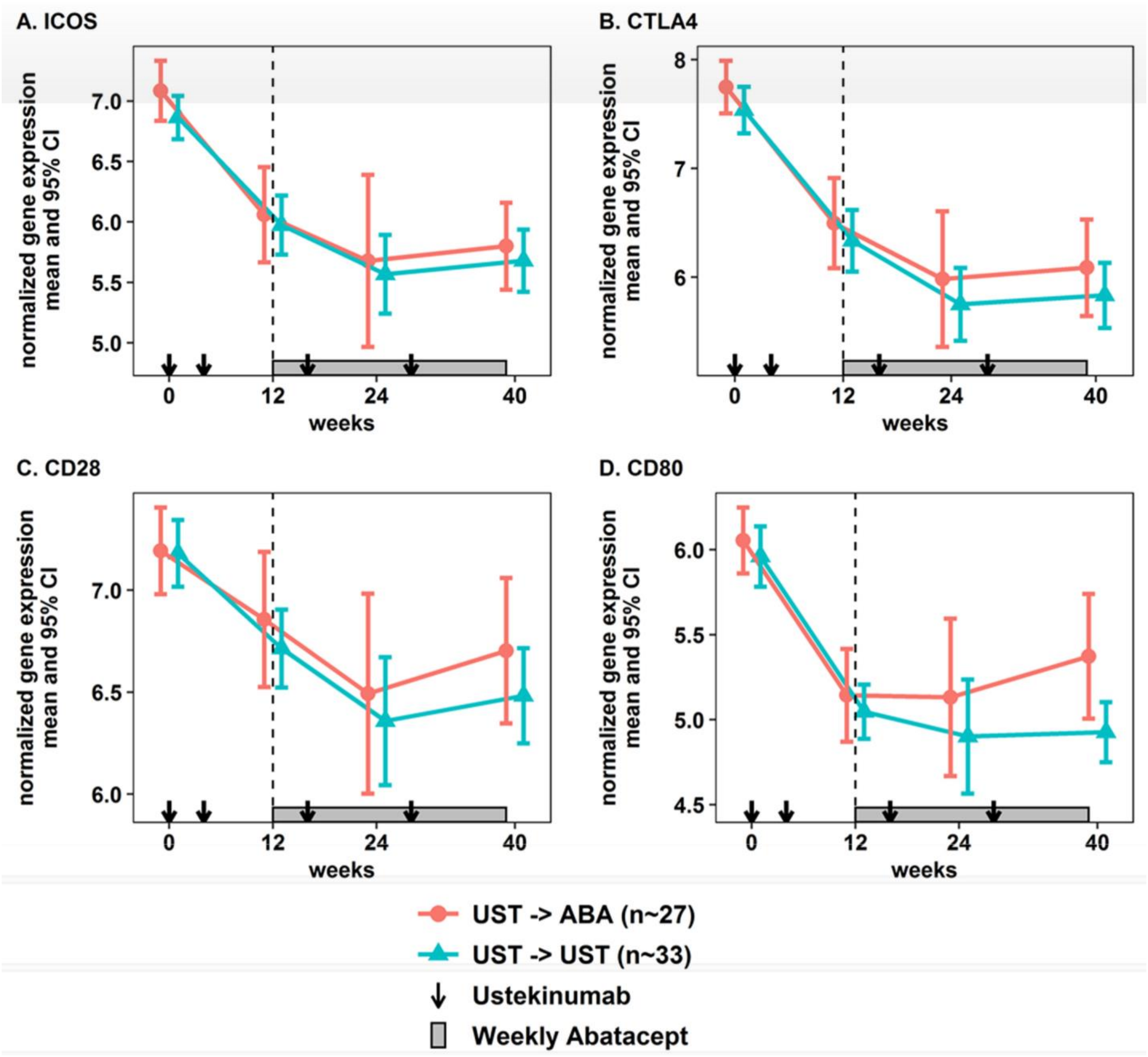

Line plots show the average expression of ICOS (A), CTLA4 (B), CD28 (C) and CD80 (D) genes for lesions at week 0 and resolving lesions at weeks 12, 24 and 40 by treatment group. Dashed vertical line indicates time of randomization. Error bars display the 95% confidence intervals. P values to compare between treatment groups determined by mixed-model for repeated measures with baseline adjustment did not identify significant differences at any time point evaluated. UST->ABA (switched to abatacept group), UST->UST (continued ustekinumab group)

© 2021 Harris KM et al. et al. *JAMA Dermatology.*

eFigure 5. Abatacept Fails to Maintain Suppression of IL17A in Resolving Lesions Following Ustekinumab Withdrawal in Participants That Completed Study Treatment

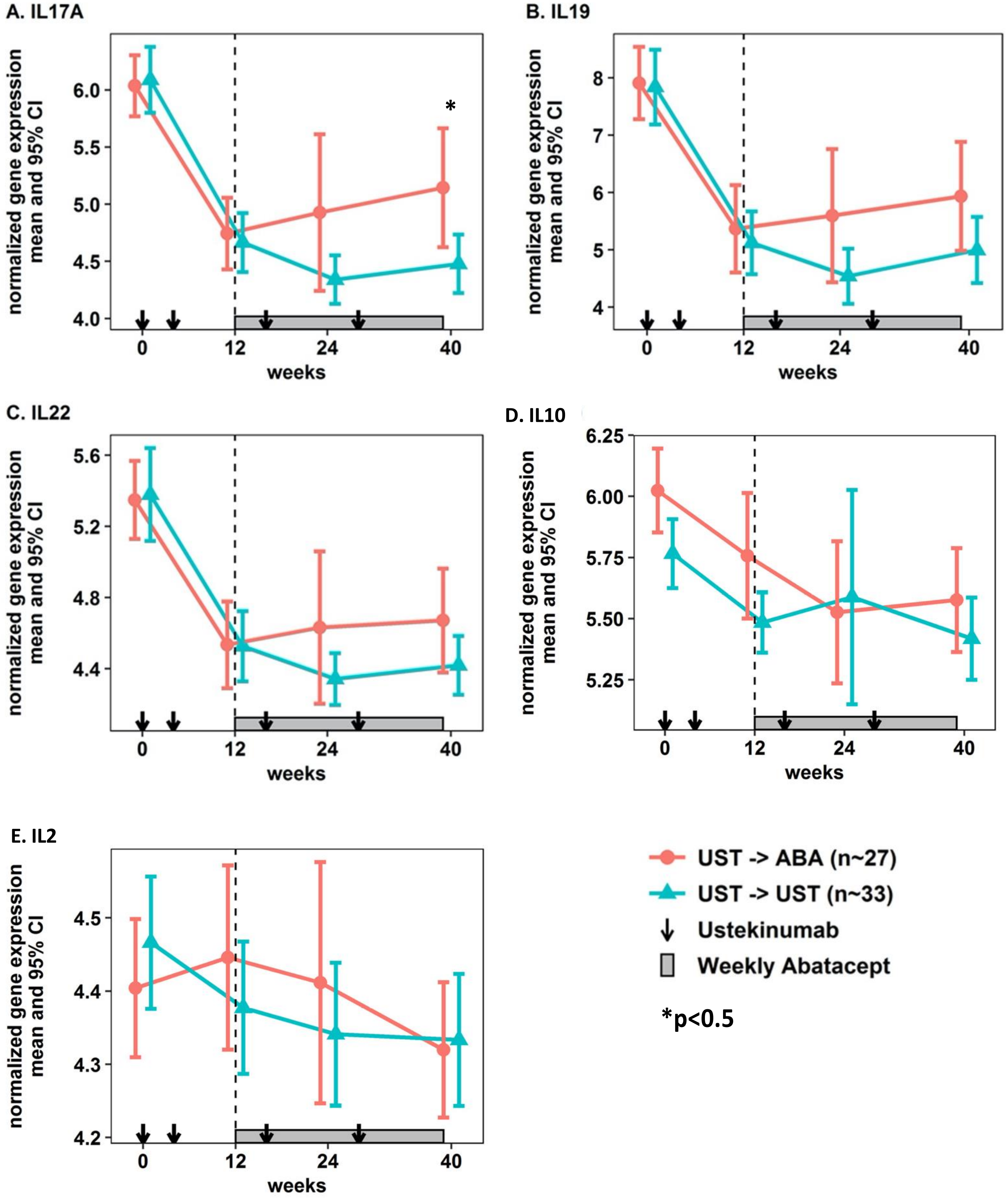

© 2021 Harris KM et al. et al. *JAMA Dermatology*.

Line plots show the average expression of IL17A (A), IL19 (B), IL22 (C), IL10 (D) and IL2 (E) genes for lesions at week 0 and resolving lesions at weeks 12, 24 and 40 by treatment group. Dashed vertical line indicates time of randomization. Error bars display the 95% confidence intervals. P values to compare between treatment groups determined by mixed-model for repeated measures with baseline adjustment. UST->ABA (switched to abatacept group), UST->UST (continued ustekinumab group)

© 2021 Harris KM et al. et al. *JAMA Dermatology*.

eFigure 6. Treatment Effect of Abatacept on Serum IL-10 and IL-2 in PAUSE Is Consistent With ACCLAIM

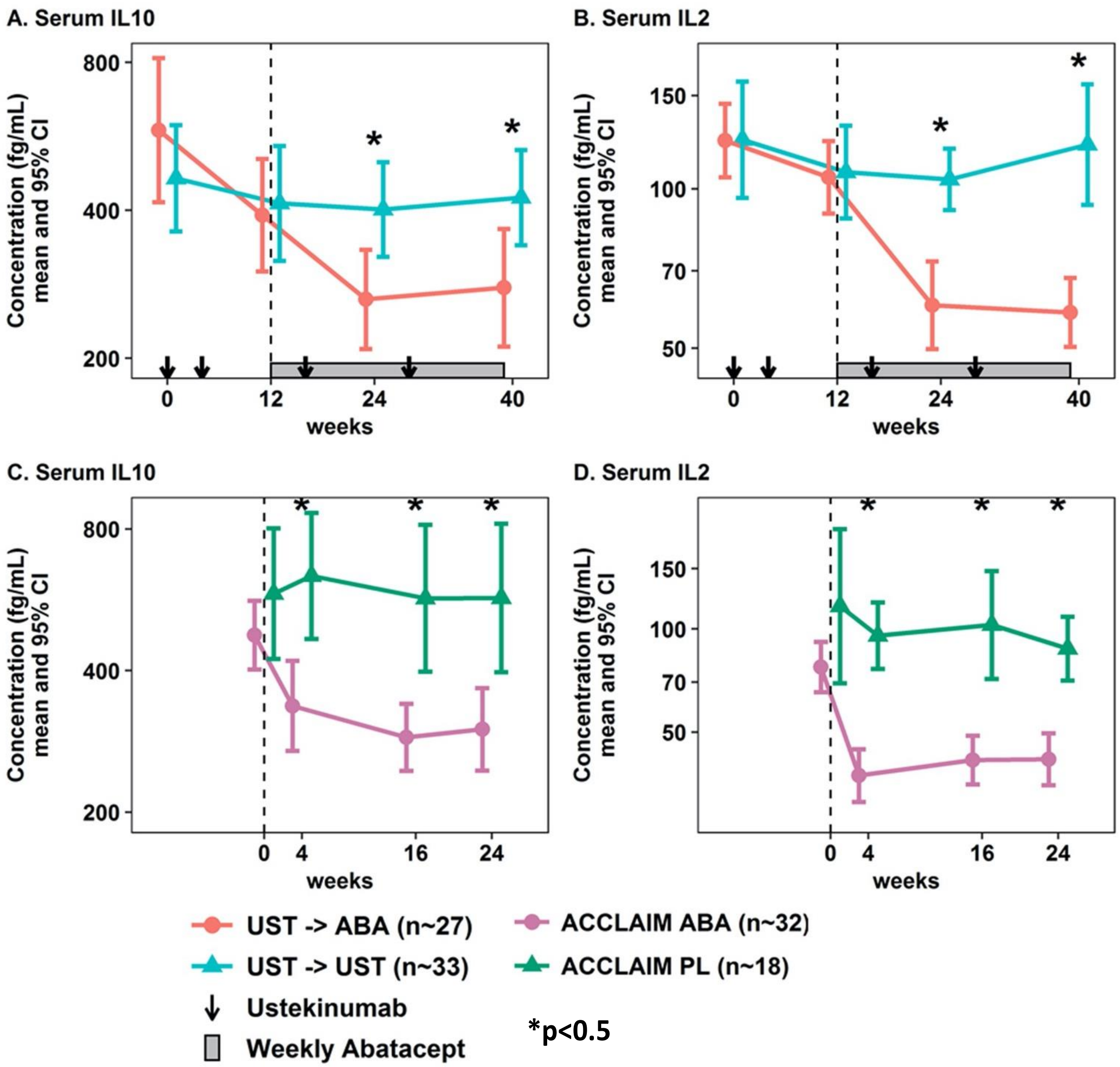

Line plots show the mean concentration of IL-10 (A & C) and IL-2 (B & D) levels in serum from participants that completed blinded treatment in the PAUSE trial (upper panels) or the ACCLAIM trial (lower panels). Dashed vertical line indicates time of randomization. Error bars display the 95% confidence intervals. P values to compare between treatment groups determined by mixed-model for repeated measures with baseline adjustment. UST->ABA (switched to abatacept group) and UST->UST (continued ustekinumab group) from the PAUSE trial; ACCLAIM ABA (abatacept group) and ACCLAIM PL (placebo group) from the ACCLAIM trial.

© 2021 Harris KM et al. et al. *JAMA Dermatology*.

eTable 5. Limits of Blank (LOB) and Lower Limit of Quantitation (LLOQ) of the MSD, LLC Multi-plex Immunoassay

	LOB (fg/mL)	LLOQ (fg/mL)
IL-2	4	13
IL-6	3	17
IL-10	9	76
IL-17A	116	217
IL-22	2	25
IFNg	32	87
TNFa	32	244
TSLP	334	2,632

© 2021 Harris KM et al. et al. *JAMA Dermatology*.
