# Supplementary material for: Effect of Costimulatory Blockade With Abatacept After Ustekinumab Withdrawal in Patients With Moderate to Severe Plaque Psoriasis: The PAUSE Randomized Clinical Trial
Source: JAMA Dermatol. 2021 Oct 13;157(11):1–11. doi: 10.1001/jamadermatol.2021.3492 (PMC8515260; doi:10.1001/jamadermatol.2021.3492)
Supplement: Supplement 5. — Data Sharing Statement [file jamadermatol-e213492-s005.pdf]

Data Sharing Statement

Harris. Effect of Costimulatory Blockade With Abatacept After Ustekinumab Withdrawal in Patients With Moderate to Severe Plaque Psoriasis. *JAMA Dermatol*. Published October 13, 2021. doi:10.1001/jamadermatol.2021.3492

Data
Data available: Yes
Data types: Deidentified participant data, Other (please specify)
Additional Information: Clinical Protocol
How to access data: Data sets and the clinical protocol will be accessible through TrialShare, a public website managed by the Immune Tolerance Network
https://www.itntrialshare.org/PAUSE.url
When available: With publication

Supporting Documents
Document types: None

Additional Information
Who can access the data: Anyone requesting the data
Types of analyses: For any purpose
Mechanisms of data availability: All published data sets will be publicly available through TrialShare to anyone for any purpose after registering for a free account.
Any additional restrictions: No restrictions
